# Exposure of *Mycobacterium marinum* to low-shear modeled microgravity: effect on growth, the transcriptome and survival under stress

**DOI:** 10.1038/npjmgrav.2016.38

**Published:** 2016-12-01

**Authors:** Camille F Abshire, Kanchanjunga Prasai, Israel Soto, Runhua Shi, Monica Concha, Melody Baddoo, Erik K Flemington, Don G Ennis, Rona S Scott, Lynn Harrison

**Affiliations:** 1 Department of Molecular and Cellular Physiology, Louisiana State University Health Sciences Center, Shreveport, LA, USA; 2 Department of Medicine and Feist-Weiller Cancer Center, Louisiana State University Health Sciences Center, Shreveport, LA, USA; 3 Department of Pathology and Tulane Cancer Center, Tulane University Health Sciences Center, New Orleans, LA, USA; 4 Department of Biology, University of Louisiana, Lafayette, LA, USA; 5 Department of Microbiology and Immunology, Feist-Weiller Cancer Center, Louisiana State University Health Sciences Center, Shreveport, LA, USA

## Abstract

Waterborne pathogenic mycobacteria can form biofilms, and certain species can cause hard-to-treat human lung infections. Astronaut health could therefore be compromised if the spacecraft environment or water becomes contaminated with pathogenic mycobacteria. This work uses *Mycobacterium marinum* to determine the physiological changes in a pathogenic mycobacteria grown under low-shear modeled microgravity (LSMMG). *M. marinum* were grown in high aspect ratio vessels (HARVs) using a rotary cell culture system subjected to LSMMG or the control orientation (normal gravity, NG) and the cultures used to determine bacterial growth, bacterium size, transcriptome changes, and resistance to stress. Two exposure times to LSMMG and NG were examined: bacteria were grown for ~40 h (short), or 4 days followed by re-dilution and growth for ~35 h (long). *M. marinum* exposed to LSMMG transitioned from exponential phase earlier than the NG culture. They were more sensitive to hydrogen peroxide but showed no change in resistance to gamma radiation or pH 3.5. RNA-Seq detected significantly altered transcript levels for 562 and 328 genes under LSMMG after short and long exposure times, respectively. Results suggest that LSMMG induced a reduction in translation, a downregulation of metabolism, an increase in lipid degradation, and increased chaperone and mycobactin expression. Sigma factor H (*sigH*) was the only sigma factor transcript induced by LSMMG after both short and long exposure times. In summary, transcriptome studies suggest that LSMMG may simulate a nutrient-deprived environment similar to that found within macrophage during infection. SigH is also implicated in the *M. marinum* LSMMG transcriptome response.

## Introduction

Recent studies have demonstrated that the spacecraft environment can be contaminated with numerous microbial species.^1^ Samples to check for microbes are frequently taken from surfaces, air, and water on the International Space Station (ISS) and tests to identify bacterial species include sequencing of 16s rRNA and culturing viable bacteria.^1,2^ A variety of bacterial phyla have been identified including Actinobacteria, Bacteroidetes, Cyanobacteria, Acidobacteria, Firmicutes, Proteobacteria, SR1, Tenericutes, and TM7 and a number of genera have been identified within each phylum.^1^ The bacteria most frequently found on the ISS include species of *Staphylococcus*, *Bacillus*, and *Micrococcus.*^2^ Experiments to determine the effect of spaceflight or low-shear modeled microgravity (LSMMG) on bacteria have determined that different phenotypes are induced in different types of bacteria. Compared with normal gravity controls alterations to growth, culture density, biofilm formation, aggregation, virulence, and changes in resistance to stress from acid, oxidative stress, heat shock, or antibiotics have been identified.^2–10^ It is not possible to generalize or predict how a bacterial species will react to microgravity or LSMMG. An example is resistance to oxidative stress: exposure to LSMMG increased oxidative stress resistance of *Pseudomonas aeruginosa*^11^ but decreased oxidative stress resistance of *Staphylococcus aureus.*^12^ Changes in the bacterial transcriptome^11–14^ are also induced by microgravity and LSMMG, and changes to the bacterial phenotype are likely due to alterations in the bacterial transcriptome. The diverse phenotypes induced by spaceflight or LSMMG^2–7^ highlights the need to understand more about how different species of bacteria are altered by microgravity and the importance of identifying key molecules that promote survival under the stress of microgravity.

*Mycobacterium marinum* is a member of a family of closely related mycobacteria, most of which are pathogenic. They include *Mycobacterium tuberculosis*, *Mycobacterium avium,* and *Mycobacterium ulcerans.*^15^ Although *M. tuberculosis*, the cause of human tuberculosis, live and replicate only in a host in nature,^15,16^ *M. marinum* and *M. avium* grow in macrophage and aquatic environments.^16^ *M. marinum* grows at 28–35 °C and infects poikilothermic hosts such as fish and frogs, but can infect humans resulting in granulomatous skin lesions, which pathologically are identical to lesions produced by dermal *M. tuberculosis*. The pathogenic *M. avium* complex, which includes the subspecies *avium*, *paratuberculosis,* and *intracellulare*, has been linked with hard-to-treat non-tuberculosis human pulmonary infections in non-immunocompromised^17^ as well as immunocompromised individuals.^18^

*M. marinum*^19^ and *M. avium* form biofilms and can grow in biofilms in water distribution systems,^20^ taps, and showerheads. Thirty percent of biofilms tested from showerheads across the USA contained *M. avium* even though they were linked to municipal water supplies treated with chlorine.^21^ *M. avium* grown in a biofilm is more resistant to chlorine, even when the biofilm is disrupted.^22^ Pathogenic waterborne mycobacteria could pose a potential threat during space travel not only to astronaut health, but also to the spacecraft environment and potable water. In fact, *M. avium* was identified in water samples taken from the Russian Mir space station.^23^

Pathogenic bacteria that have the potential to have increased antimicrobial resistance and virulence are a concern inside spacecraft because astronauts already have a compromised immune system, especially when in space for long periods of time.^24,25^ Uncovering the signaling pathways that result in the altered physiology of bacteria has the potential to reveal new targets for drug development or disinfection that will be essential for long space flights.

There are no published studies on the effect of microgravity or LSMMG on waterborne mycobacteria. Hfq has been identified as a factor regulating microgravity gene expression in *P. aeruginosa,*^13^ *S. aureus*,^12^ and *Salmonella enteric* serovar Typhimurium^14^ (*S. typhimurium*), but mycobacteria do not encode Hfq.^26^ *M. marinum* is closely related genetically to *M. tuberculosis* and *M. avium* and has similar mechanisms of infection and survival inside host macrophage. Although all of these mycobacterium species can grow in specialized medium in the laboratory,^27^ growth of *M. marinum* even at the lower temperature of 30–32 °C is faster than *M. tuberculosis*^27^ and *M. avium.*^28^ *M. marinum* is also a lower risk (bisoafety level 2) to researchers. Hence *M. marinum* is a good model for pathogenic mycobacteria.

The aim of this study was to identify factors involved in the adaptation of *M. marinum* to the LSMMG stressful environment and determine how LSMMG alters mycobacterium physiology. Two strains of *M. marinum* (1218R and LHM4) were used for this study. Strain 1218R was originally isolated from fish and LHM4 was generated from 1218R by integrating a plasmid into the genome that encodes for kanamycin resistance and the red fluorescent protein. Previous work studying the effect of LSMMG on bacteria with doubling times of <1 h exposed the bacteria to LSMMG for 10,^29^ 20,^30^ 24 h^11^ or 10 generations.^31^ *M. marinum* has a doubling time of 6–7 h at 30 °C and hence two exposure times to LSMMG or NG (short ~40 h and long 4 days and ~35 h) were selected to allow the bacteria to undergo multiple replication cycles during the experiments. The exposure protocol was designed so that short and long LSMMG- or NG-exposed cultures were in exponential phase and hence alterations to the transcriptome or resistance to treatment with hydrogen peroxide (H_2_O_2_), acid or gamma radiation were due to different exposure times to LSMMG and not due to different phases of growth.

In summary, this work characterizes the growth, stress survival, and transcriptome of *M. marinum* grown under LSMMG and identifies nutrient deprivation as a signal involved in changes to gene expression and the sigma factor SigH as a factor modulating the LSMMG transcriptome in a waterborne mycobacterium.

## Results

The effect of LSMMG exposure time had to be assessed with bacteria in the same phase of growth, as we found stress resistance altered as the *M. marinum* progressed from early to late exponential phase (data not shown) and it would be expected that the transcriptome of replicating bacteria is very different from bacteria in stationary phase. For the short exposure, *M. marinum* were diluted to an optical density at 600 nm (OD_600_) of 0.002 at the initiation of the high aspect ratio vessel (HARV) culture to make sure that the bacteria would be in exponential phase (OD_600_~0.1–0.2) after ~40 h. This exposure time allowed ~6 generations. For the longer exposure time, *M. marinum* were subjected to 4 days of growth under LSMMG or NG, the bacteria removed from the HARV, re-diluted in fresh medium and the LSMMG or NG exposure continued in new HARVs for a further ~35 h (see Materials and Methods for full details).

### LSMMG did not alter aggregation of *M. marinum*

Previous published studies observed increased aggregation of bacteria exposed to microgravity or LSMMG.^4,12,14^ Mycobacterium under normal laboratory conditions grow as aggregates and standard culture procedure^27,32^ uses Tween 80 in the medium to minimize clumping. Even in the presence of 0.2% Tween 80, *M. marinum* still aggregate. Cultures exposed to NG or LSMMG for various times were examined by microscopy to determine whether exposure to LSMMG altered aggregation. Similar size aggregates were detected in both LSMMG and NG cultures (Supplementary Figure 1A), so growth under LSMMG and standard culture medium did not increase clumping of *M. marinum*. To determine whether the Tween 80 in the culture medium prevented the detection of structure alterations under LSMMG, 1218R and LHM4 were grown initially in a tube with 0.2% Tween 80 until OD_600_ ~1 in the rotating incubator as described in the Materials and Methods section. The cultures were then diluted to OD_600_ 0.002 in growth medium without Tween 80. One dilution for each strain was used to prepare two 50 ml HARVs and a tube culture. The cultures in the tube and HARVs were grown for 67 h (LHM4) or 69 h (1218R). The tube was incubated in the rotating incubator at 120 r.p.m. and the HARVs were grown under NG or LSMMG. The culture in the tube grew as small clumps (data not shown) and clumps were also visible in the HARV cultures. When the culture was removed from the HARVs it was not possible to determine the optical density of the culture as bacteria had adhered to the base of the HARV grown under NG, preventing an accurate measurement. Samples of each culture were examined by microscopy (Supplementary Figure 1B and C) and cording of the mycobacterium was seen in all cultures. This cording structure is known to form in broth as well as biofilms.^19,33^ Under the conditions examined, there was no obvious difference between the NG and LSMMG cultures. Further studies are required to determine whether there are subtle structure differences between the cords or whether cord formation is accelerated under LSMMG. As growth in the absence of Tween 80 prevents accurate measurements of culture density and total recovery of the culture from the HARVs, all further studies were performed in the presence of 0.2% Tween 80.

### *M. marinum* grown under LSMMG transition from exponential phase earlier than NG-exposed *M. marinum*

**Figure 1:**
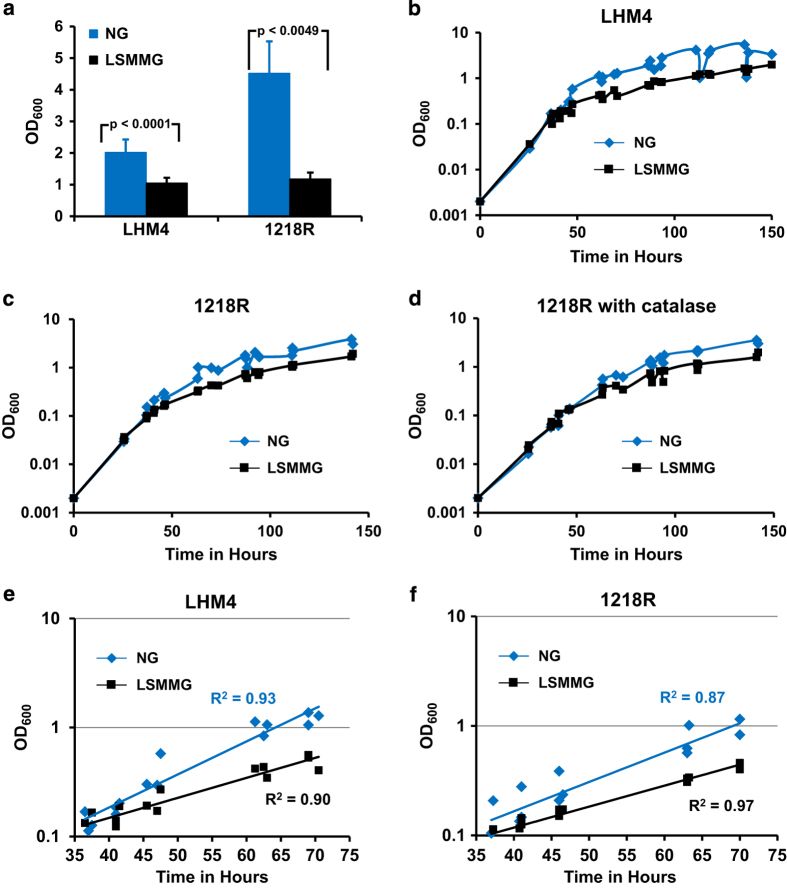
Growth under LSMMG alters culture density of *M. marinum*. LHM4 and 1218R was grown in 10 ml HARVs under NG or LSMMG for 4 days continuously and the OD_600_ determined. The data from 13 HARVs for LHM4 and 3 HARVs for 1218R for each condition was used to obtain the average and s.d. (**a**). OD_600_ was monitored for LHM4 (**b**) and 1218R (**c**) in 50 ml HARVs. At each time point, a small culture volume (1–2 ml) was removed to measure OD_600_ and the volume replaced with fresh medium (2–4% total volume). Strain 1218R was also grown in medium with catalase (**d**). Three independent experiments were performed for each strain and growth condition. As data were not gathered at the exact time point in each experiment, all the data points were pooled for a strain and condition and are shown graphically (**b**–**d**). A transition from exponential phase was detected at ~37 h in LSMMG cultures compared with NG cultures. The data between 37 and 70 h for the three experiments were plotted (**e**, **f**) and regression analysis performed, demonstrating that the increase in OD_600_ during this time period was statistically different when the bacteria were grown under NG and LSMMG (*P*<0.0001).

Thirteen independent cultures of LHM4 and three independent cultures of 1218R were grown continuously for 4 days in 10 ml HARVs under LSMMG or NG and significantly different OD_600_ were obtained for LSMMG compared with NG cultures (Figure 1a). The OD_600_ of the LSMMG culture was reduced by at least 50% compared with the NG culture. To determine in more detail how growth was altered by LSMMG, growth studies of LHM4 (Figure 1b) and 1218R (Figure 1c) were performed over a period of 150 h where the OD_600_ was monitored by removal of a sample of the culture at the different time points. Experiments were performed in triplicate and all data points from these experiments were pooled and plotted to examine the effect of LSMMG on growth (Figure 1b and c). The LSMMG cultures of LHM4 and 1218R had slightly lower OD_600_ compared with the NG culture at ~37 h after culture initiation and this difference increased with time (Figure 1e and f). This was very consistent and detected in all experiments with both *M. marinum* strains. A similar result was found when LHM4 colony forming units per ml (CFU/ ml) was plotted against time (Supplementary Figure 2). As the OD_600_ measurements for the NG cultures began to plateau at around 90 h, indicating an exit from logarithmic growth, the data between 37 and 70 h were considered (Figure 1e and f). Regression analyses determined that during this time period that the data were significantly different (*P*<0.0001) for the LSMMG and NG cultures of LHM4 and 1218R. To examine the extent of change on growth during this transition from exponential phase, the doubling times during this time period were calculated from Figure 1e and f: doubling times were 10 h (LHM4) and 11 h (1218R) for NG cultures, and 15.5 h (LHM4) and 16 h (1218R) for LSMMG cultures. The LSMMG culture did eventually reach a similar culture density to the NG culture at later time points (Figure 1b).

As OD_600_ can be influenced by size of the bacteria, the diameter and length of 50 bacteria in an LHM4 culture grown for 4 days under LSMMG (OD_600_=1.3) or NG (OD_600_=2.3) were determined. No statistical significant difference was found for the diameter (NG=0.72±0.05 μm, LSMMG=0.73±0.07 μm) or the length (NG=2.7±0.45 μm, LSMMG=2.7±0.5 μm) between the two cultures.

Published studies have determined that LSMMG induces oxidative stress genes in bacteria;^30^ suggesting LSMMG disturbs the normal redox environment. Catalase breaks down H_2_O_2_ and is present in standard *M. tuberculosis* medium (3 μg/ml). Growth experiments for 1218R were therefore performed with medium containing catalase (3 μg/ml) to remove extracellular H_2_O_2_. No change in growth was detected when the medium contained catalase, which suggests extracellular H_2_O_2_ did not hinder growth under LSMMG (Figure 1d).

### *M. marinum* transcriptome response to LSMMG

RNA samples isolated from triplicate independent cultures of 1218R grown under LSMMG or NG cultures for the short or long exposure times were used for RNA-Seq analyses, and 6–10 million reads were sequenced for each biological sample (Supplementary Table 1). RNA-Seq using 5–10 million non-rRNA reads has previously been found to provide sufficient depth of sequencing to detect the majority of alterations in bacterial gene expression.^34^ Following transcript quantification a comparison of LSMMG with NG after short or long exposure times (Supplementary Excel Worksheets 1 and 2) identified 562 and 328 genes, respectively, with significantly altered transcript levels. From the 10 categories of mycobacterial genes (http://mycobrowser.epfl.ch/marinolist.html), the intermediary metabolism and respiration category had the greatest number of genes with significant transcript changes (Supplementary Table 2).

To determine the genes with the greatest physiological relevance to growth under LSMMG the real fold change (RFC) was calculated (Supplementary Excel Worksheets). Reverse-transcriptase quantitative PCR (RT-qPCR) was also performed for 9 different genes and the majority of the LSMMG/NG fold changes in expression determined by RNA-Seq and RT-qPCR were similar (Supplementary Table 3). The genes with the greatest RNA-Seq RFC (top 10%) after short and long exposure times are shown in Supplementary Tables 4 and 5.

**Figure 2:**
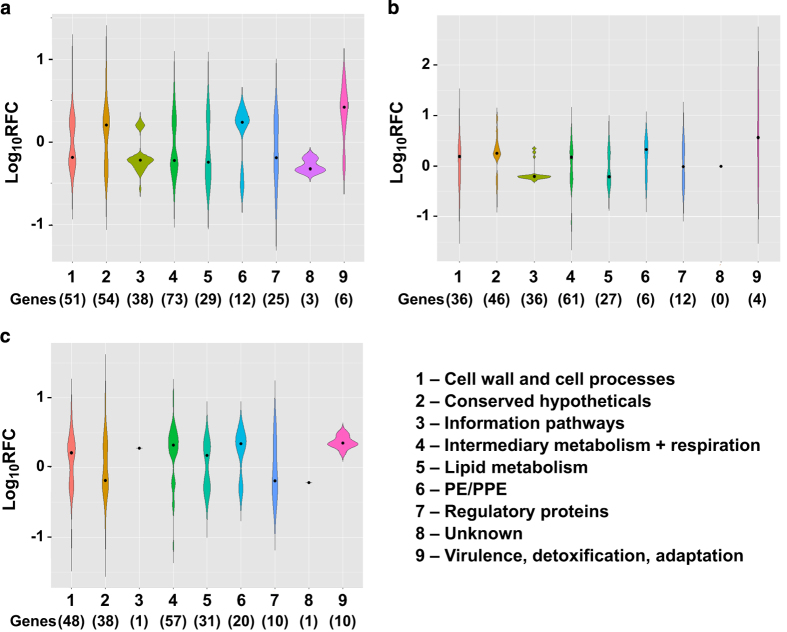
Gene expression changes identified by RNA-Seq. Transcriptional profiles were compared between 1218R grown at LSMMG and NG for 40 h (**a**, LSMMG/NG) or 4 days ~35 h (**b**, LSMMG/NG) and between 1218R grown under LSMMG for the two time periods (**c**, 4 days ~35 h/40 h). The significant (*P*<0.05) changes in transcript levels were identified. The real fold change (RFC) is the ratio of the normalized mean count values for LSMMG divided by the normalized mean count values for NG. The genes that changed ⩾1.5-fold were assigned to categories (1–9) and the distribution of the RFC is shown graphically. No genes were significantly altered ⩾1.5-fold in the insertion sequences and phage category and so this category is not included. The mean value for the RFC for a category is marked by the black spot and the number of genes in the category is shown in parentheses.

Genes that changed expression ⩾1.5-fold (RFC⩽0.67 or ⩾1.5) by RNA-Seq were identified and the log_10_ RFC plotted for the genes according to category (Figure 2). The category with the greatest number of genes for LSMMG versus NG at each exposure time (Figure 2a and b) was intermediary metabolism, and there was an even distribution of genes with increased and decreased expression. The categories of genes relating to the cell wall and the conserved hypotheticals had the next highest number of genes that significantly changed. Even though these categories also had an even distribution of genes with increased and decreased expression (Figure 2a and b), some genes relating to these two categories had the highest RFC, especially after a short exposure to LSMMG (Supplementary Tables 4 and 5). Unfortunately the function of the conserved hypothetical genes is unknown. The majority of genes involved in the information pathway and lipid metabolism categories decreased expression, whereas the genes in the PE/PPE and virulence categories increased expression (Figure 2a and b). There were also genes in the regulatory protein category that were significantly changed by LSMMG, but genes were again evenly distributed for increased and decreased expression. When the data from the LSMMG samples for short versus long exposure times were statistically compared, it was evident that gene expression continued to change with greater exposure to LSMMG (Figure 2c), and the categories for cell wall, intermediary metabolism, PE/PPE, and virulence increased expression.

To identify genes involved in adaptation to LSMMG, genes were considered that significantly changed ⩾1.5-fold at both the short and long exposure time points (Table 1). The changes for the majority of these 97 genes were very similar at the two time points. Transcript changes include a ~2-fold decrease for ribosomal proteins and a decrease for most of the genes in categories related to lipid metabolism and intermediary metabolism (Table 1). The greatest changes were in genes encoding chaperone proteins.

### Expression of cell metabolism genes

Groups of genes linked with specific metabolic pathways were examined using hierarchical clustering in GeneSifter to determine whether gene expression changes were linked to growth under LSMMG. Hierarchical clustering of all the LSMMG samples suggests changes in gene expression were linked to LSMMG. Genes involved in glycolysis (12 genes), electron transport chain (ETC, 87 genes), pentose phosphate pathway (7 genes), gluconeogenesis (15 genes), methylcitrate cycle (10 genes), glyoxylate cycle (14 genes), and citric acid cycle (TCA, 19 genes) were examined. The majority of these pathways had few significant changes or changes not related to LSMMG (data not shown). An example is ETC, where 22 genes had significant alterations but the changes were not linked to growth under LSMMG (Supplementary Figure 3A). However, 17/19 TCA genes had altered expression linked to LSMMG (Supplementary Figure 3B). Transcript levels for aconitase decreased after short exposure to LSMMG. Succinate dehydrogenase is also a TCA enzyme, but also forms complex II of the ETC.^35,36^ There are two forms of succinate dehydrogenase (Sdh1 and Sdh2) and transcript levels of 3 (*sdhB-D*) of the four genes encoding Sdh2 decreased at the short exposure time point, while transcripts from *sdhB* and *sdhD* decreased after long exposure. There was no change in expression of the genes encoding Sdh1. Proline oxidation can also donate electrons to ETC.^36^ Transcripts from *MMAR_4252* (*putA,* proline dehydrogenase) and *MMAR_4253* (pyrroline-5-carboxylate dehydrogenase) were increased 2–4-fold by LSMMG after the short exposure time (Supplementary Worksheets 1 and 2). After the long exposure time, only *MMAR_4252* was increased 2.6-fold (Table 1). Isocitrate lyase (Icl) is an enzyme that connects the TCA and glyoxylate cycle and transcript levels from *icl* (*MMAR_0792*) decreased by ~10-fold in LSMMG bacteria after the long exposure time (Supplementary Table 5).

**Figure 3:**
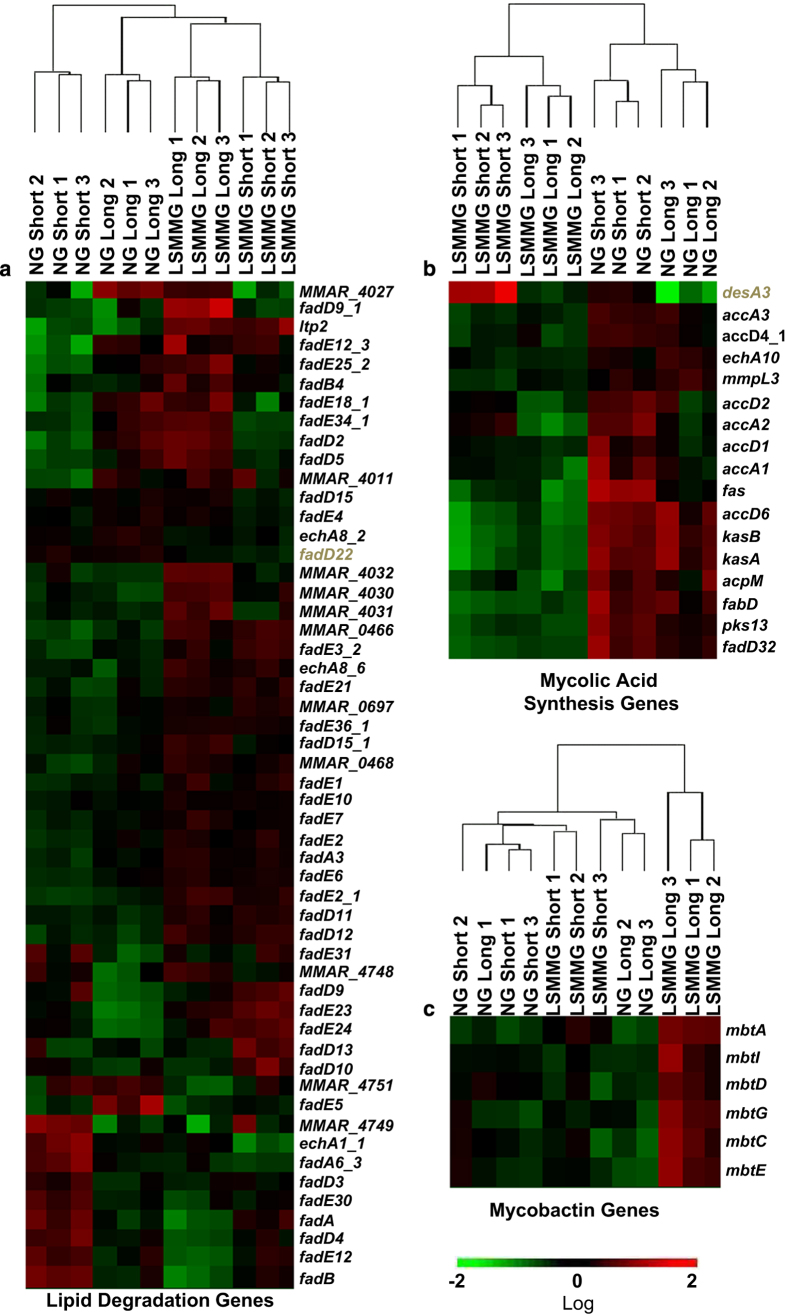
Growth under LSMMG increases expression of genes involved in lipid degradation and mycobactin synthesis but decreases expression of genes involved in mycolic acid synthesis. Genes involved in the designated categories lipid degradation (**a**), mycolic acid synthesis (**b**) and mycobactin synthesis (**c**) were identified using the mycobrowser website (http://mycobrowser.epfl.ch/) and the literature.^38^ The FPKMs (fragments per kilobase of transcript per million reads) for each gene in the 12 RNA samples were analyzed using Genesifter. Only genes with ⩾1.5-fold change in expression (*P*<0.05) were considered. The data were log transformed. The heat maps were generated showing expression in short (growth for ~40 h) and long (growth for 4 days ~35 h) exposure samples. Genes colored brown are discussed in the manuscript text.

Transcription of genes involved in lipid degradation and catabolism of fatty acids were altered significantly in LSMMG bacteria. We identified 104 genes in *M. marinum* involved in lipid degradation and 53 had ⩾1.5-fold significant change in transcript levels after growth under LSMMG (Figure 3a). Of the 53 genes, 16 had decreased expression and 37 had increased expression, the majority of which encode enzymes involved in fatty acid β oxidation. Fatty acids are also used for polyketide lipid synthesis of phenolic glycolipids and phthiocerol dimycocerosates, mycolic acid (MA) synthesis, production of cell membrane phospholipids, as well as production of carbon stores in the form of triacylglycerol.^37^ Although there were no alterations in expression of genes encoding triacylglycerol synthase (*MMAR_2634, MMAR_2598, MMAR_1519 and MMAR_1157*), genes involved in polyketide lipid synthesis (*fadD22* and *pks15/1*) were decreased 1.5- to 2-fold (Supplementary Excel Worksheets 1 and 2) and multiple genes^38^ encoding proteins involved in MA synthesis were also decreased (Table 1 and Figure 3b). Two genes (*desA3* and *desA3_2*) encoding membrane-bound desaturases showed increased expression at both time points in LSMMG bacteria (Table 1 and Figure 3b). These desaturases are involved in oleic acid production and potentially the production of cis double bonds in meromycolic acids.^38^

Mycobactins chelate iron from the environment and are also known to be important for mycobacterial growth during infection of macrophage.^39^ Sixteen genes involved in mycobactin synthesis were identified and six increased expression after LSMMG exposure for 4 days and ~35 h (Figure 3c).

### SigH: a possible regulator of the mycobacteria LSMMG response

**Figure 4:**
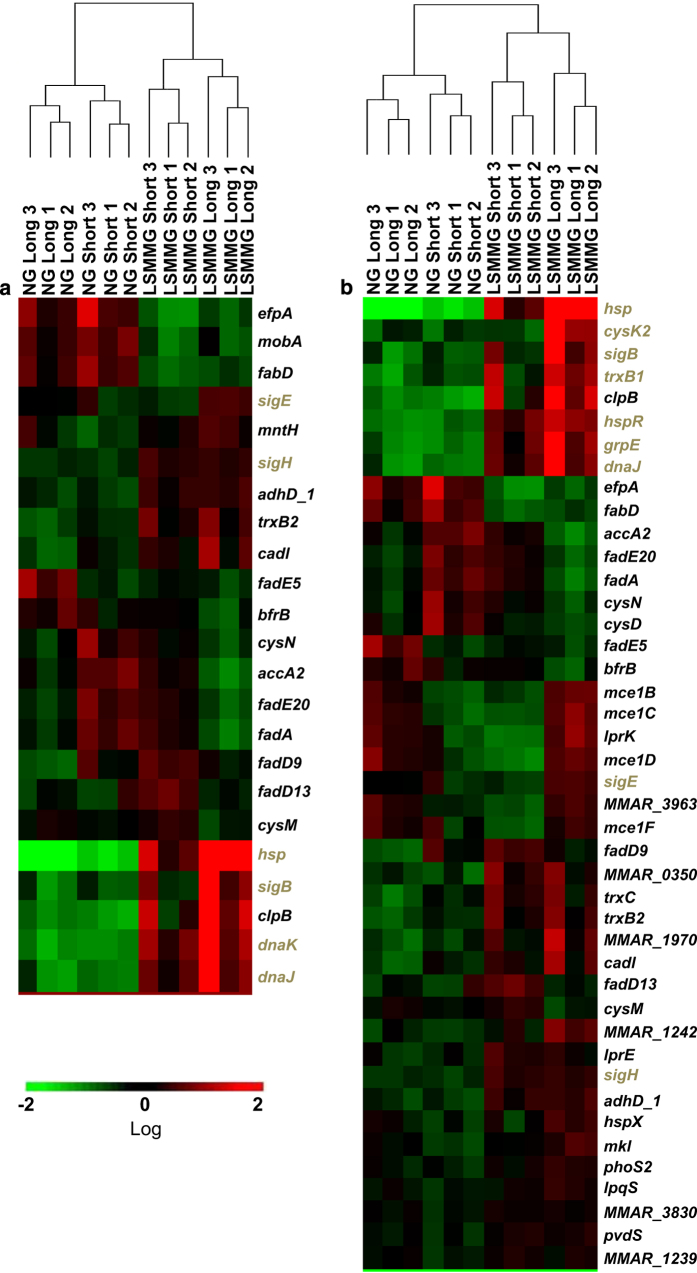
Changes in expression of SigH-regulated genes show a linkage to growth under LSMMG. Mehr *et al.*^41^ treated wild-type and *sigH* mutant *M. tuberculosis* with diamide and determined SigH-related gene expression changes after 0 and 30 min recovery time. Peak *sigH* transcript was at 30 min. *M. marinum* orthlogs of these genes were identified (http://mycobrowser.epfl.ch/). The FPKMs (fragments per kilobase of transcript per million reads) for each gene in the 12 RNA samples were analyzed for hierarchical clustering using GeneSifter with a threshold of 1.5-fold change and a statistical difference (*P*<0.05) determined using an ANOVA. The data were log transformed. The heat maps show expression changes during LSMMG and NG for the orthologs of the *M. tuberculosis* genes that changed after 0 (**a**) and 30 min (**b**). Genes colored brown are discussed in the text.

*sigH* transcript levels increased 1.5-fold at both time points (Table 1). SigH is known to regulate chaperone encoding genes^40,41^ (*hsp* and the *dnaK*, *dnaJ*, *grpE* and *hspR* operon) and *hsp* transcript levels increased at both time points (Table 1). Transcripts from all the *dnaK* operon genes increased after ~40 h of LSMMG exposure (Supplementary Table 4 and Excel Worksheet 1), while only transcripts from *dnaK* and *hspR* increased after LSMMG exposure for 4 days and ~35 h (Supplementary Table 5 and Excel Worksheet 2). *M. tuberculosis* SigH directly/indirectly regulated genes have been identified in a study^41^ where wild-type and *sigH* mutant *M. tuberculosis* were allowed to recover after diamide treatment: RNA analysis identified 44 genes at 0 min and 91 genes at 30 min of recovery as SigH-regulated genes. We identified the *M. marinum* orthologs of these genes (40 at 0 min and 78 at 30 min) and using GeneSifter determined that >50% of these orthologs altered expression significantly after growth under LSMMG (Figure 4).

### Changes in expression of genes related to the enduring hypoxic response and the nutrient starvation response

**Figure 5:**
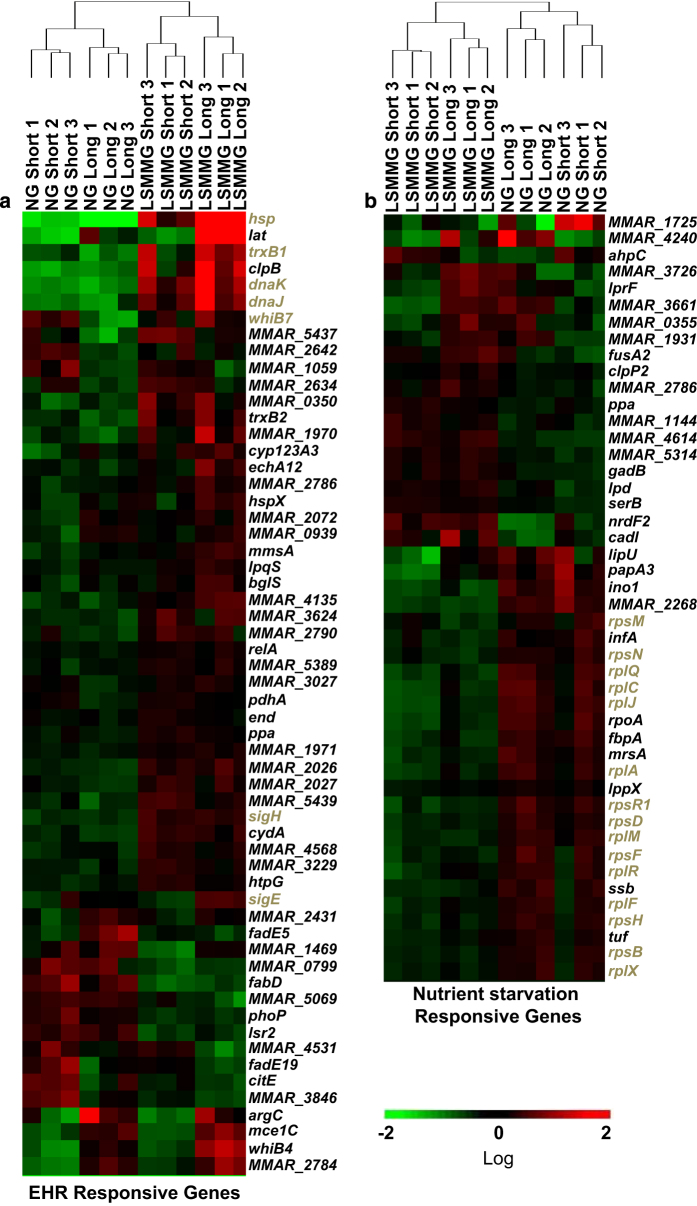
The effect of LSMMG on genes involved in the enduring hypoxic response (EHR) or nutrient starvation response. Genes involved in these two stress responses have been identified in *M. tuberculosis.*^42,43^ The *M. marinum* orthologs were identified (http://mycobrowser.epfl.ch/) for the EHR (**a**) and *relA* nutrient starvation (**b**) response^43^ (Supplemental Data Set 1 and section 3 in ref. 43). The FPKMs (fragments per kilobase of transcript per million reads) for each gene in the 12 RNA samples were analyzed for hierarchical clustering using GeneSifter with a threshold of 1.5-fold change and a statistical difference (*P*<0.05) determined using an ANOVA analysis. The data were log transformed. Genes colored brown are discussed in the text.

The enduring hypoxic response (EHR)^42^ and the *relA* nutrient starvation response^43^ are well characterized in *M. tuberculosis*. We identified 147 *M. marinum* orthologs from the 230 *M. tuberculosis* genes involved in EHR, and 105 *M. marinum* orthologs from the 147 *M. tuberculosis* genes involved in the nutrient starvation response. These orthologs (58 for EHR and 46 for nutrient starvation response) were also found to significantly alter expression⩾1.5-fold due to LSMMG (Figure 5). The EHR genes that overlap with the LSMMG changes in *M. marinum* (Figure 5a) include *sigH* and SigH-regulated genes. For *M. tuberculosis*, all the genes in Figure 5a increase expression during EHR, whereas 25 increased transcript levels under LSMMG. A major group of overlapping genes between the nutrient starvation response in *M. tuberculosis* and the LSMMG response in *M. marinum* are related to translation. The nutrient starvation response includes 23 translation-related genes and 22 decrease expression during the response. There are 21 corresponding *M. marinum* orthologs, 17 changed expression in *M. marinum* under LSMMG and 16 decreased expression (Figure 5b and Table 1).

### Comparison of *M. marinum* exposed to LSMMG and *P. aeruginosa* exposed to spaceflight or LSMMG

During spaceflight, 167 genes were differentially regulated in *P. aeruginosa* and Hfq was identified as a transcription regulator of the bacterial response to microgravity^13^ and LSMMG.^11^ Mycobacteria do not have a gene encoding Hfq, but there is overlap in the lists of genes that are differentially regulated in *P. aeruginosa* exposed to spaceflight or LSMMG and *M. marinum* subjected to LSMMG (Supplementary Tables 6 and 7). A hypergeometric distribution test was performed to determine whether the number of genes found to overlap were significant or could have occurred by chance. The number of protein coding sequences in *M. marinum* is 5,452 (refs 15,44) and from the short exposure to LSMMG there were 562 significant changes, of which 38 overlapped with *P. aeruginosa* exposed to spaceflight (where 167 changes were detected) and 47 overlapped with *P. aeruginosa* exposed to LSMMG (where 330 changes were detected). From the long exposure of *M. marinum* to LSMMG, there were 328 significant changes, of which 38 overlapped with *P. aeruginosa* exposed to spaceflight and 34 overlapped with *P. aeruginosa* exposed to LSMMG. The number of overlapping genes in each case was found to be significant (short exposure *M. marinum* and spaceflight *P. aeruginosa* representation factor 2.2, *P*<1.513e^−6^, short exposure *M. marinum* and LSMMG *P. aeruginosa* representation factor 1.4, *P*<0.012, long exposure *M. marinum* and spaceflight *P. aeruginosa* representation factor 3.8, *P*<2.17e^−13^, long exposure *M. marinum* and LSMMG *P. aeruginosa* representation factor 1.7, *P*<0.001).

When comparing the LSMMG *M. marinum* data with the spaceflight data from *P. aeruginosa*, the largest overlap in downregulated genes encode ribosomal proteins and other common downregulated genes produce proteins involved in transcription and translation. Genes encoding the metabolic enzymes Icl and Sdh2 are also downregulated in both organisms, suggesting similar downregulation of metabolic pathways. Interestingly there were no genes with decreased transcript levels detected for *P. aeruginosa* grown under LSMMG, but 330 transcript levels increased by ⩾1.5-fold.^11^ From this list a number of transcripts encoding proteins of similar function were increased in *P. aeruginosa* subjected to LSMMG but decreased in LSMMG-grown *M. marinum* (Supplementary Table 7). These include transcripts for 13 ribosomal proteins, Sdh2 (*sdhB* and *sdhD*), Icl, RNA polymerase alpha subunit (*rpoA*) and elongation factor Ts (*tsf*). A common group of genes with increased transcript levels in LSMMG *M. marinum* and *P. aeruginosa* were the stress response genes for heat shock and chaperone proteins.

Transcriptome data therefore suggest that *M. marinum* grown under LSMMG exhibit similar stress responses as LSMMG-grown *P. aeruginosa*, but have transcriptional, translational, and metabolic alterations similar to spaceflight-exposed *P. aeruginosa*.

### Survival of LHM4 NG- and LSMMG-exposed cultures to stressful conditions

**Figure 6:**
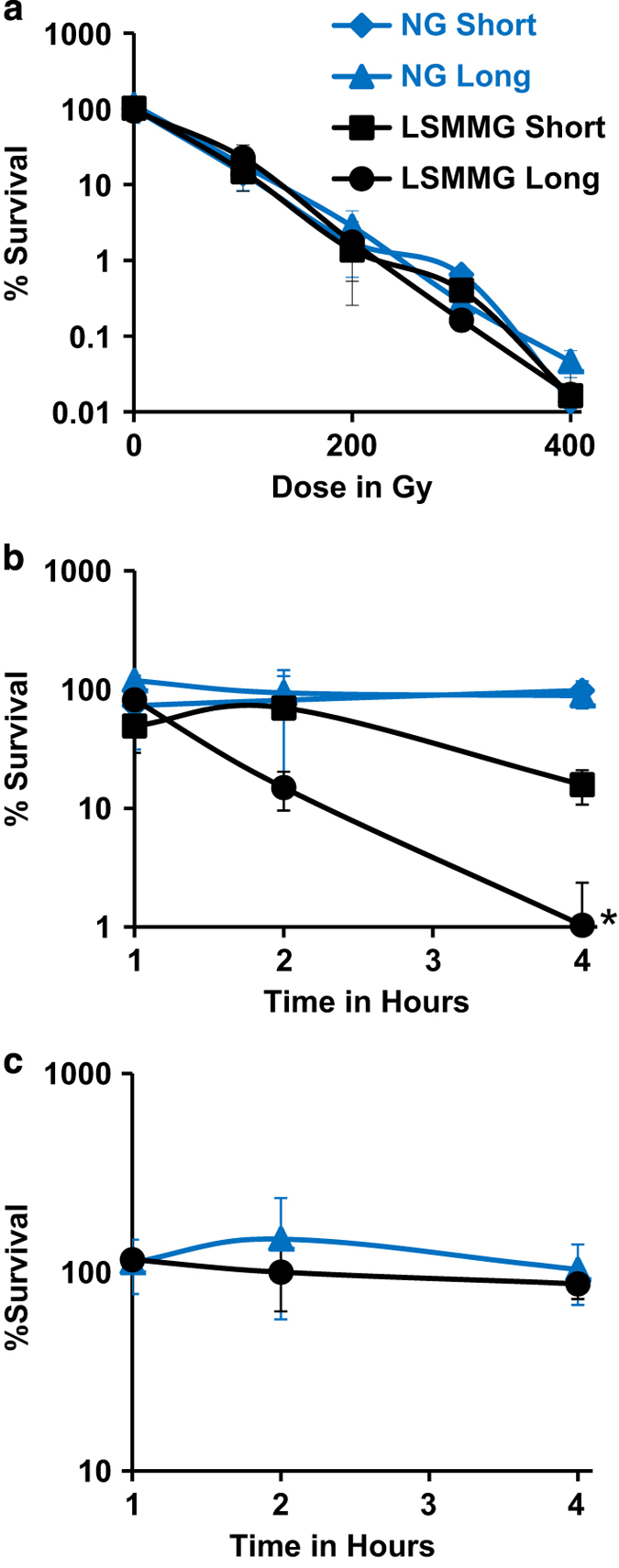
LHM4 grown under LSMMG are more sensitive to H_2_O_2_ but not gamma radiation or acid in comparison with NG cultures. Bacteria were grown in HARVs under LSMMG or NG for ~40 h (short exposure time) or 4 days ~35 h (long exposure time). Cultures were removed and treated with gamma radiation using a ^137^Cesium source (**a**), H_2_O_2_ (**b**), or growth medium at pH 3.5 (**c**). Bacteria were grown on solid medium and colonies counted. The percent survival was calculated by dividing the colony number/ml by that obtained for a culture treated with no dose at the same time. At least three experiments were performed for each culture and treatment type, except for the short exposure time LSMMG and NG cultures treated with H_2_O_2_ where two experiments were performed. The average and s.d. are shown. Survival was compared between the LSMMG and NG samples using the area under the curve and the only statistically significant difference was between the long exposure LSMMG culture and the long exposure NG culture treated with H_2_O_2_ (*P=0.004).

Ionizing radiation and H_2_O_2_ produce an oxidizing environment in cells. Compared with NG, LSMMG-exposed bacteria showed no change in resistance to ionizing radiation (Figure 6a), but were more sensitive to H_2_O_2_ (Figure 6b) with the sensitivity increasing with longer exposure to LSMMG. LHM4 exposed to NG and LSMMG for 4 days and ~35 h were also tested for sensitivity to pH 3.5 for up to 4 h and no cell death occurred (Figure 6c).

## Discussion

Previous studies examining liquid bacterial growth during space travel^3^ or LSMMG^2,3^ have found species-specific alterations: LSMMG-grown *S. typhimurium* had decreased doubling time in minimal medium,^29^ *Escherichia coli* and *Bacillus subtilis* reached higher cell densities and had increased growth rate during spaceflight compared with ground controls,^45^ while *S. aureus* achieved higher cell densities in the normal gravity control compared with the LSMMG culture.^12^ In our study, in a 4-day continuous growth culture, *M. marinum* subjected to NG achieved a higher cell density than the LSMMG culture. As the size of the bacteria did not change and aggregates were disrupted before optical density measurements were determined, the lower LSMMG cell density was likely due to an alteration in growth. A more detailed examination of the growth indicated an early transition of the LSMMG bacteria from exponential phase resulting in a slowing of growth. Even though RNA-Seq analysis was performed at the point where this transition was just detectable, the slowing of growth is apparent from changes in the transcriptome. MA synthesis is linked to new membrane synthesis and cell wall expansion^38^ and is needed for bacterial cell division, and MA synthesis gene transcript levels decreased ~3-fold under LSMMG. During exponential growth there is also a linear relationship between bacterial growth rate and the number of ribosomes in a cell.^46^ The slower growing LSMMG *M. marinum* culture had ~2-fold reduction in transcripts of multiple ribosomal proteins, indicating a downregulation of translation. *P. aeruginosa*^13^ and *S. typhimurium*^14^ cultured during spaceflight also had decreased ribosomal protein transcripts, but growth studies were not performed for these bacteria.

### Transcriptome changes imply a nutrient-deprived environment under LSMMG

Forty-six *M. marinum* orthologs of the *M. tuberculosis relA* nutrient starvation response genes^43^ had expression changes linked to LSMMG. Nutrient starvation results in decreased growth rate, translation, lipid biosynthesis (e.g., MA synthesis), and downregulation of metabolism.^43,47^ These phenotypes were also implied from the transcriptome data for LSMMG-exposed *M. marinum*.

Key bacterial metabolic pathways include β-oxidation of fatty acids, ETC, TCA, and the methylcitrate and glyoxylate cycles.^35^ In LSMMG *M. marinum*, a number of genes encoding enzymes involved in lipid degradation increased expression, but *icl* transcript levels decreased ~10-fold after long exposure to LSMMG. Icl converts isocitrate into glyoxylate, and glyoxylate combined with acetyl CoA from β oxidation of fatty acids produces malate for the glyoxylate cycle.^35^ Also transcript levels decreased 3–6-fold in LSMMG *M. marinum* for the WhiB3 transcription regulator, which is implicated in the adaptation of mycobacteria to growth on long-chain fatty acids.^48^ The transcriptome data therefore suggest energy generation from fatty acids was depressed in LSMMG *M. marinum*.

Decreased transcript levels of genes encoding Sdh2 and aconitase suggest that ETC, TCA, and the methylcitrate and glyoxylate cycles are depressed in LSMMG *M. marinum*. Sdh2 is an enzyme that is essential for growth and Sdh1 cannot substitute for all activities of Sdh2 (ref. 49), which links oxidative phosphorylation energy production to carbon metabolism.^36^ Succinate oxidation generates electrons for ETC, but in mycobacteria proline oxidation can substitute for succinate oxidation under carbon starvation and slow growth conditions.^36^ The transcriptome data indicate that *M. marinum* increased proline oxidation under LSMMG; transcript levels increased for proline oxidation enzymes when transcript levels from the Sdh2 genes decreased. Sdh1 and 2 levels in *Mycobacterium smegmatis* have been linked to energy availability and oxygen levels: under energy-limiting conditions Sdh2 is downregulated and Sdh1 is upregulated, and this is reversed under oxygen-limiting conditions.^36^ Even though in our study, the genes encoding Sdh1 did not alter expression significantly, the transcript changes for Sdh2 and the proline oxidation enzymes suggest that LSMMG *M. marinum* could still produce energy by ETC, and were sensing a carbon-starved/energy-limiting environment.

Taken together, the slower growth and the transcriptome data relating to translation and metabolism suggest that LSMMG simulates nutrient deprivation conditions in *M. marinum*. Fluid dynamics in cultures under microgravity or LSMMG are different from NG cultures.^8,11^ Crabbé *et al.*^11^ examined fluid mixing in HARVs subjected to NG and LSMMG by adding a drop of crystal violet to the fluid-filled vessels. This experiment demonstrated different mixing rates under the two conditions: even after 12 h the crystal violet had dispersed only around the circumference of the HARV subjected to LSMMG, while complete dispersal had occurred under NG. This decreased mixing and low fluid shear under LSMMG and the fact that *M. marinum* are non-motile in liquid culture results in the environment immediately around the bacterium being influenced more by diffusion. Under LSMMG, diffusion is likely the main method moving nutrients to and waste products away from the bacterium. The fluid dynamics in the LSMMG culture could therefore generate a local nutrient-deprived condition. This idea is also supported by increased transcript levels of the WhiB7 transcription regulator and the mycobactin synthesis genes after long exposure to LSMMG. *whiB7* is induced by nutrient starvation and growth under low-iron conditions,^50,51^ while mycobactin synthesis genes are induced under iron-restricted conditions.^52^ This is very relevant to cultures in potable water during spaceflight, which is where waterborne pathogenic mycobacteria could be found. In fact, *M. avium* 16s rRNA was identified by PCR amplification in three different water condensation samples from the Mir space station.^23^

### Stress response genes

Even though there were LSMMG-linked transcript alterations related to *M. tuberculosis* EHR, the metabolic transcriptome data does not suggest the LSMMG *M. marinum* were under hypoxia. Many of the EHR genes that changed were stress response genes and included *sigH*, chaperone and heat-shock-regulated genes. SigH is one of 18 sigma factors in *M. marinum*^53^ and sigma factors function in promoter sequence recognition; directing initiation of transcription by RNA polymerase.^54^ *sigH* was the only sigma factor gene to increase expression under LSMMG at both the short and long exposure times. Transcript levels of genes regulated directly or indirectly by SigH also changed significantly due to LSMMG exposure. SigH autoregulates its expression by binding to its own promoter and increasing transcription, and SigH is post-translationally regulated by RshA and PknB. RshA binds to SigH and prevents SigH from binding to RNA polymerase and SigH-RshA binding is disrupted by heat or redox stress,^55^ as well as phosphorylation by PknB.^56^ Small/no changes were detected in *rshA* and *pknB* transcript levels in LSMMG *M. marinum*, but conditions inside the LSMMG mycobacteria could have induced a substantial activity change in SigH by disrupting binding to RshA. A 1.5-fold increase in the *sigH* transcript is implicated in the 40-fold increase in *hsp* transcript after long exposure to LSMMG.

SigH is also required for the induction of *sigB* and *sigE*,^54^ and transcript levels from these genes increased after exposure to LSMMG for 4 days and ~35 h albeit by a small amount (Figure 4). SigE has been implicated in the repression of *icl,*^57^ so increased SigE expression after the long exposure time to LSMMG may explain the decrease in the *icl* transcript.

SigH is known to be involved in the response of mycobacteria to oxidative stress as well as heat shock. Therefore *M. marinum* exposed to LSMMG would be expected to be resistant to H_2_O_2_. AlgU is another alternate sigma factor involved in heat shock and oxidative stress that has been implicated in the LSMMG response of *P. aeruginosa*^11^ and LSMMG-exposed *P. aeruginosa* were more resistant to H_2_O_2_ treatment.^11^ However, LSMMG-exposed LHM4 were more sensitive to H_2_O_2_ in our study. LHM4 was derived from 1218R by integration of an RFP-expressing plasmid (pDEAM2) at the *attB* site in the genome. The *attB* site is used to integrate plasmids into mycobacterium, as integration does not disrupt any genes.^58^ Previous studies have demonstrated that a *M. marinum* strain similar to LHM4 that was derived by pDEAM2 integration into 1218R had similar growth characteristics and virulence in Medaka fish to 1218R.^59^ We also determined that LHM4 and 1218R had similar growth characteristics under LSMMG. Therefore, LSMMG-exposed 1218R would be expected to be sensitive to H_2_O_2_. Interestingly, *S. aureus*, *S. typhimurium*, *E. coli*, *Enterobacter cloacae*, and *Citrobacter freundii* grown under LSMMG have also been found to be more sensitive to H_2_O_2_ than NG controls.^12,60^ A second group using *S. typhimurium* did find that LSMMG induced resistance to H_2_O_2_ and that resistance involved induction of catalase.^30^ Sensitivity to H_2_O_2_ could be altered by the availability of detoxification enzymes (catalase and alkyl hydroperoxidases), proteins involved in ‘repairing’ oxidized proteins (thioredoxins and methionine sulfoxide reductases) or redox buffers such as mycothiol and ergothioneine.^51^ Transcript levels of the genes encoding these proteins or the enzymes involved in the synthesis of mycothiol and ergothioneine did not significantly decrease in LSMMG *M. marinum*, which suggests these protection mechanisms were not compromised compared with the NG control. In fact, transcriptome data indicate that the *trxB1* transcript, which encodes one of the thioredoxins, increased by 4-fold after the long exposure time. Cysteine, which is key to many of the protecting enzymes/compounds, was also being conserved and production increased: the transcript of cysteine desulfurase decreased by 2-fold after LSMMG exposure and the *cysK2* transcript increased ~4-fold after the long exposure to LSMMG. Even though LSMMG *M. marinum* were more sensitive to H_2_O_2_, they had no change in sensitivity to killing by ionizing radiation compared with the NG control. Ionizing radiation and H_2_O_2_ are similar in that they both induce oxidative stress and generate similar types of DNA damage^61^ and DNA is a critical target in cells for cell killing. Lack of enhanced killing by radiation suggests the increased sensitivity to H_2_O_2_ was due to a different mechanism of radical formation/ cell killing by H_2_O_2_ in LSMMG bacteria that could not be defended against by the SigH response. In mycobacteria, the outer cell wall is the first line of defense against H_2_O_2_. This is not the case for ionizing radiation. Ionizing radiation deposits energy in the molecules of the cell and generates DNA damage by depositing energy directly on the DNA or by depositing energy in water, which produces hydroxyl radicals that then induce DNA damage.^62^ The energy deposited by the radiation can also generate lipid peroxides and oxidation of protein molecules, but the delivery of the energy to the macromolecules is not impeded by the structures surrounding or within the bacterium. Therefore, ionizing radiation energy deposition and DNA damage production will occur even if the cell wall composition changes. H_2_O_2_ can pass through lipid bilayers but is broken down to hydroxyl radicals by the Fenton reaction, which requires Fe^2+^ (ref. 63). The hydroxyl radicals can generate lipid peroxides from reaction with lipids in the cell wall that can diffuse and generate DNA damage.^64^ The composition of the cell wall and the iron content of the cell could therefore modify radical formation and alter the sensitivity of the cell to H_2_O_2_. There were a substantial number of transcript changes in LSMMG *M. marinum* related to the cell wall. Transcripts of genes related to MA and phenolic glycolipid synthesis were decreased, while transcripts encoding proteins involved in modifying the types of MA were increased. LSMMG-exposed *M. marinum* also had an increase in transcripts related to mycobactin synthesis and mycobactins bind iron from the environment and transport the charged iron across the hydrophobic cell wall.^39^ An increase in mycobactin synthesis could therefore increase iron content in the mycobacterium. Alterations to mycobactin synthesis and to the composition of the mycobacterium cell wall could therefore explain why the LSMMG-exposed mycobacterium, even with the *sigH* operon response, were more sensitive to H_2_O_2_ but not ionizing radiation.

The changes at the cell wall did not sensitize the LSMMG *M. marinum* to pH 3.5 and so cell wall lipid composition may not influence acid resistance. Other bacteria exposed to LSMMG have also been found not to have increased sensitivity to killing by acid^60^ even though they were more sensitive to H_2_O_2_. Recently, *M. tuberculosis* acid resistance was linked to asparagine metabolism.^65^ An asparagine transporter AnsP2 imports asparagine into the bacterium where asparaginase AnsA breaks down asparagine to aspartate with the production of ammonia. The ammonia buffers hydrogen ions by forming NH_4_^+^. AnsA is also secreted to perform this reaction in the environment around the mycobacterium. In *M. marinum*, AnsP2 is encoded by *MMAR_0627* and AnsA is encoded by *MMAR_2360*. Transcript levels of these genes did not alter during exposure to LSMMG and hence the ability to produce ammonia may not have changed in LSMMG-exposed *M. marinum*. This may explain why LSMMG-exposed *M. marinum* were not more sensitive to killing by acid.

### Could LSMMG alter the virulence of *M. marinum*?

*sigB*, *E* and *H* as well as *hsp* and *dnaK* are induced when *M. marinum* infect fish or are ingested by mosquito larvae,^53^ and *M. marinum* ingested by mosquito larvae are more virulent in fish compared with *in vitro* cultured *M. marinum.*^59^ Exposure to the mosquito larvae gut therefore primes the *M. marinum* for survival inside fish macrophage. The *hsp* gene is also the most induced *M. tuberculosis* gene during macrophage infection,^52^ and *sigH* mutants of *M. tuberculosis* and *M. avium* subsp. *paratuberculosis* have reduced survival in animal infection models.^66,67^ Induction of the SigH stress response by LSMMG before infection should aid survival of mycobacteria in macrophage and potentially increase virulence. The transcriptome of LSMMG *M. marinum* is also similar to mycobacteria inside macrophage in that they have altered growth and metabolism to survive under a nutrient- and iron-starved environment.^39,43,52^ LSMMG increased transcripts involved in lipid degradation and mycobacteria inside macrophage use host lipids for a source of energy.^37^ However, Icl expression would need to increase; studies have shown that Icl is essential to connect lipid degradation with the glyoxylate shunt, and is required for survival in animals.^35^ Even though LSMMG *M. marinum* do have a number of adaptations that would prime them for increased virulence, the enhanced killing by H_2_O_2_ is likely to be detrimental for bacterial survival inside macrophage. Future studies examining LSMMG- or spaceflight-exposed *M. marinum* survival inside macrophage and fish are needed to determine whether *M. marinum* in space could pose a risk to the health of astronauts. As *M. marinum* is an accepted model of waterborne mycobacteria and *M. tuberculosis*, these results do have implications for other pathogenic mycobacteria exposed to LSMMG as they may respond with similar transcriptome changes.

## Materials and methods

### Bacterial strains and culture

*M. marinum* strain 1218R,^53^ a strain isolated from infected fish, and a derivative of this strain, LHM4, were used for this study. LHM4 carries an integrated plasmid pDEAM2, which encodes red fluorescent protein and confers kanamycin resistance. Bacteria were grown in the dark at 30 °C in tubes in a rotating incubator (120 r.p.m.)^27^ or in HARVs attached to a rotating cell culture system (RCCS, Synthecon, Houston, TX, USA) in Middlebrook 7H9 broth (Difco, Detroit, MI, USA) supplemented with ADS (5% BSA Fraction V, 2% Dextrose, 0.81% NaCl) to 10%, 0.5% glycerol, 100 μg cycloheximide/ml, and 0.2% Tween 80. Cultures were also grown on solid Middlebrook 7H10 agar (Difco) supplemented with 10% ADS, 0.5% glycerol, and 100 μg cycloheximide/ml. LHM4 cultures also contained 20 μg kanamycin/ml. To initiate cultures, solid medium was inoculated from a frozen stock and incubated for ~7 days to obtain colonies. Colonies were used to inoculate tube cultures (~3 ml). The tube cultures were grown for 5–7 days to an OD_600_ ~1. This tube culture was then diluted to OD_600_ 0.0005 or 0.002 before growth in 10 or 50 ml HARVs, respectively. The lower OD_600_ of the culture at the start of growth in the 10 ml HARVs prevented the cultures from being at saturation density for the majority of the 4-day exposure time. For the short exposure time, the cultures were grown in 50 ml HARVs. For the long exposure, the cultures were grown in 10 ml HARVs for 4 days and then re-diluted to OD_600_ 0.002 in 50 ml HARVs for another ~35 h. For NG, HARVs were rotated (25 r.p.m.) on an axis parallel to the direction of gravitational force, while they were rotated (25 r.p.m.) on an axis perpendicular to the direction of the gravitational force for LSMMG.^3^

### Growth studies

For the continuous growth studies, one large volume of OD_600_ 0.0005 culture was prepared from an initiating tube culture and split between two 10 ml HARVs: one was subjected to NG and the other to LSMMG. After 4 days the cultures were removed and the OD_600_ was determined. The statistical difference between the LSMMG and NG cultures was tested by a *T*-test performed using SAS 9.4 (SAS Gary, NC, USA). To examine culture density during exponential growth, one large volume of OD_600_ 0.002 culture was prepared from an initiating tube culture and split between two 50 ml HARVs: one was subjected to NG and the other to LSMMG. Where specified, 3 μg catalase/ml (0.06 U/ml; ThermoFisher Scientific, Waltham, MA, USA) was included. Bacteria were grown for ~150 h but the HARVs were stopped to remove samples (1–2 ml). Liquid medium was added to the HARV to replace the removed sample volume (2–4% of the volume) to maintain a low-shear environment. In order to obtain accurate OD_600_ and CFU measurements, a disaggregation procedure was used: SDS (final concentration 0.01%) was added; the culture was passed through a 27.5 gauge needle and either vortexed before measurement of the OD_600_ or preparation of dilutions, or the bacteria were allowed to settle for 5 min before growth on solid medium. Use of the syringe needle, vortexing, and then allowing the culture to settle before plating on solid medium is standard procedure for mycobacterium.^32^ Colonies from each sample were grown on at least two plates of solid medium for 7–10 days. Colonies were counted for each time point and the CFU/ml calculated. Preliminary experiments confirmed by microscopy that disaggregation of cultures was achieved using the syringe needle, and initial experiments determined that the addition of the SDS to the NG and LSMMG cultures only during the disaggregation and dilution stage did not alter colony forming ability (data not shown).

To determine statistical differences between LSMMG and NG cultures during the 37–70 h time period of growth, regression analysis was performed on the OD_600_ data using SAS 9.4 (SAS).

### Size of bacterium

A portion of the LHM4 culture grown for 4 days in a 10 ml HARV was passed through a 27.5 gauge needle and fixed by the addition of 30 μl 4% paraformaldehyde to 10 μl of culture. Samples were placed at 4 °C for at least 24 h before the measurement of the diameter and length of 50 bacteria using microscopy. The Instat3 program and the Unpaired *T*-test or the Mann–Whitney test was used to compare the lengths and diameters of the bacterium.

### RNA preparation

RNA extraction from *M. marinum* strain 1218R was performed according to Rustad *et al.*^68^ RNA integrity (Supplementary Table 1) was assessed on an Agilent 2100 Bioanalyzer (Agilent, Santa Clara, CA, USA) and quantitated using the Qubit RNA BR assay (Invitrogen, ThermoFisher, Waltham, MA, USA).

### RNA-Seq analyses

RNA samples were subjected to DNase treatment, purification with the RNeasy MinElute Cleanup kit (Qiagen, Valencia, CA, USA) and removal of the rRNA and preparation of the RNA-Seq libraries using the ScriptSeq Complete kit for bacteria (Epicentre Biotechnologies, Madison, WI, USA). The DNA libraries (4 nmol/l) were pooled, denatured with 0.2 mol/l sodium hydroxide, and diluted to 12 pmol/l. A 76-cycle paired-end read sequencing run was performed on an Illumina Miseq desktop sequencer (Illumina, Inc., San Diego, USA). The number of sequence reads is shown in Supplementary Table 1.

Seventy-five base-paired-end RNA-Seq reads were aligned to the *M. marinum* strain M genome (Genbank accession number CP000854) using the Spliced Transcripts Alignment to a Reference (STAR) aligner. Reads were visualized using the Integrative Genomics Viewer (IGV, Broad Institute, http://www.broadinstitute.org/igv/home). Reads were aligned to the *M. marinum* strain M transcriptome using RSEM version 1.2.9 for transcript quantification. The fragments per kilobase of transcript per million mapped reads (FPKM) was calculated for each gene and sample gene level differential expression analysis was performed using EBseq version 1.1.5 within RSEM. For the short or long exposure times, the LSMMG samples were compared with NG samples. The LSMMG samples from the two exposure times (short and long) were also compared. Changes in expression were significant if *P*<0.05. The functions of genes were assigned according to the Marinolist (http://mycobrowser.epfl.ch/marinolist.html). Pathway assignment used the Marinolist advanced search and the literature, and *M. marinum* orthologs of *M. tuberculosis* genes were identified using http://tuberculist.epfl.ch. Transcript levels for the genes assigned to pathways were analyzed and heat maps generated using GeneSifter (http://www.geospiza.com/Products/AnalysisEdition.shtml). RNA-Seq data has been deposited in the GeneLab database.

### Reverse-transcriptase quantitative PCR

RNA samples were treated with DNase and converted to cDNA using Moloney Murine Leukemia Virus reverse transcriptase. qPCR reactions in duplicate were performed with primers (Supplementary Table 8) and Power SYBR Green PCR Master Mix (Applied Biosystems, Foster City, CA, USA) using a 7500 Fast Real-Time PCR system (Applied Biosystems) according to the manufacturer’s recommendations. The cDNA level for transcripts was normalized to the cDNA level for 16s rRNA for each sample. This ratio was averaged for the three independent samples and the fold change in expression was calculated by dividing the LSMMG average by the NG average.

### Survival studies

LHM4 were removed from the HARV and the OD_600_ measured in a sample after disaggregation. Bacteria were in exponential phase (OD_600_ 0.09–0.2). For radiation and hydrogen peroxide (H_2_O_2_) studies, bacteria were centrifuged and washed in phosphate-buffered saline containing 0.2% Tween 80 (Tw-PBS), before resuspension at OD_600_ ~0.2 in Tw-PBS. Two samples (200 μl in a sealed tube) were prepared for each time point or dose. One sample was treated (1–4 h) with 5 mmol/l H_2_O_2_ at 30 °C or with gamma radiation (100–400 Gy) at room temperature. The second sample was not treated and was used as the control to determine the CFU/ml at zero treatment at the time point. A similar method was used to treat the bacteria with acid. The bacteria were centrifuged and resuspended at OD_600_ ~0.2 in growth medium (control) or growth medium at pH 3.5. A small amount of one mole per liter citric acid was added to the medium to reach pH 3.5. Once the bacteria were resuspended, the pH was checked using pH paper. The control and acid-treated samples were incubated at 30 °C for 1–4 h. After all treatment regimens, bacteria were disaggregated and diluted as described above and grown on three plates containing solid medium. Bacteria were incubated at 30 °C for 7–10 days and the CFU/ml calculated for each control and treated sample. Survival was calculated by:

$$\%\;\mathrm{Survival}=\frac{\mathrm{Avg}\;\mathrm{CFU}/\mathrm{ml}\;\mathrm{of}\;\mathrm{treated}\;\mathrm{culture}}{\mathrm{Avg}\;\mathrm{CFU}/\mathrm{ml}\;\mathrm{of}\;\mathrm{control}\;\mathrm{culture}}\times \;100$$

The survival data were compared by calculating the area under the curve for each data set using the Trapezoidal Rule, and the Student’s *T-*test was used to compare the values. These statistical analyses were performed using SAS 9.4 (SAS).

## Figures and Tables

**Table 1:** *M. marinum* genes that significantly altered expression when grown under LSMMG for both 40 h, and 4 days ~35 h (LSMMG/NG)

*Gene name*	*Function*	*RFC at 40 h*	*RFC at 4 days ~35 h*
*ltp1_1*	Cell wall and cell processes	2.2	1.9
*mmpL3*		0.63	0.67
*MMAR_0493-1*		1.5	1.6
*MMAR_1554-1*		1.7	1.5
*MMAR_2268-1*		0.46	0.50
*cydD*		2.2	1.9
*MMAR_3568-1*		3.4	2.4
*MMAR_3658-1*		8.1	9.1
*lsr2*		0.48	0.57
*fbpA*		0.54	0.63
**			
*MMAR_0119-1*	Conserved hypotheticals	1.9	1.7
*MMAR_0519-1*		0.39	0.35
*MMAR_0853-1*		9.1	9.0
*MMAR_0982-1*		0.46	0.51
*MMAR_2771-1*		1.7	2.2
*MMAR_3007-1*		1.7	1.6
*MMAR_3010-1*		1.8	1.9
*MMAR_3070-1*		2.2	1.8
*MMAR_3088-1*		1.8	1.5
*MMAR_3549-1*		0.24	0.29
*MMAR_4248-1*		3.8	2.2
*MMAR_4306-1*		8.9	4.9
*MMAR_4498-1*		3.5	5.1
*MMAR_4609-1*		2.1	2.4
*MMAR_4647-1*		3.0	1.5
*MMAR_5103-1*		0.39	0.42
*MMAR_5106-1*		0.43	0.58
*MMAR_5437-1*		1.5	1.8
*MMAR_5455-1*		1.8	1.5
**			
*rpsR1*	Information pathway	0.55	0.57
*rplA*		0.58	0.65
*rplJ*		0.48	0.57
*rplC*		0.49	0.55
*rplB*		0.52	0.63
*rplV*		0.46	0.63
*rpmC*		0.61	0.64
*rpsN*		0.67	0.62
*rpsE*		0.51	0.63
*infA*		0.64	0.66
*rpsD*		0.65	0.64
*rpoA*		0.50	0.59
*rplQ*		0.52	0.57
*rplM*		0.61	0.60
*rpsI*		0.63	0.60
*sigH*		1.5	1.5
*nrdE*		1.8	2.2
*hupB*		0.42	0.66
*rpsP*		0.66	0.66
*tsf*		0.61	0.66
*rplY*		0.55	0.64
*rnpA*		0.57	0.58
*celA*	Intermediary metabolism	0.53	0.47
*nirB*	and respiration	0.39	0.48
*hemC*		0.48	0.48
*mrsA*		0.60	0.62
*sdhB*		0.59	0.81
*sdhD*		0.55	0.67
*MMAR_1644-1*		1.8	1.5
*amiC*		0.63	0.64
*csd*		0.49	0.50
*MMAR_2557-1*		1.7	1.9
*MMAR_2844-1*		0.46	0.58
*folE_1*		1.6	1.9
*glnA2*		0.57	0.62
*MMAR_3558-1*	intermediary metabolism and respiration	0.23	0.07
*mobA*		0.33	0.52
*porB*		0.33	0.53
*porA*		0.31	0.46
*putA*		4.0	2.6
*pth*		0.49	0.61
*MMAR_4497-1*		3.3	5.0
*panD*		0.47	0.54
*panC*		0.56	0.58
**			
*desA3_2*	Lipid metabolism	3.9	3.6
*desA3*		2.2	1.8
*fadE23*		1.6	1.9
*pks15/1*		0.53	0.66
*fabG3_1*		2.1	1.7
*fabD*		0.32	0.48
*kasA*		0.28	0.37
*kasB*		0.28	0.37
*accD6*		0.29	0.36
*accA2*		0.63	0.58
*fadB*		0.57	0.59
*pks13*		0.38	0.57
*fadD32*		0.33	0.55
**			
*MMAR_0641-1*	PE/PPE	2.5	2.8
*MMAR_4562-1*		1.9	2.3
*MMAR_4899-1*		2.6	2.0
**			
*hspR*	Regulatory protein	2.6	4.4
*whiB3*		0.16	0.38
*whiB7*		0.65	2.6
*MMAR_2281-1*		0.49	0.60
*MMAR_3703-1*		3.1	2.7
*MMAR_5069-1*		0.64	0.46
**			
*hsp*	Virulence, adaptation	5.7	40.8
*dnaK*	detoxification	3.5	5.38

Only genes that altered significantly by ⩾1.5-fold at both time points are shown. RFC is the ratio of the normalized mean count values for LSMMG over the normalized mean count values for NG.

## Notes

Supplementary Figures

Supplementary Excel Worksheet 1

Supplementary Excel Worksheet 2
