# Supplementary material for: Exposure of Mycobacterium marinum to low-shear modeled microgravity: effect on growth, the transcriptome and survival under stress
Source: NPJ Microgravity. 2016 Dec 1;2:16038–. doi: 10.1038/npjmgrav.2016.38 (PMC5515531; doi:10.1038/npjmgrav.2016.38)
Supplement: Supplementary Tables [file npjmgrav201638-s1.doc]

Table 1 – RNA samples used for RNASeq and RT-qPCR

| Sample  Number | Time in Culture | Type | OD_600_ at time of  RNA preparation | RIN | Number  of Reads |
| --- | --- | --- | --- | --- | --- |
| 1 | 4 days + 35 hrs | NG | 0.13 | 8.0 | 7,967,690 |
| 2 | 4 days + 35 hrs | LSMMG | 0.129 | 8.4 | 6,852,879 |
| 3 | 4 days + 36 hrs | NG | 0.157 | 8.3 | 6,949,365 |
| 4 | 4 days + 36 hrs | LSMMG | 0.134 | 8.0 | 6,694,743 |
| 5 | 39.5 hrs | NG | 0.157 | 9.2 | 6,659,659 |
| 6 | 39.5 hrs | LSMMG | 0.110 | 8.0 | 7,392,246 |
| 7 | 40 hrs | NG | 0.183 | 8.0 | 10,316,973 |
| 8 | 40 hrs | LSMMG | 0.113 | 8.3 | 9,579,977 |
| 9 | 40.5 hrs | NG | 0.179 | 8.4 | 8,618,652 |
| 10 | 40.5 hrs | LSMMG | 0.118 | 8.0 | 7,722,126 |
| 11 | 4 days + 37.5 hrs | NG | 0.307 | 9.2 | 9,586,621 |
| 12 | 4 days + 37.5 hrs | LSMMG | 0.195 | 8.0 | 8,572,128 |

Bacteria were exposed to either normal gravity (NG) or LSMMG for either ~40 hrs (short exposure) or for 4 days prior to re-dilution and exposure for a further ~36 hrs (long exposure). It was necessary to re-dilute the 4 day culture to make sure that the bacteria were all in the same phase of growth when RNA was prepared from the culture. The RNA was DNase-treated and purified through an RNeasy MinElute Cleanup kit prior to determination of the concentration using the Quibit 2.0 RNA BR assay and RNA integrity score (RIN) using the Agilent 2100 Bioanalyzer, RNA 6000 Nano assay. The number of reads from the MiSeq that were used for the RNASeq analysis are also shown.

Table 2 – Number of genes significantly changed in each gene category after short and long exposure times.

| GENE CATEGORY | Number of genes with altered expression (p < 0.05) | | |
| --- | --- | --- | --- |
|  | LSMMG/NG Short | LSMMG/NG Long | LSMMG Long/ LSMMG Short |
| Cell wall and cell processes | 115 | 54 | 74 |
| Conserved hypotheticals | 104 | 64 | 56 |
| Information pathways | 60 | 49 | 14 |
| Insertion seqs and phages | 1 | 0 | 0 |
| Intermediary metabolism and respiration | 162 | 96 | 103 |
| Lipid metabolism | 56 | 36 | 39 |
| PE/PPE | 14 | 8 | 26 |
| Regulatory proteins | 36 | 16 | 20 |
| Unknown | 3 | 0 | 1 |
| Virulence, detoxification, adaptation | 11 | 5 | 14 |

Table 3 - Comparison of gene expression changes detected using RNA-Seq and RT-qPCR

| **Gene Name** | **Function** | **40 hours** | | **4 days ~35 hours** | |
| --- | --- | --- | --- | --- | --- |
|  |  | **RNAseq** | **RT-qPCR** | **RNAseq** | **RT-qPCR** |
| *cysK2* | Cysteine biosynthesis | - | 0.9 | 3.8 | 4.2 |
| *fadD9-1* | Lipid degradation | - | ND | 3.1 | 4.3 |
| *eis* | Enhanced intracellular survival protein | - | 3.5 | 2.5 | 1.8 |
| *hsp* | Heat shock protein | 5.7 | 5.4 | 40.8 | 20.1 |
| *dnak* | chaperone | 3.5 | 2.9 | 5.4 | 5.0 |
| *hspR* | Transcriptional repression of heat shock proteins | 2.6 | 2.7 | 4.4 | 3.3 |
| *mbtA* | Mycobactin synthesis | - | 2.1 | 2.2 | 1.6 |
| *sigH* | Alternative sigma factor, plays a role in stress response | 1.5 | 1.2 | 1.5 | 1.4 |
| *trxB1* | thioredoxin | - | 2.7 | 4.3 | 5.3 |

- = gene not significantly different for LSMMG/NG

ND – not determined

Table 4 – Top 10% of genes with the largest significant alteration in expression for LSMMG versus NG after 40 hrs

| Gene Name | Gene Category | Real Fold Change (LSMMG/NG) |
| --- | --- | --- |
| *MMAR_0853* | Conserved hypotheticals | 9.1 |
| *MMAR_4306* | Conserved hypotheticals | 8.9 |
| *MMAR_3658* | cell wall and cell processes | 8.2 |
| *MMAR_3492* | Conserved hypotheticals | 6.4 |
| *MMAR_4564* | cell wall and cell processes | 6.4 |
| *whiB3* | Regulatory protein | 0.16 |
| *hsp* | virulence, adaptation and detoxification | 5.7 |
| *MMAR_3409* | Intermediary metabolism and respiration | 5.2 |
| *MMAR_2655* | Regulatory protein | 0.21 |
| *MMAR_3558* | Intermediary metabolism and respiration | 0.23 |
| *MMAR_0520* | Regulatory protein | 0.231 |
| *MMAR_1580* | Intermediary metabolism and respiration | 0.24 |
| *MMAR_3410* | Intermediary metabolism and respiration | 4.1 |
| *MMAR_3549* | Conserved hypotheticals | 0.24 |
| *putA* | Intermediary metabolism and respiration | 4.0 |
| *MMAR_4452* | PE/PPE | 0.25 |
| *MMAR_1714* | Intermediary metabolism and respiration | 0.26 |
| *desA3_2* | Lipid metabolism | 3.9 |
| *MMAR_4248* | Conserved hypotheticals | 3.8 |
| *rpmH* | information pathway | 0.27 |
| *kasB* | Lipid metabolism | 0.28 |
| *fas* | Lipid metabolism | 0.28 |
| *MMAR_1725* | Regulatory protein | 0.28 |
| *kasA* | Lipid metabolism | 0.28 |
| *dnaK* | virulence, adaptation and detoxification | 3.5 |
| *MMAR_4498* | Conserved hypotheticals | 3.5 |
| *MMAR_3267* | cell wall and cell processes | 0.29 |
| *accD6* | Lipid metabolism | 0.29 |
| *echA1* | Lipid metabolism | 0.30 |
| *MMAR_3568* | cell wall and cell processes | 3.4 |
| *MMAR_4497* | Intermediary metabolism and respiration | 3.3 |
| *porA* | Intermediary metabolism and respiration | 0.31 |
| *MMAR_1633* | Conserved hypotheticals | 0.31 |
| *MMAR_1581* | cell wall and cell processes | 0.32 |
| *fabD* | Lipid metabolism | 0.32 |
| *MMAR_0335* | Intermediary metabolism and respiration | 3.1 |
| *MMAR_4451* | PE/PPE | 0.32 |
| *MMAR_3703* | Regulatory protein | 3.10 |
| *porB* | Intermediary metabolism and respiration | 0.33 |
| *mobA* | Intermediary metabolism and respiration | 0.33 |
| *fadD32* | Lipid metabolism | 0.33 |
| *MMAR_4647* | Conserved hypotheticals | 3.01 |
| *MMAR_4247* | Conserved hypotheticals | 2.9 |
| *MMAR_3489* | Intermediary metabolism and respiration | 2.9 |
| *MMAR_0691* | Conserved hypotheticals | 0.36 |
| *MMAR_4713* | Intermediary metabolism and respiration | 0.36 |
| *MMAR_2354* | Conserved hypotheticals | 0.37 |
| *grpE* | virulence, adaptation and detoxification | 2.7 |
| *pks13* | Lipid metabolism | 0.38 |
| *nirB* | Intermediary metabolism and respiration | 0.38 |
| *MMAR_0519* | Conserved hypotheticals | 0.39 |
| *hspR* | Regulatory protein | 2.6 |
| *ltp2* | Lipid metabolism | 2.6 |
| *MMAR_0799* | Regulatory protein | 0.39 |
| *dnaJ* | virulence, adaptation and detoxification | 2.6 |
| *MMAR_5103* | Conserved hypotheticals | 0.39 |

Table 5 – Top 10% of genes with the largest significant alteration in expression for LSMMG versus NG after 4 days ~35 hrs

| Gene Name | Gene Category | Real Fold Change (LSMMG/NG) |
| --- | --- | --- |
| *hsp* | virulence, adaptation and detoxification | 40.8 |
| *MMAR_3556* | intermediary metabolism, and respiration | 0.063 |
| *MMAR_3558* | intermediary metabolism, and respiration | 0.065 |
| *MMAR_3555* | intermediary metabolism, and respiration | 0.088 |
| *icl* | intermediary metabolism, and respiration | 0.098 |
| *MMAR_0218* | cell wall and cell processes | 9.5 |
| *MMAR_3554* | cell wall and cell processes | 0.11 |
| *MMAR_0232* | conserved hypotheticals | 9.2 |
| *MMAR_3658* | cell wall and cell processes | 9.1 |
| *MMAR_0853* | conserved hypotheticals | 9.0 |
| *MMAR_0524* | conserved hypotheticals | 7.8 |
| *mmpS5* | cell wall and cell processes | 0.14 |
| *dnaK* | virulence, adaptation and detoxification | 5.4 |
| *MMAR_1007* | conserved hypotheticals | 0.19 |
| *MMAR_4498* | conserved hypotheticals | 5.1 |
| *MMAR_4497* | intermediary metabolism, and respiration | 5.0 |
| *lat* | intermediary metabolism, and respiration | 5.0 |
| *MMAR_4306* | conserved hypotheticals | 4.9 |
| *MMAR_0523* | conserved hypotheticals | 4.7 |
| *mmpL5* | cell wall and cell processes | 0.22 |
| *hspR* | regulatory proteins | 4.4 |
| *trxB1* | intermediary metabolism, and respiration | 4.3 |
| *cysK2* | intermediary metabolism, and respiration | 3.8 |
| *desA3_2* | lipid metabolism | 3.6 |
| *MMAR_4903* | conserved hypotheticals | 3.6 |
| *MMAR_3549* | conserved hypotheticals | 0.29 |
| *MMAR_1754* | cell wall and cell processes | 3.4 |
| *MMAR_1241* | conserved hypotheticals | 3.2 |
| *fadD9_1* | lipid metabolism | 3.1 |
| *MMAR_3364* | cell wall and cell processes | 3.0 |
| *metC* | intermediary metabolism, and respiration | 3.0 |
| *MMAR_2772* | cell wall and cell processes | 2.9 |
| *ethR* | regulatory proteins | 0.35 |

Table 6 – Overlap of genes/ categories of genes with altered expression compared to NG controls for *M. marinum* grown under LSMMG and *P. aeruginosa* grown in spaceflight.

| **Protein encoded by gene(s)** | ***P. aeruginosa* grown in space-flight** | | ***M. marinum* short exposure** | | ***M. marinum* long exposure** | |
| --- | --- | --- | --- | --- | --- | --- |
|  | **Gene** | **Gene expression change** | **Gene** | **Gene expression change** | **Gene** | **Gene expression change** |
| Ribosomal binding proteins | *rpsA, rpsB, rpsC, rpsD, rpsE, rpsF, rpsG, rpsH, rpsI, rpsK, rpsL, rpsM, rpsP, rpsQ, rpsS, rpsT, rpsU, rplB, rplC, rplD, rplS, rplF, rplL, rplJ, rplK, rplM, rplR, rplU, rplV, rplW, rplX, rpmB, rpmD, rpmE, rpmH, rpmF, rpmJ* | ↓ | *rplA, rplB, rplC, rplJ, rplM, rplO, rplQ, rplT, rplV, rplY, rpmB2_1, rpmC, rpmD, rpmG2, rpmH, rpmL, rpsA, rpsD, rpsE, rpsI, rpsN, rpsO, rpsP, rpsR1* | ↓ | *rplA, rplB, rplC, rplD, rplJ, rplM, rplN, rplO, rplQ, rplV, rplY, rpmC, rpmD, rpmJ, rpsA, rpsD, rpsE, rpsF, rpsG, rpsI, rpsN, rpsP, rpsR1, rpsT* | ↓ |
| RNA polymerase subunit A | *rpoA* | ↓ | *rpoA* | ↓ | *rpoA* | ↓ |
| 16s rRNA-processing protein | *rimM* | ↓ | *rimM* | NS | *rimM* | ↓ |
| Nitrite reductase | *nirS* | ↑ | *nirB, nirD* | ↓ | *nirB, nirD* | ↓ |
| Succinate dehydrogenase | *sdhB, sdhC, sdhD* | ↓ | *sdhB, sdhC, sdhD* | ↓ | *sdhB, sdhD* | ↓ |
| Isocitrate lyase | *PA2634* | ↓ | *icl* | NS | *icl* | ↓ |
| Pyruvate dehydrogenase E1 component | *PA3416, PA3417* | ↑ | *MMAR_3489* | ↑ | *MMAR_3489* | NS |
| Elongation factor Tsf | *tsf* | ↓ | *tsf* | ↓ | *tsf* | ↓ |
| Adenylate kinase | *adk* | ↓ | *adk* | ↓ | *adk* | NS |
| tRNA [guanine-*N*(1)-]-methyltransferase | *trmD* | ↓ | *trmD* | NS | *trmD* | ↓ |
| Cysteine desulfurase | *iscS* | ↓ | *csd, iscS* | ↓ | *csd* | ↓ |
| Ribonucleotide diphosphate reductase | *nrdA* | ↓ | *nrdE* | ↑ | *nrdE, nrdH, nrdF2* | ↑ |
| Translation initiation factor  IF-1 | *infA* | ↓ | *infA* | ↓ | *infA* | ↓ |
| Elongation factor P | *efp* | ↓ | *efp* | ↓ | *efp* | NS |

The list of genes with significant changes in expression at the short and long exposure time points for *M. marinum* were compared to those found to change ≥ 2 fold in *P. aeruginosa* grown in space-flight^13^. Where possible the same genes were identified in *M. marinum* and *P.aeruginosa*, but types of genes were also considered. The genes shown here significantly changed in *M. marinum* grown under LSMMG compared to NG and in *P. aeruginosa* grown in space-flight compared to ground controls. If a gene was significantly changed at one of the time points for *M. marinum* but not at the other time point, the gene is still shown. NS = not significant.

Table 7 – Overlap of genes/ categories of genes with altered expression compared to NG controls for *M. marinum* and *P. aeruginosa* grown under LSMMG.

| **Protein encoded by gene(s)** | ***P. aeruginosa* grown under LSMMG** | | ***M. marinum* short exposure** | | ***M. marinum* long exposure** | |
| --- | --- | --- | --- | --- | --- | --- |
|  | **Gene** | **Gene expression change** | **Gene** | **Gene expression change** | **Gene** | **Gene expression change** |
| Ribosomal binding proteins | *rplO, rpmD, rpsE, rpsH, rplF, rplN, rplW, rplC, rplA, rplM, PA4671, rpmB, rpmH* | ↑ | *rplA, rplB, rplC, rplJ, rplM, rplO, rplQ, rplT, rplV, rplY, rpmB2_1, rpmC, rpmD, rpmG2, rpmH, rpmL, rpsA, rpsD, rpsE, rpsI, rpsN, rpsO, rpsP, rpsR1* | ↓ | *rplA, rplB, rplC, rplD, rplJ, rplM, rplN, rplO, rplQ, rplV, rplY, rpmC, rpmD, rpmJ, rpsA, rpsD, rpsE, rpsF, rpsG, rpsI, rpsN, rpsP, rpsR1, rpsT* | ↓ |
| RNA polymerase subunit A | *rpoA* | ↑ | *rpoA* | ↓ | *rpoA* | ↓ |
| Succinate dehydrogenase | *sdhA, sdhB, sdhD* | ↑ | *sdhB, sdhC, sdhD* | ↓ | *sdhB, sdhD* | ↓ |
| Isocitrate lyase | *PA2634* | ↑ | *icl* | NS | *icl* | ↓ |
| Elongation factor Tsf | *tsf* | ↑ | *tsf* | ↓ | *tsf* | ↓ |
| Chaperone | *dnaK, GroEL, GroES* | ↑ | *dnaK, dnaJ, dnaJ2* | ↑ | *dnaK* | ↑ |
| Heat shock protein | *grpE, htpG, htpX, ibpA* | ↑ | *grpE, hsp, htpG, hspR* | ↑ | *Hsp, hspR* | ↑ |
| Sigma factors | *rpoH, rpoD, AlgU, sigX* | ↑ | *sigH, sigA* | *sigH* ↑  *sigA* ↓ | *sigH, sigE* | ↑ |
| Phosphate acetyltransferase | *pta* | ↑ | *pta* | ↑ | *pta* | NS |
| Acetate kinase | *PA0836* | ↑ | *ackA* | ↑ | *ackA* | NS |
| Cytochrome c oxidase | *ccoO_1_, ccoO_2_, ccoN_2_,* | ↑ | *ctaD_1* | ↑ | *ctaD_1* | NS |
| Aconitate hydratase | *acnA* | ↑ | *acn* | ↓ | *acn* | NS |
| Citrate synthase | *gltA* | ↑ | *gltA2* | ↑ | *gltA2* | NS |
| Succinyl-CoA synthetase | *sucD* | ↑ | *sucD* | ↓ | *sucD* | NS |
| Electron transfer flavoprotein | *etfA* | ↑ | *fixB* | ↓ | *fixB* | NS |
| Isocitrate dehydrogenase | *icd* | ↑ | *icd1, icd2* | ↑ | *icd1, icd2* | NS |

The list of genes with significant changes in expression at the short and long exposure time points for *M. marinum* were compared to those found to change ≥ 1.5 fold in *P. aeruginosa* grown under LSMMG^11^. Where possible the same genes were identified in *M. marinum* and *P.aeruginosa*, but types of genes were also considered. The genes shown here significantly changed in *M. marinum* or *P. aeruginosa* grown under LSMMG compared to NG. If a gene was significantly changed at one of the time points for *M. marinum* but not at the other time point, the gene is still shown. NS = not significant.

Table 8 – Primers used for RT-qPCR

| CysK2-2F | 5’ ATCCCGACCAATACAGCAA 3’ |
| --- | --- |
| CysK2-2R | 5’ CGATGAATGTCCACCAGTTC 3’ |
| DNA-KF | 5’ CACCGAACTGGTCAAGGA 3’ |
| DNA-KR | 5’ CGTCTTTCACCTCACCCT 3’ |
| FadD91-1F | 5’ CGGTATCGCAATCAGCAAG 3’ |
| FadD91-1R | 5’ TTTGAATGATCTGTGGTAATCCC 3’ |
| Eis-1F2 | 5’ gcagtctctacctgggtg 3’ |
| Eis-1R2 | 5’ gcaatcacgccaacatctt 3’ |
| Hsp-1F2 | 5’ CGTCTCGGCTTCCTATGA 3’ |
| Hsp-1R2 | 5’ ACTTGGTGATTGCGATGC 3’ |
| HspRF | 5’ TATGACAGGCTTGGTCTGG 3’ |
| HspRR | 5’ CTGACGCAGCAGATTGAC 3’ |
| MbtAF | 5’ CGCCTTGTTACTCGTCTCA 3’ |
| MbtAR | 5’ CGAGGTACACGTCGTCAG 3’ |
| SigHF | 5’ GATTCTGACCAATACCTACATC 3’ |
| SigHR | 5’ CTGCCAATCGGTGATCTC 3’ |
| TrxB1F | 5’ ATGGCTACCCAAGACTTAACA 3’ |
| TrxB1R | 5’ CAGCACCATGTCGTTGTC 3’ |
| 16srRNAF | 5’ GGAGCGAACAGGATTAGATAC 3’ |
| 16srRNAR | 5’ CCAAGGAAGGAAACCCAC 3’ |
