# Supplementary material for: Exposure of Mycobacterium marinum to low-shear modeled microgravity: effect on growth, the transcriptome and survival under stress
Source: NPJ Microgravity. 2016 Dec 1;2:16038–. doi: 10.1038/npjmgrav.2016.38 (PMC5515531; doi:10.1038/npjmgrav.2016.38)
Supplement: Supplementary Figures [file npjmgrav201638-s2.ppt]

## Slide 1
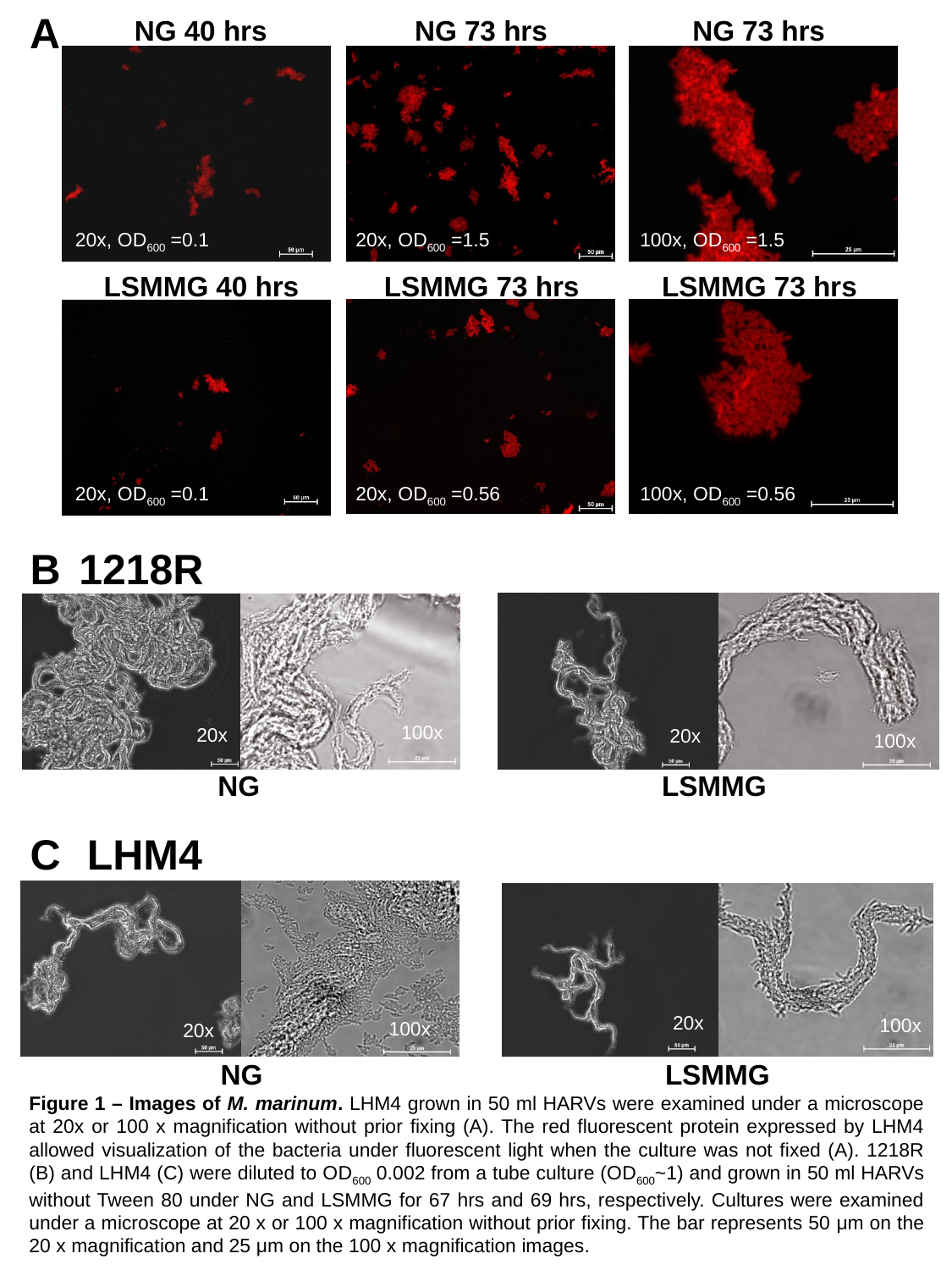

A
NG 40 hrs
NG 73 hrs
NG 73 hrs
20x, OD600 =0.1
20x, OD600 =1.5
100x, OD600 =1.5
LSMMG 40 hrs
LSMMG 73 hrs
LSMMG 73 hrs
20x, OD600 =0.1
20x, OD600 =0.56
100x, OD600 =0.56
B
1218R
20x
100x
100x
20x
NG
LSMMG
C
LHM4
20x
100x
20x
100x
NG
LSMMG
Figure 1 – Images of M. marinum. LHM4 grown in 50 ml HARVs were examined under a microscope at 20x or 100 x magnification without prior fixing (A). The red fluorescent protein expressed by LHM4 allowed visualization of the bacteria under fluorescent light when the culture was not fixed (A). 1218R (B) and LHM4 (C) were diluted to OD600 0.002 from a tube culture (OD600~1) and grown in 50 ml HARVs without Tween 80 under NG and LSMMG for 67 hrs and 69 hrs, respectively. Cultures were examined under a microscope at 20 x or 100 x magnification without prior fixing. The bar represents 50 μm on the 20 x magnification and 25 μm on the 100 x magnification images.

## Slide 2
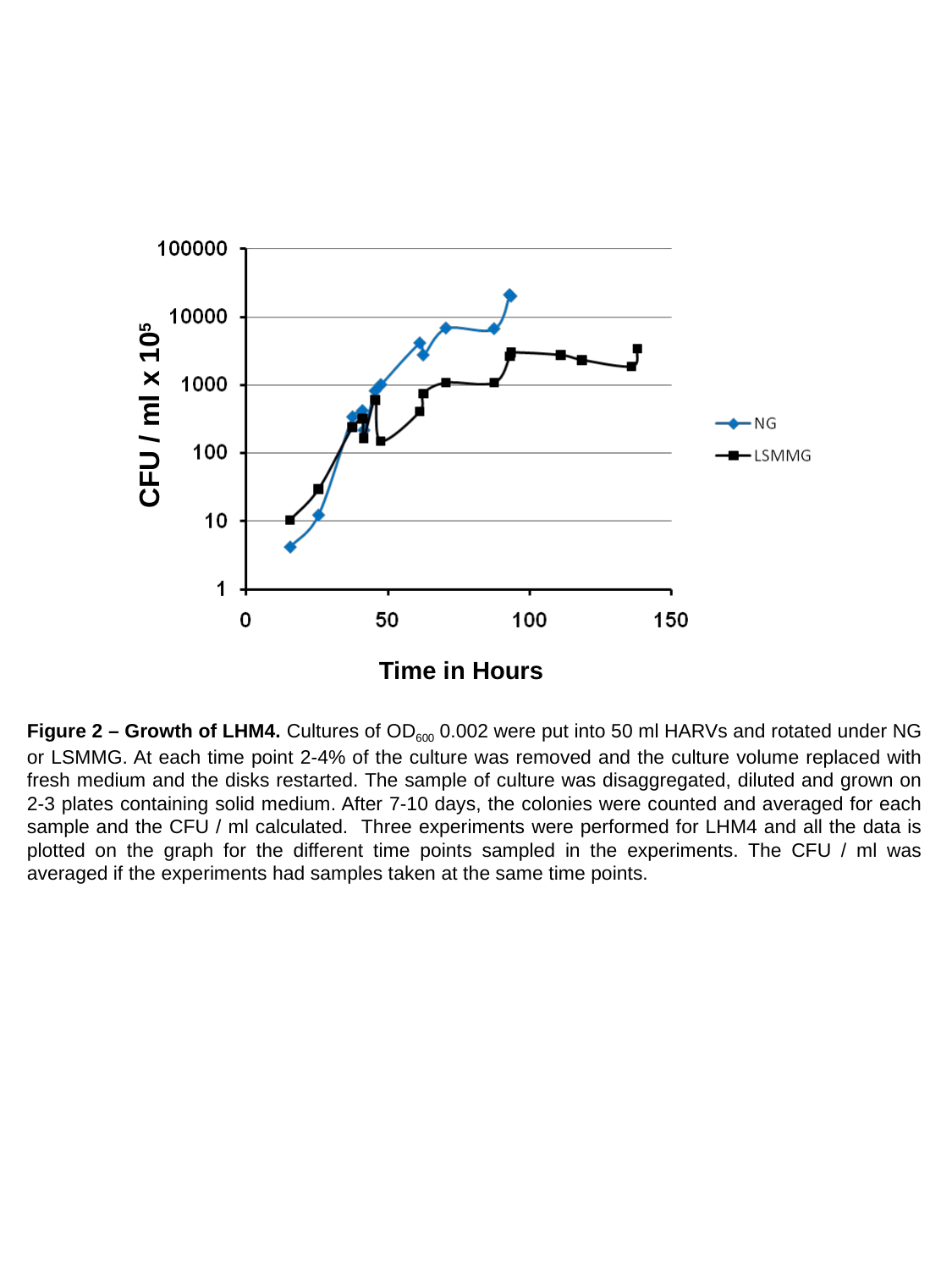

CFU / ml x 105
Time in Hours
Figure 2 – Growth of LHM4. Cultures of OD600 0.002 were put into 50 ml HARVs and rotated under NG or LSMMG. At each time point 2-4% of the culture was removed and the culture volume replaced with fresh medium and the disks restarted. The sample of culture was disaggregated, diluted and grown on 2-3 plates containing solid medium. After 7-10 days, the colonies were counted and averaged for each sample and the CFU / ml calculated. Three experiments were performed for LHM4 and all the data is plotted on the graph for the different time points sampled in the experiments. The CFU / ml was averaged if the experiments had samples taken at the same time points.

## Slide 3
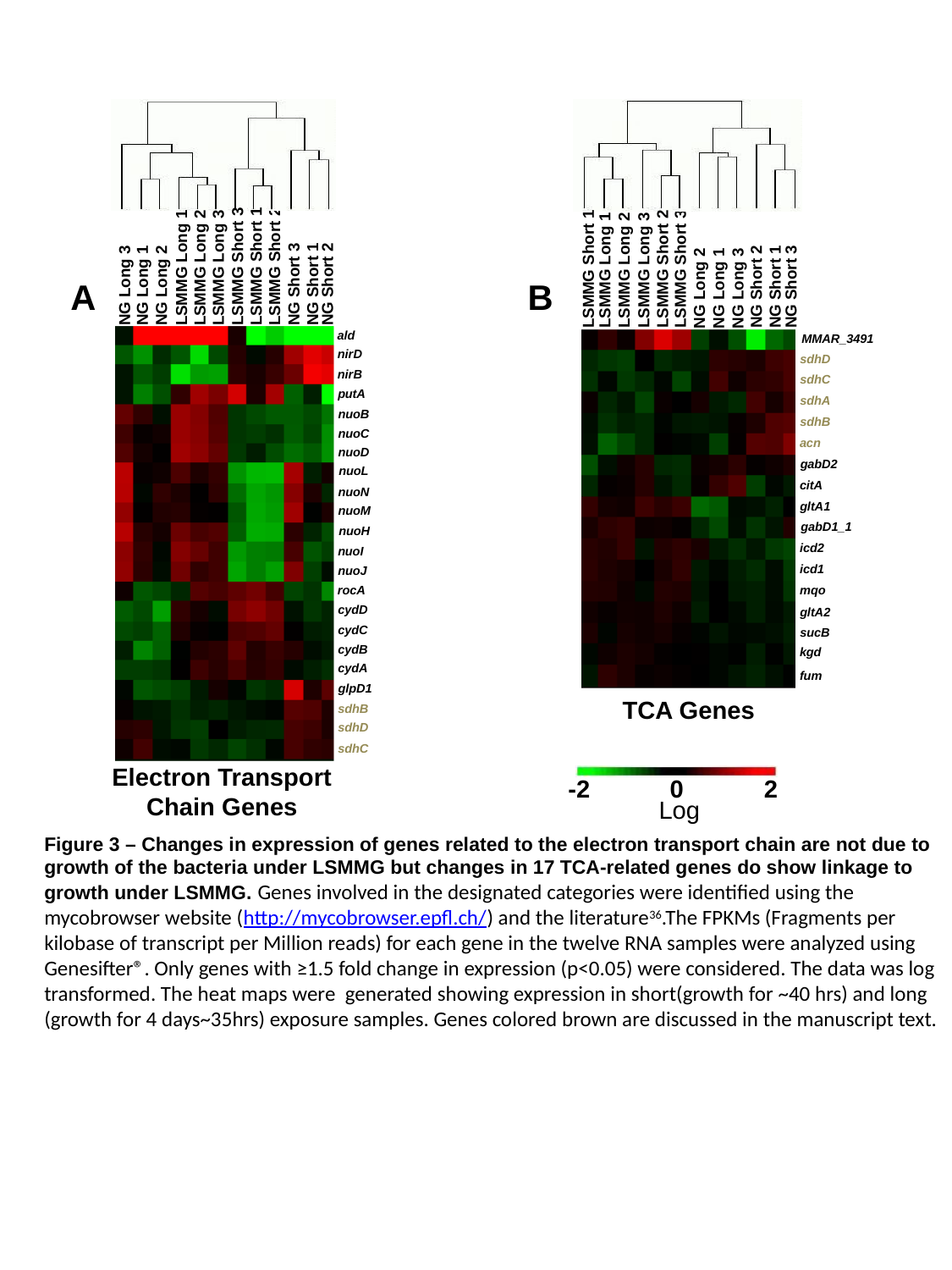

LSMMG Short 3
LSMMG Short 1
LSMMG Short 2
LSMMG Long 1
LSMMG Long 2
LSMMG Long 3
NG Short 3
NG Short 1
NG Short 2
A
NG Long 3
NG Long 1
NG Long 2
ald
nirD
nirB
putA
nuoB
nuoC
nuoD
nuoL
nuoN
nuoM
nuoH
nuoI
nuoJ
rocA
cydD
cydC
cydB
cydA
glpD1
sdhB
sdhD
sdhC
Electron Transport
Chain Genes
LSMMG Short 1
LSMMG Short 2
LSMMG Short 3
LSMMG Long 1
LSMMG Long 2
LSMMG Long 3
B
NG Short 2
NG Short 1
NG Short 3
NG Long 2
NG Long 1
NG Long 3
MMAR_3491
sdhD
sdhC
sdhA
sdhB
acn
gabD2
citA
gltA1
gabD1_1
icd2
icd1
mqo
gltA2
sucB
kgd
fum
TCA Genes
-2
0
2
Log
Figure 3 – Changes in expression of genes related to the electron transport chain are not due to growth of the bacteria under LSMMG but changes in 17 TCA-related genes do show linkage to growth under LSMMG. Genes involved in the designated categories were identified using the mycobrowser website (http://mycobrowser.epfl.ch/) and the literature36.The FPKMs (Fragments per kilobase of transcript per Million reads) for each gene in the twelve RNA samples were analyzed using Genesifter®. Only genes with ≥1.5 fold change in expression (p<0.05) were considered. The data was log transformed. The heat maps were generated showing expression in short(growth for ~40 hrs) and long (growth for 4 days~35hrs) exposure samples. Genes colored brown are discussed in the manuscript text.
