# Supplementary material for: Exposure of Mycobacterium marinum to low-shear modeled microgravity: effect on growth, the transcriptome and survival under stress
Source: NPJ Microgravity. 2016 Dec 1;2:16038–. doi: 10.1038/npjmgrav.2016.38 (PMC5515531; doi:10.1038/npjmgrav.2016.38)
Supplement: Supplementary Excel Worksheet 1 [file npjmgrav201638-s3.pdf]

CELL WALL AND CELL PROCESSES - 115 genes

GENE ID	GENE NAME	FUNCTION	FPKM Fragments Per Kilobase of transcript per Million mapped reads. NORMAL Short			LSMMG Short			PPEE posterior probability that a transcript is equally expressed	PPDE posterior probability that a transcript is differentially expressed	RealFC real fold change is the ratio of the normalized mean count values for LSMMG over the normalized mean count values for normal
			39.5hrs	40hrs	40.5hrs	39.5hrs	40hrs	40.5hrs			
MMAR_0054	MMAR_0054-1	cell wall and cell processes	55.67	64.03	56.32	108.4	89.28	105.24	2.32E-05	0.999976807	1.55892712
MMAR_0055	MMAR_0055-1	cell wall and cell processes	290.61	185.09	207.88	169.37	124.52	146.41	2.95E-05	0.999970517	0.58841262
MMAR_0067	MMAR_0067-1	cell wall and cell processes	251.72	253.17	228.98	303.49	350.2	303.06	0.036829223	0.963170777	1.1821926
MMAR_0142	ltp1_1	cell wall and cell processes	37.41	39.36	44.18	79.37	100.18	109.35	7.26E-10	0.999999999	2.162373095
MMAR_0144	MMAR_0144-1	cell wall and cell processes	245.42	166.99	211.97	332.66	382.04	500.86	0.044910001	0.955089999	1.774246282
MMAR_0255	mmpL5_4	cell wall and cell processes	153.19	144.92	121.52	236.62	220.56	208.41	5.68E-11	1	1.44575683
MMAR_0418	MMAR_0418-1	cell wall and cell processes	332.13	370.93	468.38	226.94	228.85	242.3	0.005347613	0.994652387	0.536818275
MMAR_0419	MMAR_0419-1	cell wall and cell processes	167.15	158.11	225.94	110.14	120.83	108.11	0.006813165	0.993186835	0.556203096
MMAR_0420	MMAR_0420-1	cell wall and cell processes	609.19	628.92	687.68	555.37	550.9	500.47	0.018540355	0.981459645	0.752380124
MMAR_0423	MMAR_0423-1	cell wall and cell processes	141.79	104.1	132.22	99.63	113.88	102.43	0.002000365	0.997999635	0.761497211
MMAR_0444	MMAR_0444-1	cell wall and cell processes	183.94	155.13	134.01	108.44	116.98	102.92	7.05E-08	0.99999993	0.633088328
MMAR_0446	mmpL3	cell wall and cell processes	663.98	548.34	524.83	406.9	409.8	376.94	0	1	0.627237376
MMAR_0461	MMAR_0461-1	cell wall and cell processes	60.81	64.65	47.07	133.64	122.46	128.19	0	1	2.025593581
MMAR_0493	MMAR_0493-1	cell wall and cell processes	64.32	74.32	57.83	96.83	100.57	130.66	0.016600673	0.983399327	1.515329202
MMAR_0502	MMAR_0502-1	cell wall and cell processes	379.69	290.38	179.55	555.66	559.54	460.05	0.008207161	0.991792839	1.699321733
MMAR_0647	MMAR_0647-1	cell wall and cell processes	687.04	583.67	606.88	464.13	523.1	492.07	0	1	0.716425403
MMAR_0671	MMAR_0671-1	cell wall and cell processes	442.42	353.72	396.59	494.08	539.93	541.83	0.000875886	0.999124114	1.202208331
MMAR_0696	lpqK	cell wall and cell processes	257.94	279.68	256.57	339.39	335.16	337.6	0.013675969	0.986324031	1.155663659
MMAR_0705	mmpS1_1	cell wall and cell processes	43.41	62.63	73.43	29.22	23.7	26.76	0.006540251	0.993459749	0.39601038
MMAR_0708	MMAR_0708-1	cell wall and cell processes	57.32	56.14	45.48	104.83	79.41	79.9	0.025144827	0.974855173	1.509987981
MMAR_0770	MMAR_0770-1	cell wall and cell processes	41.97	30.57	42.18	30.68	24.71	26.06	0.000421539	0.999578461	0.647057973
MMAR_0772	mmpS4_2	cell wall and cell processes	656.22	660.15	571.01	462.75	440.95	415.56	2.00E-15	1	0.630670679
MMAR_0804	MMAR_0804-1	cell wall and cell processes	844.09	843.85	755.24	1308.31	1326.06	1149.93	1.14E-11	1	1.406234088
MMAR_0809	lprQ	cell wall and cell processes	507.95	460.26	355.92	341.99	360.06	320.48	0.025909131	0.974090869	0.704198725
MMAR_0890	MMAR_0890-1	cell wall and cell processes	378.67	371.45	272.68	700.26	760.19	581.86	3.90E-12	1	1.789937605
MMAR_0971	secE	cell wall and cell processes	1491.98	1350.2	1216.07	1100.16	984.41	873.4	2.38E-13	1	0.660371473
MMAR_0994	mkl	cell wall and cell processes	1118.34	975.84	1054.13	1440.45	1497.52	1458.48	0	1	1.269313715
MMAR_1187	MMAR_1187-1	cell wall and cell processes	62.92	44.82	38.93	70.46	81.64	83.46	0.04210953	0.95789047	1.470085151
MMAR_1189	MMAR_1189-1	cell wall and cell processes	153.1	115.39	116.7	185.96	187.62	167.32	0.007075925	0.992924075	1.281808581
MMAR_1230	corA_1	cell wall and cell processes	64.4	56.63	48.46	82.51	102.55	99.06	4.25E-06	0.99999575	1.52687805
MMAR_1277	manB	cell wall and cell processes	318.93	320.51	255.47	429.23	391.11	382.85	0.038285669	0.961714331	1.222461965
MMAR_1366	MMAR_1366-1	cell wall and cell processes	137.25	137.89	139.25	110.82	98.94	100.26	0.003003728	0.996996272	0.679134697
MMAR_1371	MMAR_1371-1	cell wall and cell processes	289.33	290.62	262.21	341.61	340.57	336.01	0.006892087	0.993107913	1.099817609
MMAR_1515	MMAR_1515-1	cell wall and cell processes	208.16	167.89	192.78	121.71	113.5	120.04	1.81E-11	1	0.56815717
MMAR_1530	ftsE	cell wall and cell processes	735.9	617.59	586.13	587.35	605.61	549.68	2.23E-05	0.999977728	0.817287583
MMAR_1543	MMAR_1543-1	cell wall and cell processes	249.42	247.31	191.52	447.51	470.78	422.29	0	1	1.767839192
MMAR_1554	MMAR_1554-1	cell wall and cell processes	190	215.88	221.07	394.15	355.31	415.18	2.30E-09	0.999999998	1.681213624
MMAR_1581	MMAR_1581-1	cell wall and cell processes	303.59	284.96	399	125.99	97.8	120.27	7.13E-11	1	0.315936784
MMAR_1696	MMAR_1696-1	cell wall and cell processes	112.21	118.23	93.35	142.37	180.49	201.1	0.042181	0.957819	1.461981667
MMAR_1763	lppX	cell wall and cell processes	805.02	709.09	684.43	627.6	652.61	603.39	0.003194116	0.996805884	0.778272239
MMAR_1787	amt	cell wall and cell processes	835.12	952.18	776.04	587.82	539.35	616.54	1.95E-07	0.999999805	0.617246681
MMAR_1797	dacB	cell wall and cell processes	93.54	69.91	68.79	60.13	66.63	47.75	0.014407181	0.985592819	0.685513001
MMAR_1805	lepB	cell wall and cell processes	307.39	271.44	237.7	201.27	227.39	186.76	1.14E-06	0.999998859	0.68575452
MMAR_1991	MMAR_1991-1	cell wall and cell processes	430.05	336.7	419.22	315.76	323.3	284.13	6.18E-07	0.999999382	0.709245429
MMAR_2014	MMAR_2014-1	cell wall and cell processes	448.56	429.86	416.34	424.2	398.07	343.22	0.009433257	0.990566743	0.815448466
MMAR_2043	MMAR_2043-1	cell wall and cell processes	293.17	232.74	229.1	367.36	395.04	433.09	1.74E-07	0.999999826	1.445086826
MMAR_2106	MMAR_2106-1	cell wall and cell processes	123.76	143.57	161.51	216.65	245.24	216.98	0.016584479	0.983415521	1.417016269
MMAR_2166	MMAR_2166-1	cell wall and cell processes	302	245.17	286.91	237.01	257.5	239.71	3.91E-06	0.999996087	0.800931289
MMAR_2246	secG	cell wall and cell processes	2071.62	1121.67	1761.98	1224.8	1010.27	1297.53	0.036658652	0.963341348	0.649632011
MMAR_2268	MMAR_2268-1	cell wall and cell processes	819.69	792.67	1238.46	480.74	509.69	451.51	0.025964056	0.974035944	0.456043128
MMAR_2388	MMAR_2388-1	cell wall and cell processes	397.29	351.65	371.21	258.56	257.4	252.52	1.23E-08	0.999999988	0.621928213
MMAR_2422	MMAR_2422-1	cell wall and cell processes	65.6	49.46	61.19	137.03	181.77	136.33	6.60E-09	0.999999993	2.351508879
MMAR_2424	cydD	cell wall and cell processes	34.5	37.61	41.51	99.16	84.32	87.51	0	1	2.160681399
MMAR_2426	cydA	cell wall and cell processes	419.77	384.68	464.71	604.95	651.88	722.94	0.000369039	0.999630961	1.416180616
MMAR_2440	MMAR_2440-1	cell wall and cell processes	6332.74	5318.95	7239.49	4705.29	5083.34	4300.41	0.027982253	0.972017747	0.672074676
MMAR_2572	MMAR_2572-1	cell wall and cell processes	84.42	73.98	78.18	48.23	50.6	47.7	0.001971222	0.998028778	0.563305714
MMAR_2647	MMAR_2647-1	cell wall and cell processes	190.14	205.15	160.8	282.24	260.71	241.13	0.005913229	0.994086771	1.280420679
MMAR_2677	MMAR_2677-1	cell wall and cell processes	266.61	219.1	340.19	177.88	189.81	189.06	0.049930713	0.950069287	0.611631542
MMAR_2678	mycP5	cell wall and cell processes	592.3	580.9	611.27	488.81	512.71	471.58	4.86E-10	1	0.749589655
MMAR_2679	MMAR_2679-1	cell wall and cell processes	567.11	451.6	518.92	429.1	433.66	379.56	2.96E-10	1	0.735733445
MMAR_2712	MMAR_2712-1	cell wall and cell processes	260.88	196.8	197.24	175.68	197.45	177.83	0.018143655	0.981856345	0.768764291
MMAR_2772	MMAR_2772-1	cell wall and cell processes	839.6	579.41	510.09	1019.09	1037.57	1055.38	0.000488874	0.999511126	1.474275568
MMAR_2797	nanT	cell wall and cell processes	157.94	138.22	108.09	185.47	190.48	209.51	0.016733073	0.983266927	1.321828019
MMAR_2870	MMAR_2870-1	cell wall and cell processes	143.17	93.04	138.31	84.52	90.95	78.65	9.69E-05	0.999903095	0.618047856
MMAR_2923	MMAR_2923-1	cell wall and cell processes	25.21	22.79	31.81	12.7	20.27	15.86	0.018138405	0.981861595	0.55366841
MMAR_2995	MMAR_2995-1	cell wall and cell processes	163.25	164.56	183.27	231.22	268.86	260.21	0.000228833	0.999771167	1.346455634
MMAR_3011	MMAR_3011-1	cell wall and cell processes	50.33	43.85	33.34	84.17	67.38	68.89	0.001470146	0.998529854	1.578302109
MMAR_3190	ftsZ	cell wall and cell processes	595.48	505.61	551.12	434.09	452.35	437.66	0	1	0.729160975
MMAR_3200	pbpB	cell wall and cell processes	279.63	229.61	255.12	226.41	246.5	212.94	0.000321295	0.999678705	0.81750907
MMAR_3225	MMAR_3225-1	cell wall and cell processes	231.94	218.95	194.02	189.12	199.22	175.24	0.000190236	0.999809764	0.794956448
MMAR_3243	MMAR_3243-1	cell wall and cell processes	2840.79	2782.05	2813.14	3535.47	3948.32	3546.15	0.002249155	0.997750845	1.177544218
MMAR_3263	MMAR_3263-1	cell wall and cell processes	225.64	203.18	253.82	188.03	163.83	177.58	0.000636426	0.999363574	0.703568984
MMAR_3264	MMAR_3264-1	cell wall and cell processes	265.7	291.87	236.47	220.54	169.87	217.29	0.014024659	0.985975341	0.695873977
MMAR_3266	MMAR_3266-1	cell wall and cell processes	117.99	129.45	134.61	70.86	88	72.45	7.39E-09	0.999999993	0.548845854
MMAR_3267	MMAR_3267-1	cell wall and cell processes	268.3	233.59	288.33	56.47	88.99	106.7	0	1	0.288199559
MMAR_3359	MMAR_3359-1	cell wall and cell processes	78.82	58.59	65.16	90.94	105.71	112.1	0.002583705	0.997416295	1.390607942
MMAR_3568	MMAR_3568-1	cell wall and cell processes	28.16	38.11	23.49	113.51	122.06	96.52	0	1	3.345368145
MMAR_3572	MMAR_3572-1	cell wall and cell processes	19.46	28.33	18.55	46.51	36.82	45.62	0.001115246	0.998884754	1.755653816
MMAR_3616	MMAR_3616-1	cell wall and cell processes	124.34	104.08	99.86	175.5	180.41	176.44	2.61E-08	0.999999974	1.478743089
MMAR_3640	MMAR_3640-1	cell wall and cell processes	554.96	620.61	550.23	504.1	447.95	453.04	0.000707842	0.999292158	0.738870872
MMAR_3658	MMAR_3658-1	cell wall and cell processes	91.47	52.9	59.21	512.38	624.41	668.48	0	1	8.137526527
MMAR_3700	MMAR_3700-1	cell wall and cell processes	46.37	75.43	54.18	96.81	93.81	95.13	0.007024656	0.992975344	1.460675734
MMAR_3840	MMAR_3840-1	cell wall and cell processes	719.84	510.05	627.77	528.46	491.97	371.8	4.53E-05	0.999954674	0.677730968
MMAR_4036	MMAR_4036-1	cell wall and cell processes	47.59	48.5	38.77	32.22	37.57	27.09	0.003020909	0.996979091	0.653197343
MMAR_4061	MMAR_4061-1	cell wall and cell processes	345.33	381.9	283.38	515.27	529.14	509.88	1.94E-08	0.999999981	1.395637661
MMAR_4138	oppD	cell wall and cell processes	119.5	106.47	94.23	143.54	170.76	172.54	1.14E-05	0.999988642	1.385768323
MMAR_4139	oppA	cell wall and cell processes	292.64	279.42	301.01	550.68	537.94	538.72	0	1	1.693362677
MMAR_4175	MMAR_4175-1	cell wall and cell processes	72.94	52.95	57.77	136.26	98.21	110.96	0.007026451	0.992973549	1.714876756
MMAR_4203	sugC	cell wall and cell processes	87.3	106.95	71.28	132.62	130.45	120.17	0.033374851	0.966625149	1.308588326
MMAR_4209	MMAR_4209-1	cell wall and cell processes	141.28	125.85	118.28	208.54	194.36	215.47	2.44E-15	1	1.461413424
MMAR_4389	pra	cell wall and cell processes	875.94	702.84	846.92	539.01	609.89	526.31	1.67E-15	1	0.627410687
MMAR_4394	MMAR_4394-1	cell wall and cell processes	782.88	619.47	763.71	521.8	623.68	671.89	0.023169798	0.976830202	0.762958306
MMAR_4437	MMAR_4437-1	cell wall and cell processes	266.19	230.7	248.48	384.14	406.29	400.33	0.000295246	0.999704754	1.450435683
MMAR_4564	MMAR_4564-1	cell wall and cell processes	4.58	4.05	3.18	28.34	18.17	36.12	1.42E-05	0.999985823	6.387382985
MMAR_4576	pstS2	cell wall and cell processes	80.03	61.28	92.9	115.33	138.2	126.42	0.012092751	0.987907249	1.473039429
MMAR_4607	MMAR_4607-1	cell wall and cell processes	128.01	127.73	125.8	234.54	311.03	233.78	2.37E-06	0.999997626	1.84957682
MMAR_4610	MMAR_4610-1	cell wall and cell processes	150.48	142.71	174.88	134.98	119.11	104.87	0.007820771	0.992179229	0.694033715
MMAR_4622	ctpE	cell wall and cell processes	79.95	55.53	61.57	120.31	108.77	120.85	5.77E-10	0.999999999	1.624828873
MMAR_4623	MMAR_4623-1	cell wall and cell processes	424.59	345.73	343.3	615.22	675.82	625.29	0	1	1.569466724
MMAR_4665	rpfA	cell wall and cell processes	251.71	247.52	213.13	206.79	200.28	168.69	8.37E-05	0.999916343	0.734611783
MMAR_4750	MMAR_4750-1	cell wall and cell processes	417.14	358.94	391.69	340.69	321.89	245.98	9.09E-05	0.999909075	0.707953793
MMAR_5087	MMAR_5087-1	cell wall and cell processes	589.11	452.09	568.18	415.57	416.11	406.05	1.60E-09	0.999999998	0.699550603
MMAR_5093	ppm1_2	cell wall and cell processes	170.86	115.86	142.94	120.69	121.63	120.6	0.004227439	0.995772561	0.771263263
MMAR_5095	idsA1	cell wall and cell processes	253.68	243.01	238.25	183.38	225.71	210.56	2.23E-05	0.999977739	0.765396173
MMAR_5101	lsr2	cell wall and cell processes	3110.45	2812.13	3664.4	1934.84	1699.31	1486.41	1.60E-07	0.99999984	0.478711957
MMAR_5119	esxN_2	cell wall and cell processes	9474.66	7531.65	11205.26	5888.52	7235.81	5133.08	0.047038891	0.952961109	0.576583359
MMAR_5149	serB	cell wall and cell processes	422.9	416.64	408.12	586.56	579.16	590.9	0.005998999	0.994001001	1.27706208
MMAR_5154	dppA	cell wall and cell processes	110.62	90.08	106.03	160.17	165.45	158.89	0.00023886	0.99976114	1.438223991
MMAR_5168	MMAR_5168-1	cell wall and cell processes	594.08	650	556.96	837.42	824.17	779.54	7.84E-05	0.999921584	1.228707296
MMAR_5303	MMAR_5303-1	cell wall and cell processes	252.69	165.57	183.78	129.74	108.19	145.79	1.65E-05	0.999983477	0.582844999
MMAR_5368	fbpA	cell wall and cell processes	6757.05	5469.44	5429.84	3437.58	3908.47	3166.11	0	1	0.542401697
MMAR_5375	MMAR_5375-1	cell wall and cell processes	340.49	337.26	332.38	419.13	408.48	423.6	0.003118011	0.996881989	1.125400587
MMAR_5439	MMAR_5439-1	cell wall and cell processes	1217.35	1069.02	1380.03	2085.4	1861.14	1961.11	0.000124143	0.999875857	1.462929116
MMAR_5443	MMAR_5443-1	cell wall and cell processes	689.95	804.22	836.02	1465.09	1186.25	1370.29	0.001381221	0.998618779	1.562749024
MMAR_5445	MMAR_5445-1	cell wall and cell processes	236.58	211.34	243.85	359.61	334.91	320.42	7.64E-07	0.999999236	1.33434882

Conserved hypotheticals - 104 genes

GENE ID	GENE NAME	FUNCTION	FPKM Fragments Per Kilobase of transcript per Million mapped reads.						PPEE	PPDE	RealFC
			NORMAL Short			LSMMG Short			posterior probability that a transcript is equally expressed	posterior probability that a transcript is differentially expressed	real fold change is the ratio of the normalized mean count values for LSMMG over the normalized mean count values for normal
			39.5hrs	40hrs	40.5hrs	39.5hrs	40hrs	40.5hrs			
MMAR_0022	MMAR_0022-1	Conserved hypotheticals	389.08	298.87	276.77	254.91	272.29	251.44	0.013973315	0.986026685	0.737730621
MMAR_0040	MMAR_0040-1	Conserved hypotheticals	43.17	54.08	38.55	66.9	64.67	75.32	0.039100512	0.960899488	1.379583645
MMAR_0119	MMAR_0119-1	Conserved hypotheticals	7.82	15.38	11.11	26.21	20.65	26.14	0.001425111	0.998574889	1.910860701
MMAR_0153	MMAR_0153-1	Conserved hypotheticals	298.68	304.03	320.06	458.05	432.61	485.12	0.005177637	0.994822363	1.343814492
MMAR_0250	MMAR_0250-1	Conserved hypotheticals	76.68	64.78	90.49	116.08	122.2	130.94	0.006022048	0.993977952	1.442974889
MMAR_0259	MMAR_0259-1	Conserved hypotheticals	124.37	117.51	100.16	240.69	240.11	248.54	2.08E-08	0.999999979	1.934561659
MMAR_0337	MMAR_0337-1	Conserved hypotheticals	1846.98	1734.65	1720.96	2406.32	2308.76	2057.9	0.044354493	0.955645507	1.155375071
MMAR_0424	MMAR_0424-1	Conserved hypotheticals	57.89	73.57	85.55	134.09	141.24	131.5	2.22E-06	0.999997781	1.68527989
MMAR_0447	MMAR_0447-1	Conserved hypotheticals	192.26	142.74	177.09	139.72	128.23	120.54	2.70E-06	0.999997305	0.69045165
MMAR_0464	MMAR_0464-1	Conserved hypotheticals	52.69	67.55	52.84	125.9	118.64	130.96	0	1	1.964284684
MMAR_0519	MMAR_0519-1	Conserved hypotheticals	94.3	115.62	109.99	41.65	56.09	39.35	1.04E-10	1	0.385968212
MMAR_0599	MMAR_0599-1	Conserved hypotheticals	736.78	537.66	555.39	443.4	535.04	471	0.000899304	0.999100696	0.72220799
MMAR_0620	lpqJ	Conserved hypotheticals	288.4	280.07	262.95	526.37	455.49	496.06	1.40E-13	1	1.610642628
MMAR_0691	MMAR_0691-1	Conserved hypotheticals	4.85	4.97	7.57	2.07	2.51	2.33	0.00164052	0.99835948	0.359343149
MMAR_0822	MMAR_0822-1	Conserved hypotheticals	577.97	603.97	386.91	913.42	765.91	802.21	0.039565751	0.960434249	1.439899506
MMAR_0827	MMAR_0827-1	Conserved hypotheticals	1032.92	845.53	1046.2	615.38	558.99	675.91	3.15E-08	0.999999969	0.569147392
MMAR_0853	MMAR_0853-1	Conserved hypotheticals	104.81	68.58	49.96	689.09	675.53	846.12	0	1	9.104528741
MMAR_0908	MMAR_0908-1	Conserved hypotheticals	1070.33	890.59	1213.09	684.39	678.78	813.1	0.002039237	0.997960763	0.617806331
MMAR_0982	MMAR_0982-1	Conserved hypotheticals	369.18	366.91	400.44	200.79	187.24	190.54	0	1	0.461871314
MMAR_1215	MMAR_1215-1	Conserved hypotheticals	545.71	567.23	512	662.87	687.72	739.7	0.018461667	0.981538333	1.167969782
MMAR_1534	MMAR_1534-1	Conserved hypotheticals	232.45	266.43	190.08	464.74	403.33	402.59	1.53E-09	0.999999998	1.676482405
MMAR_1572	MMAR_1572-1	Conserved hypotheticals	263.55	279.91	308.86	415.53	446.48	446.91	3.92E-06	0.999996083	1.388802801
MMAR_1579	MMAR_1579-1	Conserved hypotheticals	55.81	44.17	51.63	32.58	19.5	27.04	7.96E-14	1	0.476043219
MMAR_1633	MMAR_1633-1	Conserved hypotheticals	110.35	129.35	187.69	50.78	48.54	47.58	0.000328235	0.999671765	0.307951917
MMAR_1661	MMAR_1661-1	Conserved hypotheticals	134.76	122.79	113.57	213.97	182.77	209.19	2.68E-07	0.999999732	1.485280512
MMAR_1800	MMAR_1800-1	Conserved hypotheticals	883.96	721.38	692.14	417.43	468.81	299.16	4.73E-07	0.999999527	0.461145258
MMAR_1808	MMAR_1808-1	Conserved hypotheticals	347.79	397.44	308.9	518.38	510.6	500.76	1.12E-05	0.99998882	1.313990777
MMAR_1896	MMAR_1896-1	Conserved hypotheticals	184.42	157.34	144.39	106.24	128.79	126.92	4.64E-08	0.999999954	0.678192239
MMAR_2046	MMAR_2046-1	Conserved hypotheticals	54.62	70.68	55.05	89.5	88.2	86.45	0.016807347	0.983192653	1.324353878
MMAR_2098	MMAR_2098-1	Conserved hypotheticals	451.87	403.7	410.52	373.23	422.69	356.94	0.016814539	0.983185461	0.826383521
MMAR_2271	MMAR_2271-1	Conserved hypotheticals	2031.82	1986.71	2762.52	1280.33	1315.26	1502.96	0.030708812	0.969291188	0.541487886
MMAR_2345	MMAR_2345-1	Conserved hypotheticals	110.6	120.5	149.29	188.65	199.17	212.25	0.043319877	0.956680123	1.419852006
MMAR_2354	MMAR_2354-1	Conserved hypotheticals	494.88	560.79	879.12	260.37	252.45	285.55	0.048926752	0.951073248	0.372028563
MMAR_2373	MMAR_2373-1	Conserved hypotheticals	3095.56	4074.96	3602.42	6036.91	6421.98	6410.8	1.64E-07	0.999999836	1.56907165
MMAR_2441	MMAR_2441-1	Conserved hypotheticals	579.85	536.85	659.33	443.34	420.96	435.66	0.000174928	0.999825072	0.658844438
MMAR_2442	MMAR_2442-1	Conserved hypotheticals	254.78	314.58	256.29	437.56	413.03	463.63	4.34E-05	0.999956629	1.437440626
MMAR_2452	MMAR_2452-1	Conserved hypotheticals	53.53	35.08	34.93	30.67	28.21	24.11	0.007033916	0.992966084	0.615495281
MMAR_2715	MMAR_2715-1	conserved hypotheticals	127	96.23	122.37	88.92	90.23	92.49	0.001533944	0.998466056	0.715724352
MMAR_2771	MMAR_2771-1	Conserved hypotheticals	145.16	103	82.05	226.43	182.47	190.14	0.004154543	0.995845457	1.659570191
MMAR_2779	MMAR_2779-1	Conserved hypotheticals	418.68	396.16	341.6	324.22	330.39	242.78	0.005050146	0.994949854	0.705664094
MMAR_2781	MMAR_2781-1	Conserved hypotheticals	281.66	234.27	232.83	443.57	367.35	385.27	2.25E-05	0.999977465	1.452745877
MMAR_2791	MMAR_2791-1	Conserved hypotheticals	74.92	57.22	73.06	127.59	118.08	93.65	0.011780013	0.988219987	1.503920317
MMAR_2850	MMAR_2850-1	Conserved hypotheticals	174.2	189.67	217.93	300.75	298.78	286.04	0.007738421	0.992261579	1.368057381
MMAR_2871	MMAR_2871-1	Conserved hypotheticals	194.54	181.06	299.1	121.83	104.44	83.3	0.007513498	0.992486502	0.409104545
MMAR_2976	MMAR_2976-1	Conserved hypotheticals	182.32	230.94	148.96	322.89	285.05	281.86	0.005276929	0.994723071	1.435435292
MMAR_2980	MMAR_2980-1	Conserved hypotheticals	10.58	7.35	8.12	24.57	26.9	15.85	0.001013458	0.998986542	2.359057837
MMAR_3007	MMAR_3007-1	Conserved hypotheticals	315.82	223.47	223.17	439.44	485.25	464.03	5.00E-15	1	1.664662996
MMAR_3010	MMAR_3010-1	Conserved hypotheticals	50.73	55.22	49.49	89.99	107.75	112.52	8.48E-13	1	1.808365819
MMAR_3068	MMAR_3068-1	Conserved hypotheticals	113.29	109.3	109.65	193.22	197.2	197.53	0	1	1.609589993
MMAR_3069	MMAR_3069-1	Conserved hypotheticals	353.88	400	363.15	705.05	701.61	687.72	0	1	1.694242381
MMAR_3070	MMAR_3070-1	Conserved hypotheticals	113.07	101.75	118.63	251.03	272.6	287.79	0	1	2.210668723
MMAR_3088	MMAR_3088-1	Conserved hypotheticals	149.33	156.6	147.58	286.24	312.05	323.74	0	1	1.844387783
MMAR_3229	MMAR_3229-1	Conserved hypotheticals	2332.35	2288.99	2278.46	3404.6	3092.46	3331.88	0.000597048	0.999402952	1.286018864
MMAR_3230	MMAR_3230-1	Conserved hypotheticals	822.66	830.93	784.11	1306.22	1143.7	1318.02	0.001403777	0.998596223	1.394133509
MMAR_3301	MMAR_3301-1	Conserved hypotheticals	59.01	61.89	45.79	76.37	85.41	72.58	0.03278028	0.96721972	1.278489593
MMAR_3341	MMAR_3341-1	Conserved hypotheticals	60.44	31.12	44	20.86	20.1	25.98	5.12E-05	0.999948764	0.453649882
MMAR_3381	MMAR_3381-1	Conserved hypotheticals	136.53	112.58	94.83	196.41	199.98	160.89	0.000994953	0.999005047	1.479849505
MMAR_3406	MMAR_3406-1	Conserved hypotheticals	188.3	190.49	201.79	130	167.13	144.88	0.000188454	0.999811546	0.69021481
MMAR_3483	MMAR_3483-1	Conserved hypotheticals	11.35	12.36	15.21	22.16	28.85	23.96	0.00013156	0.99986844	1.742297328
MMAR_3487	MMAR_3487-1	Conserved hypotheticals	76.74	67.99	88.01	48.11	50.26	57.53	1.33E-05	0.999986659	0.607637557
MMAR_3492	MMAR_3492-1	Conserved hypotheticals	28.16	45.7	36.04	73.48	335.6	393.09	0.032854784	0.967145216	6.409776252
MMAR_3493	MMAR_3493-1	Conserved hypotheticals	985.2	1276.09	1075.29	1693.13	1666.94	1573.61	0.011818512	0.988181488	1.324358066
MMAR_3549	MMAR_3549-1	Conserved hypotheticals	50.43	45.75	73.08	16.16	12.84	16.53	2.05E-08	0.99999998	0.243859167
MMAR_3627	MMAR_3627-1	Conserved hypotheticals	1259.01	1380.52	1182.11	1080.23	859.04	970.48	0.001685546	0.998314454	0.687398684
MMAR_3725	MMAR_3725-1	Conserved hypotheticals	185.99	145.74	191.68	162.76	135.58	107.38	0.030036635	0.969963365	0.702608065
MMAR_4077	MMAR_4077-1	Conserved hypotheticals	580.07	552.24	529.56	780.88	783.18	787.14	0.00375613	0.99624387	1.283016169
MMAR_4123	MMAR_4123-1	Conserved hypotheticals	1320.23	1340.8	1133.35	1822.19	1667.7	1891.06	0.004110431	0.995889569	1.285990034
MMAR_4142	MMAR_4142-1	Conserved hypotheticals	93.02	88.95	101.18	139.57	131.81	143.5	5.40E-06	0.999994603	1.331196275
MMAR_4177	MMAR_4177-1	Conserved hypotheticals	352.33	259.08	269.76	521.81	577.18	504.89	4.55E-06	0.999995453	1.653775265
MMAR_4191	MMAR_4191-1	Conserved hypotheticals	68.77	69.74	54.34	114.07	103.22	107.79	5.56E-13	1	1.535496387
MMAR_4227	MMAR_4227-1	Conserved hypotheticals	7042.18	6299.63	6634.01	5856.07	6671.71	5666.05	3.43E-05	0.999965689	0.818485487
MMAR_4247	MMAR_4247-1	Conserved hypotheticals	10.16	7.83	11.3	24.17	28.26	40.44	0.003607116	0.996392884	2.877029669
MMAR_4248	MMAR_4248-1	Conserved hypotheticals	15.03	8.54	26.98	73.05	57.16	83.85	1.10E-05	0.999989016	3.81716989
MMAR_4291	MMAR_4291-1	Conserved hypotheticals	396.03	393.46	335.11	530.68	793.66	589.27	0.010109875	0.989890125	1.521926538
MMAR_4297	MMAR_4297-1	Conserved hypotheticals	10430.93	10142.05	7350.46	17274.46	18152.42	13405.7	2.09E-05	0.999979115	1.55859506
MMAR_4306	MMAR_4306-1	Conserved hypotheticals	46369.92	38788.56	16135.57	317119.92	423944.74	258218.46	0	1	8.936271697
MMAR_4312	MMAR_4312-1	Conserved hypotheticals	65.12	47.22	56.19	80.15	94.17	99.19	0.00010574	0.99989426	1.480518303
MMAR_4332	MMAR_4332-1	Conserved hypotheticals	229.62	195.83	178.75	336.82	385.92	298.61	1.64E-06	0.999998362	1.535110951
MMAR_4356	MMAR_4356-1	Conserved hypotheticals	1170.71	933.24	1136.9	782.59	774.46	830.47	6.97E-13	1	0.669121564
MMAR_4498	MMAR_4498-1	Conserved hypotheticals	378.15	225.51	380.72	985.81	1134.82	1652.73	0.000587305	0.999412695	3.476777423
MMAR_4575	MMAR_4575-1	Conserved hypotheticals	388.64	480.96	485	828.38	883.39	929.9	2.88E-13	1	1.759438807
MMAR_4583	MMAR_4583-1	Conserved hypotheticals	77.17	84.25	61.41	119.59	120.16	140.96	2.50E-05	0.999974955	1.550081994
MMAR_4609	MMAR_4609-1	Conserved hypotheticals	363.58	428.2	797.46	1142.77	1300.1	1276.93	0.003807301	0.996192699	2.087610901
MMAR_4619	MMAR_4619-1	Conserved hypotheticals	796.38	911.64	699.92	1307.32	1142.34	1433.01	0.034175721	0.965824279	1.455883703
MMAR_4647	MMAR_4647-1	Conserved hypotheticals	331.82	383.94	475.28	1189.65	1141.21	1637.04	4.20E-07	0.99999958	3.008498535
MMAR_4673	MMAR_4673-1	Conserved hypotheticals	200.43	179.41	164.84	324.35	354.41	329.77	0	1	1.684677369
MMAR_4799	MMAR_4799-1	Conserved hypotheticals	56.47	45.14	64.28	36.65	43.89	39.87	0.026693273	0.973306727	0.658126765
MMAR_4858	MMAR_4858-1	Conserved hypotheticals	113.87	95.51	100.29	66.98	73.66	65.37	0	1	0.606220261
MMAR_4892	MMAR_4892-1	Conserved hypotheticals	321	307.68	298.83	534.34	503.4	488.31	0	1	1.494281087
MMAR_4944	MMAR_4944-1	Conserved hypotheticals	279.27	217.64	200.87	187.36	206.25	174.2	0.024052624	0.975947376	0.743005597
MMAR_5091	MMAR_5091-1	Conserved hypotheticals	1703.71	1374.44	1416.12	2042.83	2691.66	2079.85	0.003359765	0.996640235	1.36460217
MMAR_5103	MMAR_5103-1	Conserved hypotheticals	526.48	535.6	685.57	238.46	252.68	262.85	2.42E-09	0.999999998	0.389795932
MMAR_5106	MMAR_5106-1	Conserved hypotheticals	117.9	109.22	115.41	64.99	51.12	45.59	0	1	0.428620321
MMAR_5142	MMAR_5142-1	Conserved hypotheticals	567.33	505.78	448.89	805.68	750.27	743.82	7.57E-13	1	1.376130254
MMAR_5167	MMAR_5167-1	Conserved hypotheticals	5676.05	4194.39	4560.84	6592.88	9474.18	6735.89	0.00070187	0.99929813	1.404073941
MMAR_5198	MMAR_5198-1	Conserved hypotheticals	867.69	648.98	659.01	1108.04	1114.54	1156.95	3.51E-11	1	1.414220193
MMAR_5209	MMAR_5209-1	Conserved hypotheticals	114.4	117.74	100.22	164.91	173.95	155.27	3.10E-10	1	1.350857418
MMAR_5287	MMAR_5287-1	Conserved hypotheticals	669.58	428.43	525.53	401.2	486.5	367.47	0.004860232	0.995139768	0.701912478
MMAR_5358	MMAR_5358-1	Conserved hypotheticals	150.41	168.06	185.46	279.47	268.66	267.86	7.60E-07	0.99999924	1.464396026
MMAR_5437	MMAR_5437-1	Conserved hypotheticals	587.4	375.1	451.74	812.7	693.61	797.14	0.031618788	0.968381212	1.482739691
MMAR_5446	MMAR_5446-1	Conserved hypotheticals	318.34	271.48	302.03	458.17	430.62	371.39	0.022378919	0.977621081	1.286331415
MMAR_5455	MMAR_5455-1	Conserved hypotheticals	283.54	252.67	254.83	570.3	493.82	473.05	4.44E-16	1	1.770181458
MMAR_5457	espB	Conserved hypotheticals	631.02	522.55	583.13	910.59	828.32	798.6	3.51E-07	0.999999649	1.330533049
MMAR_5574	MMAR_5574-1	Conserved hypotheticals	1478.6	1271.57	1351.5	1239.58	1110.99	1224.75	0.032129484	0.967870516	0.782467862

Information pathway - 60 genes

GENE ID	GENE NAME	FUNCTION	FPKM Fragments Per Kilobase of transcript per Million mapped reads. NORMAL Short			LSMMG Short			PPEE posterior probability that a transcript is equally expressed	PPDE posterior probability that a transcript is differentially expressed	RealFC real fold change is the ratio of the normalized mean count values for LSMMG over the normalized mean count values for normal
			39.5hrs	40hrs	40.5hrs	39.5hrs	40hrs	40.5hrs			
MMAR_0002	dnaN	information pathway	539.32	445.01	465.6	424.7	450.1	409.41	4.08E-11	1	0.806729247
MMAR_0074	rpsR1	information pathway	1748.94	1511.25	1403.05	1167.71	865.82	827.96	5.35E-13	1	0.552528721
MMAR_0076	dnaB	information pathway	551.19	524.04	413.33	835.92	806.58	713.23	2.27E-06	0.999997735	1.441473706
MMAR_0146	MMAR_0146-1	information pathway	57.37	40.51	37.68	65.51	76.87	76.56	0.001975151	0.998024849	1.478323323
MMAR_0744	def	information pathway	508.8	425.42	443.85	582.53	654.89	713.75	0.006911066	0.993088934	1.286659253
MMAR_0957	recB	information pathway	20.51	15.59	18.26	33.6	28.25	28.85	1.55E-05	0.999984495	1.523015842
MMAR_0958	recC	information pathway	18.86	12.89	11.97	22.24	24.11	21.38	0.033472438	0.966527562	1.419972453
MMAR_0967	rpmG2	information pathway	1844.61	1478.6	1680.44	1580.15	1091.07	901.34	0.04029954	0.95970046	0.640906032
MMAR_0972	nusG	information pathway	1829.47	1457.15	1219	1130.18	1130.62	1030.6	0.00322359	0.99677641	0.666572489
MMAR_0974	rplA	information pathway	2007.88	1717.33	1405.03	1092.03	1151.15	1020.39	2.66E-08	0.999999973	0.579372048
MMAR_0990	rplJ	information pathway	1740.78	1587.42	1148.37	801.17	781.01	756.93	2.81E-08	0.999999972	0.475592699
MMAR_0995	rpoB	information pathway	2420.78	2541.19	1823.52	1682.3	1555.26	1548.67	0.009764229	0.990235771	0.642497308
MMAR_1031	rplC	information pathway	4057.27	3067.26	2415.9	1759.29	1773.35	1627.64	0.000135638	0.999864362	0.49442744
MMAR_1034	rplB	information pathway	2951.17	2316.39	1819.46	1342.68	1412.39	1272.44	0.000129399	0.999870601	0.519363953
MMAR_1036	rplV	information pathway	800.9	603.73	449.8	292.74	382.34	268.76	0.001177948	0.998822052	0.464223039
MMAR_1039	rpmC	information pathway	6340.48	5524.74	4480.46	3880.84	3934.34	3218.74	1.68E-10	1	0.606231526
MMAR_1048	rpsN	information pathway	14166.2	12087.11	10434.21	10010.07	8517.29	8636.56	7.67E-07	0.999999233	0.666947386
MMAR_1052	rpsE	information pathway	1314.44	981.69	814.33	615.63	630.32	503.23	5.34E-05	0.999946563	0.513675673
MMAR_1053	rpmD	information pathway	5469.62	4566.56	3144.64	2864.86	3545.12	2371.28	0.037953585	0.962046415	0.597645063
MMAR_1054	rplO	information pathway	3516.14	2783.35	2233.05	1868.95	2183.71	1769.65	0.004726939	0.995273061	0.620496151
MMAR_1085	infA	information pathway	15569.11	12986.82	11523.74	11317.21	8260.47	8598.62	5.25E-05	0.999947487	0.634779724
MMAR_1089	rpsD	information pathway	3325.05	3126.52	2790.11	2354.01	2240.46	2025.18	0	1	0.649576268
MMAR_1090	rpoA	information pathway	2871.65	2266.6	1709.34	1329.33	1246.33	1197.64	0.000641657	0.999358343	0.504162745
MMAR_1091	rplQ	information pathway	1860.86	1752.79	1200.83	1016.42	892.26	849.15	9.97E-05	0.999900264	0.520912673
MMAR_1092	truA	information pathway	95.02	87.48	103.91	58.19	72.21	54.78	8.91E-06	0.99999109	0.58475492
MMAR_1106	rplM	information pathway	6759.78	6708.57	5299.73	4499.7	4492.1	3697.05	1.54E-07	0.999999846	0.610681776
MMAR_1107	rpsI	information pathway	1605.36	1436.96	1069.35	953.58	1096.12	826.59	0.013232347	0.986767653	0.634115796
MMAR_1334	sigH	information pathway	972.9	1165.79	1107.78	1657.12	1735.79	2074.72	0.002459713	0.997540287	1.521122108
MMAR_1344	MMAR_1344-1	information pathway	591.03	481.21	404.77	765.7	724.71	791.73	0.000107739	0.999892261	1.410531268
MMAR_1346	rhlE	information pathway	371.04	335.42	328.18	247.91	269.57	235	0	1	0.661824304
MMAR_1642	nrdE	information pathway	1174.82	1275.96	1458.68	2370.7	2497.59	2982.34	7.49E-07	0.999999251	1.819116489
MMAR_1728	hupB	information pathway	6711.6	4743.48	6045.51	2575.35	3129.66	2372.05	0	1	0.419669995
MMAR_1740	rpmB2_1	information pathway	3264.76	2304.82	2219.44	1504.47	1049.01	1282.92	4.44E-16	1	0.449132851
MMAR_1799	rpsP	information pathway	1642.63	1316	1158.57	937.19	1151.94	921.19	0.000337401	0.999662599	0.664349149
MMAR_1820	tsf	information pathway	1554.04	1395.31	1096.81	877.07	934.16	913.4	2.37E-06	0.999997634	0.613355936
MMAR_1888	proS	information pathway	302.15	266.66	204.88	482.9	478.04	372.4	0.002014144	0.997985856	1.573191372
MMAR_1894	infB	information pathway	561.92	519.18	453.66	397.96	399.73	379.45	1.49E-13	1	0.699080132
MMAR_1897	dinF	information pathway	37.63	35.77	24.05	18.26	22.17	22.39	0.016034126	0.983965874	0.587987546
MMAR_1922	rpsO	information pathway	8023.25	7128.74	6008.53	6415.72	5949.49	5347.14	0.000787206	0.999212794	0.753224683
MMAR_1959	hsdM	information pathway	24.41	37.75	26.11	55.99	51.69	51.99	5.84E-06	0.999994159	1.633167396
MMAR_2011	sigA	information pathway	1453.89	1207.09	1270.18	1175.79	1221.99	1142.93	3.70E-14	1	0.820616501
MMAR_2181	efp	information pathway	1173.4	954.57	947.16	951.45	934.29	826.6	2.40E-06	0.999997601	0.801540978
MMAR_2182	nusB	information pathway	572.36	505.2	471.19	467.33	474.75	390.27	0.000181278	0.999818722	0.779669112
MMAR_2201	mihF	information pathway	3884.46	3112.71	3809.61	2727.88	2695.4	2734.39	6.81E-10	0.999999999	0.680964367
MMAR_2356	ileS	information pathway	189.2	168.39	151.44	156.23	163.01	146.49	0.035259978	0.964740022	0.834363598
MMAR_2369	dnaE1	information pathway	572.69	555.71	510.8	865.64	893.44	839.97	0	1	1.443202669
MMAR_2432	polA	information pathway	300.87	304.47	236	421.14	407.85	366.45	0.004310315	0.995689685	1.293992242
MMAR_2433	rpsA	information pathway	6363.54	6227.9	5392.5	4657.33	4665.45	4520.41	2.22E-16	1	0.70016335
MMAR_2449	rpmL	information pathway	1159.45	640.23	828.15	317.35	541.7	363.66	1.75E-09	0.999999998	0.417495911
MMAR_2450	rplT	information pathway	2613.97	1985.55	1902.26	1448.63	1677.65	1198.77	4.37E-08	0.999999956	0.602612487
MMAR_2585	tex	information pathway	54.1	35.75	37.89	58.18	67.14	69.49	0.038437526	0.961562474	1.397070496
MMAR_4069	dinG	information pathway	27.69	18.15	20.02	37.64	40.28	32.82	0.001043846	0.998956154	1.539856901
MMAR_4189	deaD	information pathway	532.24	474.66	450.55	732.64	697.38	678.36	0	1	1.317853934
MMAR_4387	greA	information pathway	1082.81	1011.29	937.55	858.74	937	774.72	1.23E-07	0.999999877	0.766752107
MMAR_4472	rplY	information pathway	1182.96	1137.1	952.63	642.55	719.67	635.69	0	1	0.553776137
MMAR_4482	MMAR_4482-1	information pathway	255.99	270.32	279.73	358.21	370.73	361.06	0.00863497	0.99136503	1.222076632
MMAR_4553	uvrD1	information pathway	197.57	169.63	155.75	265.71	257.79	237.98	5.41E-08	0.999999946	1.328468479
MMAR_5102	lysS	information pathway	815.27	824.98	566.03	1380.57	1316.46	1315.79	2.15E-12	1	1.656923325
MMAR_5568	rpmH	information pathway	1170.62	842.39	491.12	366.69	181.89	177.2	2.94E-05	0.999970633	0.271895869
MMAR_5569	rnpA	information pathway	927.89	779.72	670.36	555.62	444.28	498.54	5.92E-11	1	0.572091113

Insertion seqs and phages -1 gene

GENE ID	GENE NAME	FUNCTION	FPKM Fragments Per Kilobase of transcript per Million mapped reads. NORMAL Short			LSMMG Short			PPEE posterior probability that a transcript is equally expressed	PPDE posterior probability that a transcript is differentially expressed	RealFC real fold change is the ratio of the normalized mean count values for LSMMG over the normalized mean count values for normal
			39.5hrs	40hrs	40.5hrs	39.5hrs	40hrs	40.5hrs			
MMAR_2506	xerD	insertion seQ and phages	186.44	207.46	148.03	282.58	287.89	277.94	1.32E-07	0.999999868	1.420579424

Intermediary metabolism and respiration - 162 genes

GENE ID	GENE NAME	FUNCTION	FPKM<br>Fragments Per Kilobase of transcript per Million mapped reads.						PPEE	PPDE	RealFC
			NORMAL Short			LSMMG Short			posterior probability that a transcript is equally expressed	posterior probability that a transcript is differentially expressed	real fold change is the ratio of the normalized mean count values for LSMMG over the normalized mean count values for normal
			39.5hrs	40hrs	40.5hrs	39.5hrs	40hrs	40.5hrs			
MMAR_0107	celA	Intermediary metabolism and respiration	221.65	160.38	144.46	101.02	122.83	80.86	0.000429018	0.999570982	0.52908348
MMAR_0158	icd2	Intermediary metabolism and respiration	1013.59	1210.3	944.75	1715.8	1643.3	1750.26	1.79E-09	0.999999998	1.46317322
MMAR_0210	gcvT_1	Intermediary metabolism and respiration	23.21	32.69	30.54	18.92	17.2	17.54	0.012506177	0.987493823	0.55982638
MMAR_0211	gcvB_1	Intermediary metabolism and respiration	24.36	25.58	18.7	17.81	17.81	17.4	0.043380527	0.956619473	0.703202429
MMAR_0251	mtn	Intermediary metabolism and respiration	106.11	73.63	102.46	163.74	166.42	143.09	1.70E-05	0.999983036	1.525946929
MMAR_0260	MMAR_0260-1	Intermediary metabolism and respiration	140.91	218.06	155.99	367.19	429.14	324.38	3.83E-07	0.999999617	1.957671499
MMAR_0312	MMAR_0312-1	Intermediary metabolism and respiration	188.9	154.78	182.95	235.33	242.87	249.89	3.22E-07	0.999999678	1.259022074
MMAR_0324	pepA	Intermediary metabolism and respiration	253.59	258.91	293.86	212.08	220.08	190.51	0.00142137	0.99857863	0.69907866
MMAR_0335	MMAR_0335-1	Intermediary metabolism and respiration	65.13	72.3	49.18	225.55	248.34	172.52	8.23E-14	1	3.14392322
MMAR_0347	msrA	Intermediary metabolism and respiration	299.97	280.48	300.42	420.96	419.25	476.09	0.000363207	0.999636793	1.352441921
MMAR_0357	MMAR_0357-1	Intermediary metabolism and respiration	275.29	244.13	344.05	193.93	208.63	166.76	0.006240574	0.993759426	0.596794134
MMAR_0377	pntAa	Intermediary metabolism and respiration	134.74	116.8	95.51	87.16	88.18	90.91	0.000641651	0.999358349	0.699831681
MMAR_0378	pntAb	Intermediary metabolism and respiration	2247.13	1704.28	1668.17	1412.24	1514.48	1462.84	1.25E-11	1	0.708392798
MMAR_0379	pntB	Intermediary metabolism and respiration	356.26	315.11	268.85	264.6	281.1	243.83	0.011583329	0.988416671	0.76545895
MMAR_0432	ilvD	Intermediary metabolism and respiration	391.79	346.57	322.95	327.56	310.61	323.95	9.79E-07	0.999999021	0.826194877
MMAR_0490	gabD1	Intermediary metabolism and respiration	257.91	236.12	206.56	303.88	308.32	308.84	1.84E-05	0.999981636	1.19740919
MMAR_0516	nirB	Intermediary metabolism and respiration	59.42	54.41	27.94	18.03	21.27	20.17	0.014801062	0.985198938	0.383987473
MMAR_0554	MMAR_0554-1	Intermediary metabolism and respiration	40.88	33.42	39.72	63.81	73.49	56.68	9.03E-05	0.9999097	1.547669651
MMAR_0611	MMAR_0611-1	Intermediary metabolism and respiration	888.85	794.73	852.59	789.53	789	821.88	2.69E-11	1	0.861580882
MMAR_0661	MMAR_0661-1	Intermediary metabolism and respiration	75.64	110.38	94.94	135.03	169.16	195.39	0.022052345	0.977947655	1.598570037
MMAR_0709	fgd1	Intermediary metabolism and respiration	277.86	241.9	220.77	353.25	348.72	390.59	1.22E-06	0.999998782	1.344252621
MMAR_0710	pta	Intermediary metabolism and respiration	149.9	138.05	121.05	300.09	305.57	272.25	0	1	1.955704082
MMAR_0711	ackA	Intermediary metabolism and respiration	114.4	94.23	91.95	195.92	216.89	176.54	3.89E-15	1	1.786620452
MMAR_0726	lpqL	Intermediary metabolism and respiration	498.65	472.12	438.46	726.61	774.88	677.9	3.33E-16	1	1.406578848
MMAR_0727	lpqL_1	Intermediary metabolism and respiration	178.08	187.84	151.82	302.25	238.46	251.36	0.024666104	0.975333896	1.39082981
MMAR_0728	lpqM	Intermediary metabolism and respiration	180.6	144.85	162.68	227.8	249.69	212.8	0.00030108	0.99969892	1.288239698
MMAR_0785	lpd	Intermediary metabolism and respiration	1794.32	1720.78	1467.38	2394.03	2339.85	2310.06	7.33E-12	1	1.286706159
MMAR_0843	hemC	Intermediary metabolism and respiration	2107.34	1458.17	1285.33	885.82	916.18	752.35	0.000131245	0.999868755	0.482246393
MMAR_0917	MMAR_0917-1	Intermediary metabolism and respiration	71.8	73.01	69.65	93.14	111.43	94.9	0.006558286	0.993441714	1.267484175
MMAR_0934	MMAR_0934-1	Intermediary metabolism and respiration	125.62	130.18	124.38	212.33	224.36	197.48	1.67E-06	0.999998331	1.512026904
MMAR_0981	lipG1	Intermediary metabolism and respiration	77.65	82.66	80.99	64.85	68.51	43.34	0.028572825	0.971427175	0.662461125
MMAR_1041	atsA	Intermediary metabolism and respiration	302.9	263.82	228.67	411	430.73	402.85	4.45E-14	1	1.427743532
MMAR_1071	adk	Intermediary metabolism and respiration	1378.4	885.44	1093.83	877.18	984.09	748.47	0.023296749	0.976703251	0.708149615
MMAR_1082	rmlB	Intermediary metabolism and respiration	427.02	420.38	354.16	529.87	543.16	493.69	0.002382623	0.997617377	1.184810824
MMAR_1108	mrsA	Intermediary metabolism and respiration	239.02	208.22	186.73	152.02	142.88	119.57	2.14E-13	1	0.595896522
MMAR_1171	bioF2_5	Intermediary metabolism and respiration	34.55	28.53	28.78	56.03	63.28	73.3	2.59E-11	1	1.91243193
MMAR_1176	folD	Intermediary metabolism and respiration	612.74	583.7	471.11	801.37	830.47	688.92	0.033564465	0.966435535	1.264999516
MMAR_1186	icd1	Intermediary metabolism and respiration	263.69	224.37	210.02	357.36	328.04	370.65	3.82E-07	0.999999618	1.379389348
MMAR_1200	sdhB	Intermediary metabolism and respiration	694.04	532.47	725.29	423.11	439.4	403.01	1.27E-08	0.999999987	0.58899283
MMAR_1202	sdhD	Intermediary metabolism and respiration	328.16	275.38	345.8	190.36	188.26	202.05	3.24E-13	1	0.553401889
MMAR_1203	sdhC	Intermediary metabolism and respiration	2128.86	2064.88	2410.59	1256.8	1555.14	1118.01	7.13E-07	0.999999287	0.531307336
MMAR_1216	MMAR_1216-1	Intermediary metabolism and respiration	157.86	154.54	157.52	204.3	247.13	260.71	0.000870909	0.999129091	1.374190066
MMAR_1220	amiB1	Intermediary metabolism and respiration	159.49	133.37	122.97	110.21	122.23	121.8	0.002197481	0.997802519	0.776773439
MMAR_1232	atsB	Intermediary metabolism and respiration	70.15	66.96	70.67	88.47	92.38	102.49	0.001564537	0.998435463	1.239859426
MMAR_1251	accA3	Intermediary metabolism and respiration	457.41	436.74	509.68	242.27	294.33	272.85	1.49E-14	1	0.5232284
MMAR_1295	sahH	Intermediary metabolism and respiration	5534.74	5181.03	5504.67	4879.22	5271.07	4460.83	0.020643442	0.979356558	0.818280329
MMAR_1333	MMAR_1333-1	Intermediary metabolism and respiration	333.57	278.5	282.57	353.17	418.3	420.87	0.045021725	0.954978275	1.212925399
MMAR_1342	entC	Intermediary metabolism and respiration	87.9	106.5	81.29	146.25	151.83	150.41	3.69E-10	1	1.473357008
MMAR_1343	gpm2	Intermediary metabolism and respiration	156.97	133.9	144.69	250.28	275.12	258.55	0.001240303	0.998759697	1.633358119
MMAR_1363	MMAR_1363-1	Intermediary metabolism and respiration	1855.23	1808.8	1802.15	2561.88	2955.18	2400.09	1.38E-05	0.999986243	1.289311239
MMAR_1375	atsD_1	Intermediary metabolism and respiration	78.86	67.2	62.91	93.97	113.93	124.91	0.000922143	0.999077857	1.452480712
MMAR_1575	MMAR_1575-1	Intermediary metabolism and respiration	195.62	221.73	189.64	332.82	346.69	388.91	1.01E-10	1	1.593631689
MMAR_1580	MMAR_1580-1	Intermediary metabolism and respiration	85.98	96.91	117.21	30.58	24.52	25.31	4.33E-15	1	0.242321397
MMAR_1644	MMAR_1644-1	Intermediary metabolism and respiration	306.83	301.56	274.64	583.31	576.25	557.97	0	1	1.768467068
MMAR_1685	fixB	Intermediary metabolism and respiration	506.25	354.98	431.74	294.13	370.77	316.52	0.000344228	0.999655772	0.69213026
MMAR_1689	iscS	Intermediary metabolism and respiration	119.31	110.53	96.37	76.46	75.83	70.13	0.022179823	0.977820177	0.620614086
MMAR_1714	MMAR_1714-1	Intermediary metabolism and respiration	40.13	44.38	63.43	17.33	15.53	9.26	1.64E-06	0.999998359	0.256969015
MMAR_1724	bioF2_2	Intermediary metabolism and respiration	38.75	35.88	52.69	23.19	29.8	28.08	0.026838248	0.973161752	0.576252191
MMAR_1726	leuC	Intermediary metabolism and respiration	375.5	292.02	367.74	126.87	163.37	185.45	0	1	0.418360819
MMAR_1727	leuD	Intermediary metabolism and respiration	556.04	429.41	484.55	224.3	296.74	322.27	0	1	0.521908003
MMAR_1744	MMAR_1744-1	Intermediary metabolism and respiration	355.66	248.9	332.28	202.72	223.13	182.75	1.31E-08	0.999999987	0.591462408
MMAR_1758	MMAR_1758-1	Intermediary metabolism and respiration	252.71	226.4	189.13	292.26	311.48	282.1	0.026719977	0.973280023	1.207795731
MMAR_1766	MMAR_1766-1	Intermediary metabolism and respiration	1466.7	1257.09	1332.51	1920.66	1769.12	1664.22	0.004028967	0.995971033	1.199747043
MMAR_1821	amiC	Intermediary metabolism and respiration	201.6	178.02	129.64	124.19	103.44	122.68	0.013779312	0.986220688	0.628486413
MMAR_1997	MMAR_1997-1	Intermediary metabolism and respiration	232.68	154.1	201.41	154.78	154.65	139.01	0.00016467	0.99983533	0.695978236
MMAR_2017	dut	Intermediary metabolism and respiration	335.97	245.56	195.07	192.31	170.72	164.83	0.022392732	0.977607268	0.621104994
MMAR_2022	ceoC	Intermediary metabolism and respiration	660.69	792.88	648.32	1154.83	1085.57	1044.96	6.93E-07	0.999999307	1.411282606
MMAR_2176	aroK	Intermediary metabolism and respiration	470.83	367.94	348.03	335.85	338.38	259.4	0.002215509	0.997784491	0.715500546
MMAR_2202	gmk	Intermediary metabolism and respiration	1050.82	920.63	780.19	673.9	764.88	595.34	7.34E-05	0.999926622	0.671355938
MMAR_2207	MMAR_2207-1	Intermediary metabolism and respiration	50.55	62.42	48.07	85.91	79.2	79.94	0.000102165	0.999897835	1.37898526
MMAR_2269	csd	Intermediary metabolism and respiration	358.23	346.76	485.16	224.89	196.39	226.17	0.000324776	0.999675224	0.492537349
MMAR_2282	acn	Intermediary metabolism and respiration	1136.55	1168.72	1479.05	686.96	756.31	725.19	9.19E-05	0.999908071	0.519187407
MMAR_2333	wcaA	Intermediary metabolism and respiration	315.4	274.52	384.09	247.94	242.56	225.83	0.020917899	0.979082101	0.666419874
MMAR_2353	MMAR_2353-1	Intermediary metabolism and respiration	75.21	86.72	125.93	48.14	39.09	41.7	0.005554815	0.994445185	0.403944265
MMAR_2362	MMAR_2362-1	Intermediary metabolism and respiration	197.5	201.76	168.72	304.19	264.62	258.96	0.000668408	0.999331592	1.323289877
MMAR_2379	treX	Intermediary metabolism and respiration	237.2	204.94	217.68	288.8	303.83	293.43	0.034849772	0.965150228	1.222937413
MMAR_2557	MMAR_2557-1	Intermediary metabolism and respiration	40.15	50.11	35.75	71.2	90.31	76.54	4.72E-07	0.999999528	1.710014521
MMAR_2568	MMAR_2568-1	Intermediary metabolism and respiration	60.85	61.11	64.36	97.66	97.31	106.22	1.22E-15	1	1.468115848
MMAR_2571	gabD2	Intermediary metabolism and respiration	301.27	285.73	327.18	180.92	226.42	220.34	1.65E-08	0.999999984	0.623087483
MMAR_2648	MMAR_2648-1	Intermediary metabolism and respiration	399.64	351.92	338.88	531.84	486.03	454.03	0.002476358	0.997523642	1.229207658
MMAR_2708	gcvB	Intermediary metabolism and respiration	161.41	148.71	147.76	129.32	140.19	115.72	3.37E-07	0.999999663	0.765834716
MMAR_2713	glcB	Intermediary metabolism and respiration	1202.58	1199.22	1033.09	1035.13	1074.98	1008.65	0.005716405	0.994283595	0.826073783
MMAR_2782	adhC	Intermediary metabolism and respiration	40.83	53.86	45.75	99.39	91.13	113.89	3.74E-09	0.999999996	1.95833048
MMAR_2813	MMAR_2813-1	Intermediary metabolism and respiration	19.54	26.45	22.95	32.09	36.64	41.73	0.018444928	0.981555072	1.44771219
MMAR_2844	MMAR_2844-1	Intermediary metabolism and respiration	405.38	358.66	369.05	170.99	200.54	203.49	0	1	0.461730443
MMAR_2924	MMAR_2924-1	Intermediary metabolism and respiration	259.23	269.04	375.34	178.37	154.76	178.75	0.007418759	0.992581241	0.511172891
MMAR_2992	folE_1	Intermediary metabolism and respiration	216.64	282.06	329.19	428.87	498.58	524.88	0.01168728	0.98831272	1.576794904
MMAR_3085	prcB	Intermediary metabolism and respiration	464.3	435.06	420.07	529.78	615.36	570.16	0.000900785	0.999099215	1.180267034
MMAR_3101	lpd_1	Intermediary metabolism and respiration	129.33	141.82	149.94	187.25	197.42	187.24	0.033984318	0.966015682	1.229755402
MMAR_3112	cysQ	Intermediary metabolism and respiration	99.85	83.75	89.02	66.32	60.72	86.91	0.018211181	0.981788819	0.714674041
MMAR_3123	MMAR_3123-1	Intermediary metabolism and respiration	164.43	220.16	157.91	328.62	284.93	314.69	2.82E-05	0.999971761	1.548838775
MMAR_3202	MMAR_3202-1	Intermediary metabolism and respiration	181.01	155.41	137.51	134.17	138.33	123.12	0.000859324	0.999140676	0.760666588
MMAR_3246	cbhK	Intermediary metabolism and respiration	1090.86	1012.54	932.44	946.17	997.58	982.31	0.000160917	0.999839083	0.876312832
MMAR_3289	glnA1	Intermediary metabolism and respiration	1936.92	1898.69	2013.76	1084.08	1225.74	1284.39	0	1	0.55772064
MMAR_3294	glnA2	Intermediary metabolism and respiration	827.39	916.1	884.28	565.51	536.6	540.09	7.77E-16	1	0.566321303
MMAR_3296	ctaD_1	Intermediary metabolism and respiration	18.15	16.71	21.71	29.28	35.93	30.48	0.01112392	0.98887608	1.534176305
MMAR_3314	MMAR_3314-1	Intermediary metabolism and respiration	157.28	114.76	136.84	174.41	194.97	201.39	0.021198448	0.978801552	1.273213447
MMAR_3334	aceE	Intermediary metabolism and respiration	472.58	415.18	376.15	537.93	575.46	521.35	0.018066012	0.981933988	1.179582296
MMAR_3388	MMAR_3388-1	Intermediary metabolism and respiration	319.39	346.6	259.33	650.56	519.23	542.18	3.97E-06	0.999996034	1.682094198
MMAR_3409	MMAR_3409-1	Intermediary metabolism and respiration	8	9.75	8.81	25.41	69.33	57.15	0.001053497	0.998946503	5.159124064
MMAR_3410	MMAR_3410-1	Intermediary metabolism and respiration	31.48	21.72	36.31	87.07	199.34	118.6	0.041343093	0.958656907	4.113448426
MMAR_3489	MMAR_3489-1	Intermediary metabolism and respiration	6.54	8.92	3.74	13.44	28.61	19	0.008726335	0.991273665	2.875324559
MMAR_3558	MMAR_3558-1	Intermediary metabolism and respiration	264.64	405.73	622.76	90.36	96.75	136.67	0.032993367	0.967006633	0.224675241
MMAR_3639	fdhF	Intermediary metabolism and respiration	22.11	22.58	19.57	13.68	13.68	13.67	0.049522182	0.950477818	0.580909674
MMAR_3645	cysK1	Intermediary metabolism and respiration	251.56	280.19	229.51	213.75	215.28	214.57	0.014849446	0.985150554	0.766227909
MMAR_3721	MMAR_3721-1	Intermediary metabolism and respiration	401.92	441.31	402.98	600	579.87	618.42	8.44E-09	0.999999992	1.309682387
MMAR_3730	MMAR_3730-1	Intermediary metabolism and respiration	355.76	395.56	377.16	322	327.74	322.08	0.014609539	0.985390461	0.77896353
MMAR_3757	MMAR_3757-1	Intermediary metabolism and respiration	186.8	183.15	182.77	233.94	224.3	241.8	0.004074309	0.995925691	1.151305799
MMAR_3801	mobA	Intermediary metabolism and respiration	234.51	326.96	323.53	83.9	100.36	137.57	6.88E-07	0.999999312	0.326915719
MMAR_3802	porB	Intermediary metabolism and respiration	863.16	916.04	1205.83	284.36	351.94	442.96	1.41E-07	0.999999859	0.326753942
MMAR_3803	porA	Intermediary metabolism and respiration	832.88	1038.84	1258.08	306.8	317.54	433.09	1.46E-06	0.999998542	0.305096991
MMAR_3817	pepN	Intermediary metabolism and respiration	465.69	388.73	345.44	540.57	579.04	548.48	0.000461661	0.999538339	1.269469957
MMAR_3845	citE	Intermediary metabolism and respiration	182.56	168.96	196.77	92.81	114.12	96.51	0	1	0.501223511
MMAR_3846	MMAR_3846-1	Intermediary metabolism and respiration	165.57	180.2	232.16	102.48	103.13	89.47	0.000151325	0.999848675	0.458236989
MMAR_4076	gloA_1	Intermediary metabolism and respiration	1509.23	1314.88	1659.82	1200.14	1162.12	1122.16	0.00034991	0.99965009	0.701660196
MMAR_4097	hemK	Intermediary metabolism and respiration	253.44	202.85	179.77	186.83	175.94	179.2	0.041325195	0.958674805	0.777732885
MMAR_4180	MMAR_4180-1	Intermediary metabolism and respiration	288.02	335.39	263.84	450.45	488.37	447.52	6.40E-09	0.999999994	1.415873852
MMAR_4188	MMAR_4188-1	Intermediary metabolism and respiration	153.9	117.24	115.87	190.36	212.22	192.88	1.40E-07	0.99999986	1.404652328
MMAR_4197	mez_1	Intermediary metabolism and respiration	172.44	152.01	145.97	110.79	119.67	124.67	0.029704567	0.970295433	0.68734191
MMAR_4214	htrA	Intermediary metabolism and respiration	400.57	401.16	384.97	396.56	387.61	375.54	0.045974276	0.954025724	0.88769356
MMAR_4225	glgC	Intermediary metabolism and respiration	298.77	343.98	310.98	503.43	480.67	454.88	1.49E-07	0.999999851	1.366438913
MMAR_4246	aldA_1	Intermediary metabolism and respiration	3.22	5.18	4.51	9.86	10.78	15.13	0.000480179	0.999519821	2.494140372
MMAR_4252	putA	Intermediary metabolism and respiration	6.39	0.94	4.44	8.8	18.24	23.62	0.031129018	0.968870982	3.986695824
MMAR_4253	rocA	Intermediary metabolism and respiration	15.43	9.7	13.88	37.89	29.38	33.88	2.45E-10	1	2.370451936
MMAR_4279	fbiC	Intermediary metabolism and respiration	141.14	147.83	126.96	208.63	208.94	238.25	3.87E-09	0.999999996	1.433601914
MMAR_4326	MMAR_4326-1	Intermediary metabolism and respiration	25.07	18.81	24.34	42.16	56.56	81.04	0.019622277	0.980377723	2.399826898
MMAR_4339	zwf1	Intermediary metabolism and respiration	257.73	222.63	214.6	319.4	301.17	328.15	2.62E-06	0.999997378	1.24410794
MMAR_4372	phoH2	Intermediary metabolism and respiration	214.31	168.69	221.62	288.32	297.42	301.6	9.28E-05	0.999907234	1.335178979
MMAR_4376	coaA	Intermediary metabolism and respiration	303.2	239.27	264.75	407.46	376.38	383.34	1.15E-08	0.999999989	1.317243616
MMAR_4390	cbs	Intermediary metabolism and respiration	337.76	320.97	280.29	403.4	407.38	407.66	1.75E-06	0.999998249	1.18088838
MMAR_4409	MMAR_4409-1	Intermediary metabolism and respiration	362.16	371.86	412.52	575.43	572.29	603	8.04E-07	0.999999196	1.382226356
MMAR_4473	pth	Intermediary metabolism and respiration	813.78	639.67	533.52	350.09	349.38	369.71	8.80E-10	0.999999999	0.491108959
MMAR_4497	MMAR_4497-1	Intermediary metabolism and respiration	28.6	30.94	25	88.27	98.01	120.29	1.11E-16	1	3.287569734
MMAR_4542	purH	Intermediary metabolism and respiration	245.57	219.33	200.39	269.79	316.11	293.98	0.009025557	0.990974443	1.204641233
MMAR_4549	sucD	Intermediary metabolism and respiration	968.28	749.85	857.92	669.19	799.44	677.57	0.0001753	0.9998247	0.758305862
MMAR_4550	sucC	Intermediary metabolism and respiration	1464.01	1446.88	1401.12	1199.83	1318.44	1309.97	2.25E-08	0.999999977	0.80599517
MMAR_4557	pgi	Intermediary metabolism and respiration	303.63	327.04	341.67	514.78	470.08	490.5	2.76E-06	0.999997238	1.37555038
MMAR_4614	MMAR_4614-1	Intermediary metabolism and respiration	103.64	104.95	108.89	177.48	165.26	193.68	2.28E-09	0.999999998	1.53367897
MMAR_4640	gltA2	Intermediary metabolism and respiration	1195.07	1075.83	1105.31	1391.65	1486.1	1379.57	1.01E-09	0.999999999	1.146685172
MMAR_4646	fprB	Intermediary metabolism and respiration	32.78	32.01	37.84	81.5	85.89	97.73	0	1	2.343513243
MMAR_4678	MMAR_4678-1	Intermediary metabolism and respiration	132.65	156.84	137.51	188.61	192.74	185.95	0.028577117	0.971422883	1.202736693
MMAR_4713	MMAR_4713-1	Intermediary metabolism and respiration	9.71	5.76	9.79	1.03	2.71	6.31	0.008484068	0.991515932	0.363637541
MMAR_4770	MMAR_4770-1	Intermediary metabolism and respiration	159.86	196.61	161.43	261.11	271.99	274.64	1.11E-08	0.999999989	1.412814447
MMAR_4825	metH	Intermediary metabolism and respiration	127.05	139.84	143.98	101.79	99.73	91.49	6.32E-07	0.999999368	0.646917655
MMAR_4908	ptrB	Intermediary metabolism and respiration	399.25	402.94	350.6	620.57	600.6	639.94	0	1	1.468530699
MMAR_4915	cyp126A3	Intermediary metabolism and respiration	73.6	63.81	45.94	101.09	93.09	88.93	0.009342906	0.990657094	1.41057999
MMAR_4921	moeY	Intermediary metabolism and respiration	273.27	240.04	214.98	169.38	175.26	195.69	2.02E-10	1	0.676646517
MMAR_5036	MMAR_5036-1	Intermediary metabolism and respiration	63.52	60.72	74.72	39.97	35.86	44.35	3.15E-07	0.999999685	0.547472405
MMAR_5042	MMAR_5042-1	Intermediary metabolism and respiration	31.33	44.81	38.23	19.03	23.06	24.43	0.003529392	0.996470608	0.524427437
MMAR_5043	fdxB	Intermediary metabolism and respiration	366.62	370.97	422.08	284.47	321.12	289.57	0.001057987	0.998942013	0.700012996
MMAR_5082	ispD	Intermediary metabolism and respiration	431.67	386.13	446.69	342.57	339.85	363.16	1.66E-06	0.999998336	0.749678911
MMAR_5104	panD	Intermediary metabolism and respiration	1070.97	1314.64	1358.78	672.64	617.23	661.51	3.86E-06	0.99999614	0.467143064
MMAR_5105	panC	Intermediary metabolism and respiration	204.99	172.36	218.58	114.76	125.19	124.11	6.47E-14	1	0.554685839
MMAR_5155	acs	Intermediary metabolism and respiration	126.79	91.17	106.19	147.83	159.5	160.47	4.97E-05	0.999950343	1.318025299
MMAR_5194	MMAR_5194-1	Intermediary metabolism and respiration	157.42	144.1	111.58	265.23	257.91	275.25	1.83E-06	0.999998172	1.757785265
MMAR_5221	asd	Intermediary metabolism and respiration	226.75	180.26	204.91	157.41	197.39	170.62	0.007781527	0.992218473	0.781479071
MMAR_5235	MMAR_5235-1	Intermediary metabolism and respiration	202.25	246.11	249.86	337.92	327.75	337.6	0.030786813	0.969213187	1.29916956
MMAR_5252	MMAR_5252-1	Intermediary metabolism and respiration	212	151.2	170.14	285.87	311.63	252.84	0.000133048	0.999866952	1.454874259
MMAR_5314	MMAR_5314-1	Intermediary metabolism and respiration	157.74	138.77	132.74	187.6	217.45	213.12	8.28E-08	0.999999917	1.311954486
MMAR_5353	MMAR_5353-1	Intermediary metabolism and respiration	456.97	370.12	336.83	335.99	335.43	335.91	0.004606375	0.995393625	0.789105292
MMAR_5390	pheA	Intermediary metabolism and respiration	32.99	48.83	43.11	25.43	25.76	26.98	0.028969063	0.971030937	0.563365992
MMAR_5394	glpQ1	Intermediary metabolism and respiration	329.6	294.75	263.03	252.72	279.51	257.02	0.006862689	0.993137311	0.809065943

Lipid metabolism - 56 genes

GENE ID	GENE NAME	FUNCTION	FPKM Fragments Per Kilobase of transcript per Million mapped reads.						PPEE	PPDE	RealFC
			NORMAL Short			LSMMG Short			posterior probability that a transcript is equally expressed	posterior probability that a transcript is differentially expressed	real fold change is the ratio of the normalized mean count values for LSMMG over the normalized mean count values for normal
			39.5hrs	40hrs	40.5hrs	39.5hrs	40hrs	40.5hrs			
MMAR_0064	MMAR_0064-1	Lipid metabolism	146.39	129.97	147.63	88.3	61.87	84.51	2.22E-16	1	0.503196232
MMAR_0256	MMAR_0256-1	Lipid metabolism	23.41	25.64	19.03	45.04	48.1	41.28	0	1	1.795881373
MMAR_0336	desA3_2	Lipid metabolism	799.05	781.14	550.36	2991.94	3270.43	2810.16	0	1	3.877319134
MMAR_0374	fadE2	Lipid metabolism	303.05	280.81	290.17	396.75	466.76	397.01	7.12E-05	0.999928809	1.310060788
MMAR_0455	fadE3_2	Lipid metabolism	44.55	54.24	43.86	86.58	92.66	86.95	0.009002615	0.990997385	1.689121903
MMAR_0488	fadE4	Lipid metabolism	901	881.51	781.97	783.38	843.65	783.35	0.024607089	0.975392911	0.854873436
MMAR_0530	fadE6	Lipid metabolism	252.87	244.76	242.64	337.88	359.99	349.15	2.22E-16	1	1.285857499
MMAR_0707	pks6	Lipid metabolism	34.72	36.89	39.07	26.86	26.04	26.25	4.00E-09	0.999999996	0.649371628
MMAR_0851	MMAR_0851-1	Lipid metabolism	7.81	7.97	9.38	6.34	5.66	5.71	3.30E-05	0.999966977	0.638867741
MMAR_1140	choD	Lipid metabolism	175.87	125.53	150.79	122.98	139.5	117.74	0.012408621	0.987591379	0.767581961
MMAR_1315	desA3	Lipid metabolism	366.28	313.85	361.51	712.88	730.15	1072.89	0.00464526	0.99535474	2.197855811
MMAR_1316	desA3_1	Lipid metabolism	554.1	559.68	498.43	1153.59	1088.33	1390.65	7.43E-11	1	2.048007031
MMAR_1509	fadE23	Lipid metabolism	566.79	521.15	468.32	845.15	955.62	859.05	0	1	1.555110295
MMAR_1510	fadE24	Lipid metabolism	222.8	219.33	186.83	299.1	321.51	354.55	8.76E-08	0.999999912	1.410301803
MMAR_1732	gpdA2	Lipid metabolism	144.45	150.28	142.9	183.64	214.6	192.65	0.007841045	0.992158955	1.223644918
MMAR_1759	fadD29	Lipid metabolism	2231.51	2299.29	2102.53	2041.02	2005.92	1891.66	4.44E-05	0.99995561	0.813397432
MMAR_1761	fadD22	Lipid metabolism	920.24	845.05	814.14	645.69	684.96	610.33	0	1	0.685002795
MMAR_1762	pks15/1	Lipid metabolism	435.33	384.37	352.88	230.85	237.74	210.25	0	1	0.528152873
MMAR_1767	mas	Lipid metabolism	777.75	756.22	698.15	684.9	689.16	666.13	4.77E-13	1	0.83217224
MMAR_1778	tesA	Lipid metabolism	2739.67	3530.55	2653.36	5373	4416.81	4538.91	0.031668089	0.968331911	1.45034145
MMAR_2225	lprH	Lipid metabolism	105.34	135.1	119.99	182.03	197.2	167.28	0.003898174	0.996101826	1.364614067
MMAR_2233	fadD12	Lipid metabolism	109.48	84.58	96.69	145.43	160.4	159.82	2.53E-05	0.999974656	1.460388945
MMAR_2370	fadD11	Lipid metabolism	335.16	337.94	313.93	469.08	444.02	467.46	1.19E-13	1	1.271308332
MMAR_2371	plsB1	Lipid metabolism	159.04	164.66	151.06	243.21	229.73	224.29	9.33E-15	1	1.334235528
MMAR_2470	pks10	Lipid metabolism	251.87	259.04	265.45	221.54	233.31	244.86	0.029659232	0.970340768	0.81695914
MMAR_2625	fadD1	Lipid metabolism	53.43	34.09	38.12	64.76	64.06	61.08	0.024046733	0.975953267	1.384748862
MMAR_2981	fabG3_1	Lipid metabolism	66.18	43.7	43.25	110.13	135.34	101.1	2.38E-07	0.999999762	2.06989727
MMAR_3231	fadD15	Lipid metabolism	371.89	320.49	324.04	281.69	309.79	277.09	5.11E-14	1	0.778380237
MMAR_3268	MMAR_3268-1	Lipid metabolism	36.15	34.74	42.6	19.66	19.02	24.96	8.52E-09	0.999999991	0.509261911
MMAR_3271	MMAR_3271-1	Lipid metabolism	37.88	38.29	42.29	32.4	26.74	29.05	6.10E-06	0.999993904	0.67652446
MMAR_3272	MMAR_3272-1	Lipid metabolism	152.04	120.98	119.53	114.87	110.56	98.22	5.18E-05	0.999948152	0.752558774
MMAR_3336	fabD	Lipid metabolism	839.48	962.49	1434.77	342.41	378.57	420.3	0.002672026	0.997327974	0.317445115
MMAR_3338	kasA	Lipid metabolism	2019.85	2081	2819.18	520.61	725.9	917.06	1.69E-08	0.999999983	0.282695717
MMAR_3339	kasB	Lipid metabolism	1654.16	1475.17	1928.7	394.66	551.7	615.62	0	1	0.280109149
MMAR_3340	accD6	Lipid metabolism	849.85	792.64	911.25	200.1	300.49	323.89	0	1	0.293003453
MMAR_3445	cdh	Lipid metabolism	222.37	204.8	170.32	443.13	514.4	407.1	1.11E-16	1	2.075760503
MMAR_3473	MMAR_3473-1	Lipid metabolism	9.31	9.26	9.16	22.91	29.66	14.81	0.036749171	0.963250829	2.198049889
MMAR_3631	MMAR_3631-1	Lipid metabolism	89.33	84.18	97.67	127.51	121.28	130.45	0.00215534	0.99784466	1.270149404
MMAR_3833	plsB2	Lipid metabolism	193.39	180.88	193.99	264.61	254.91	255.87	2.99E-12	1	1.240773995
MMAR_3834	plsC	Lipid metabolism	479.06	535.93	498.32	756.77	720.38	766.77	1.41E-08	0.999999986	1.344985772
MMAR_3962	fas	Lipid metabolism	1592.85	1641.7	1884.21	404.97	574.28	602.05	0	1	0.280237097
MMAR_4250	ltp2	Lipid metabolism	10.9	6.71	11.31	21.88	23.83	35.95	0.003300224	0.996699776	2.575153337
MMAR_4300	omt_2	Lipid metabolism	96.15	61.39	63.14	115.58	115.74	106.93	0.040038195	0.959961805	1.404195302
MMAR_4317	echA1_1	Lipid metabolism	64.61	51.65	75.65	17.74	23.93	21.32	6.79E-14	1	0.297497722
MMAR_4318	fadA6_3	Lipid metabolism	158.51	144.7	204.65	69.79	74.84	75.34	2.00E-08	0.99999998	0.392292499
MMAR_4355	ispH	Lipid metabolism	232.25	204.94	240.43	185.65	187.73	202.62	5.60E-05	0.999944027	0.771966914
MMAR_4393	fadA3	Lipid metabolism	548.16	505.86	453.26	652.94	742.93	682.66	2.55E-06	0.999997451	1.254783981
MMAR_4534	accA2	Lipid metabolism	41.03	50.91	40.76	27.79	29.71	34.19	0.001043437	0.998956563	0.626184009
MMAR_4547	MMAR_4547-1	Lipid metabolism	12.37	14.36	16.17	7.1	10.6	5.88	0.012961709	0.987038291	0.496362421
MMAR_4676	fadB	Lipid metabolism	750.37	855.37	796.94	446.38	551.19	507.86	7.88E-10	0.999999999	0.567900473
MMAR_5001	fadD19_1	Lipid metabolism	49	43.79	51.31	31.6	34.84	44.12	0.003500551	0.996499449	0.697454089
MMAR_5049	fadE30	Lipid metabolism	56.12	68.54	68.14	38.17	45.83	46.73	0.002017087	0.997982913	0.612574909
MMAR_5236	MMAR_5236-1	Lipid metabolism	307.96	306.82	310.21	392.76	443.29	450.01	1.68E-05	0.999983173	1.261831492
MMAR_5363	accD4_1	Lipid metabolism	604.34	567.57	600.1	305.82	381.46	373.91	0	1	0.544048166
MMAR_5364	pks13	Lipid metabolism	486.51	556.54	710.07	208.09	251.37	277.25	4.42E-05	0.999955775	0.380121556
MMAR_5365	fadD32	Lipid metabolism	792.33	911.2	1310.95	333.93	377.96	391.39	0.001674314	0.998325686	0.330310935

PE/PPE -14 genes

GENE ID	GENE NAME	FUNCTION	FPKM Fragments Per Kilobase of transcript per Million mapped reads.						PPEE	PPDE	RealFC
			NORMAL Short			LSMMG Short			posterior probability that a transcript is equally expressed	posterior probability that a transcript is differentially expressed	real fold change is the ratio of the normalized mean count values for LSMMG over the normalized mean count values for normal
			39.5hrs	40hrs	40.5hrs	39.5hrs	40hrs	40.5hrs			
MMAR_0261	MMAR_0261-1	PE/PPE	11.72	15.53	11.56	29.87	25.75	19.45	0.005539107	0.994460893	1.750200142
MMAR_0641	MMAR_0641-1	PE/PPE	17.32	14.87	14.58	38.46	48.19	41.95	0	1	2.507630281
MMAR_0787	MMAR_0787-1	PE/PPE	757.24	863.3	593.85	1270.18	1490.47	1389.85	3.28E-11	1	1.701469184
MMAR_1639	MMAR_1639-1	PE/PPE	21.63	20.14	18.86	38.75	37.33	35.05	0	1	1.670326303
MMAR_2274	MMAR_2274-1	PE/PPE	24.42	18.48	32.79	9.78	11.72	15.09	0.002305967	0.997694033	0.438608672
MMAR_2671	MMAR_2671-1	PE/PPE	202.97	242.71	265.38	350.8	381.39	374.19	0.001816413	0.998183587	1.405367891
MMAR_2895	MMAR_2895-1	PE/PPE	116.39	131.77	109.46	273.02	278.24	310.07	0	1	2.184053851
MMAR_3661	MMAR_3661-1	PE/PPE	163.65	123.94	116.67	106.47	100.15	99.46	0.000117927	0.999882073	0.692022703
MMAR_3665	MMAR_3665-1	PE/PPE	292.26	338.56	310.11	595.49	533.77	618.94	2.85E-12	1	1.684518379
MMAR_3666	MMAR_3666-1	PE/PPE	418.29	499.06	351.5	825.44	714.99	926.52	2.63E-05	0.999973716	1.76581828
MMAR_4451	MMAR_4451-1	PE/PPE	74.1	79.55	105.97	31.88	29.07	30.6	3.98E-08	0.99999996	0.3182064
MMAR_4452	MMAR_4452-1	PE/PPE	34.7	37.17	52.12	13.3	11.18	10.24	2.40E-08	0.999999976	0.252982104
MMAR_4562	MMAR_4562-1	PE/PPE	37.54	24.63	20.95	62.31	47.87	58.05	0.001397771	0.998602229	1.859820864
MMAR_4899	MMAR_4899-1	PE/PPE	74.81	73.04	47.78	174.85	195	177.23	0	1	2.547585232

Regulatory protein - 36 genes

GENE ID	GENE NAME	FUNCTION	FPKM Fragments Per Kilobase of transcript per Million mapped reads.						PPEE	PPDE	RealFC
			NORMAL Short			LSMMG Short			posterior probability that a transcript is equally expressed	posterior probability that a transcript is differentially expressed	real fold change is the ratio of the normalized mean count values for LSMMG over the normalized mean count values for normal
			39.5hrs	40hrs	40.5hrs	39.5hrs	40hrs	40.5hrs			
MMAR_0036	MMAR_0036-1	Regulatory protein	1087.02	816.24	888.21	1605.63	1861.18	1521.26	3.55E-15	1	1.619256907
MMAR_0042	MMAR_0042-1	Regulatory protein	56.38	33.5	32.6	22.39	25.53	23.97	0.01342522	0.98657478	0.539222428
MMAR_0062	MMAR_0062-1	Regulatory protein	120.11	123.79	141.55	97.29	95.58	81.43	0.00019215	0.99980785	0.642975243
MMAR_0150	MMAR_0150-1	Regulatory protein	28.64	15.64	26.26	37.33	56.8	47.99	0.033782949	0.966217051	1.837353871
MMAR_0381	MMAR_0381-1	Regulatory protein	284.02	335.36	304.73	243.95	223.08	240.75	0.002781143	0.997218857	0.691158749
MMAR_0520	MMAR_0520-1	Regulatory protein	65.77	64.54	52.84	13.59	19.99	11.89	0	1	0.225493891
MMAR_0536	MMAR_0536-1	Regulatory protein	153.52	123.59	110.45	112.51	106.36	86.95	0.021679696	0.978320304	0.718714705
MMAR_0640	hspR	Regulatory protein	184.05	166.56	137.19	363.37	556.52	477.04	1.62E-09	0.999999998	2.587240215
MMAR_0773	MMAR_0773-1	Regulatory protein	89.42	64.17	116	42.48	62.25	45.92	0.033598835	0.966401165	0.50560788
MMAR_0799	MMAR_0799-1	Regulatory protein	1099.12	1869.72	1541.88	658.76	554.77	755.03	0.00998168	0.99001832	0.389384958
MMAR_0815	senX3	Regulatory protein	125.67	158.05	111.47	224.11	213.06	218.68	1.33E-07	0.999999867	1.503664407
MMAR_1132	whiB3	Regulatory protein	973.22	1517.33	2700.9	289.66	315.22	339.22	0.048635603	0.951364397	0.159575269
MMAR_1239	MMAR_1239-1	Regulatory protein	853.04	800.66	693.92	1108.99	1025.5	978.81	0.005694302	0.994305698	1.20429822
MMAR_1312	pvdS	Regulatory protein	488.06	379.2	376.67	572.41	592.65	514.68	0.030746079	0.969253921	1.231207073
MMAR_1337	MMAR_1337-1	Regulatory protein	572.34	654.24	554.78	517.24	477.41	502.73	0.02409095	0.97590905	0.762414034
MMAR_1365	whiB7	Regulatory protein	259.74	202.49	257.94	222.98	137.59	156.09	0.042973978	0.957026022	0.647169195
MMAR_1517	devS	Regulatory protein	18.4	19.27	20.64	25.82	41.09	36.79	0.037594394	0.962405606	1.611172278
MMAR_1630	MMAR_1630-1	Regulatory protein	26.25	22.28	15.24	34.68	38	32.2	0.010342659	0.989657341	1.503673431
MMAR_1725	MMAR_1725-1	Regulatory protein	25.5	11.18	20.52	4.03	7.32	6.13	2.02E-05	0.999979777	0.280380306
MMAR_1788	glnB	Regulatory protein	4771.93	4400.21	3953.98	3462.44	3469.5	3052.3	0	1	0.685084166
MMAR_2281	MMAR_2281-1	Regulatory protein	307.96	350.2	358.9	179.08	166.58	201.64	9.64E-10	0.999999999	0.485341194
MMAR_2286	moxR1	Regulatory protein	487.91	384.58	459.48	379.73	409.63	411.04	0.000497786	0.999502214	0.820469799
MMAR_2655	MMAR_2655-1	Regulatory protein	8.92	20.72	25.37	5	3.31	4.32	0.018135299	0.981864701	0.204580835
MMAR_3423	MMAR_3423-1	Regulatory protein	2435.42	2401.95	3014.17	5033.33	4340.73	4960.1	0.000126107	0.999873893	1.648657283
MMAR_3547	MMAR_3547-1	Regulatory protein	71.27	74.55	78.54	97.21	98.78	99.06	0.011542068	0.988457932	1.193595956
MMAR_3703	MMAR_3703-1	Regulatory protein	105.03	89.25	86.57	199.51	333.79	422.97	0.002792091	0.997207909	3.101124723
MMAR_4156	pknH	Regulatory protein	365.83	339.13	377.72	471.33	475.44	493.97	2.46E-05	0.999975361	1.209306938
MMAR_4242	MMAR_4242-1	Regulatory protein	553.75	503.59	489.82	775.76	766.97	705.2	0	1	1.321764207
MMAR_4857	MMAR_4857-1	Regulatory protein	123.15	121.42	93.48	87.49	77.47	79.42	3.79E-05	0.999962078	0.657994913
MMAR_4865	MMAR_4865-1	Regulatory protein	771.2	654.89	610.16	592.27	560.66	539.82	2.43E-09	0.999999998	0.75687877
MMAR_4941	phoR	Regulatory protein	105.2	99.41	116.2	57.22	69.59	75.43	1.14E-09	0.999999999	0.572032494
MMAR_4942	phoP	Regulatory protein	1107.45	1178.06	1662.37	675.1	748.47	750.34	0.042887131	0.957112869	0.495986165
MMAR_5069	MMAR_5069-1	Regulatory protein	79.71	87.44	97.19	73.3	55.05	57.98	0.011439974	0.988560026	0.635736696
MMAR_5182	MMAR_5182-1	Regulatory protein	281.28	315.52	244.09	485.65	494.82	486.22	0	1	1.581024068
MMAR_5183	MMAR_5183-1	Regulatory protein	128.23	110.12	144.16	309.93	219.18	286.62	0.004521229	0.995478771	1.92640864
MMAR_5343	MMAR_5343-1	Regulatory protein	182.13	168.28	172.22	267.59	360.11	359.91	3.66E-05	0.999963407	1.697818768

Unknown - 3 genes

GENE ID	GENE NAME	FUNCTION	FPKM Fragments Per Kilobase of transcript per Million mapped reads.						PPEE	PPDE	RealFC
			NORMAL Short			LSMMG Short			posterior probability that a transcript is equally expressed	posterior probability that a transcript is differentially expressed	real fold change is the ratio of the normalized mean count values for LSMMG over the normalized mean count values for normal
			39.5hrs	40hrs	40.5hrs	39.5hrs	40hrs	40.5hrs			
MMAR_2858	MMAR_2858-1	unknown	176.59	198.32	244.91	105.19	120.25	83.56	0.00025292	0.99974708	0.444406521
MMAR_3574	MMAR_3574-1	unknown	42.71	46.07	61.41	21.83	23.16	33.8	0.003506801	0.996493199	0.473540355
MMAR_4957	MMAR_4957-1	unknown	584.1	609.85	559.31	479.64	404.98	362.78	1.20E-07	0.99999988	0.641746588

Virulence, adaptation and detoxification - 11 genes

GENE ID	GENE NAME	FUNCTION	FPKM Fragments Per Kilobase of transcript per Million mapped reads.						PPEE	PPDE	RealFC
			NORMAL Short			LSMMG Short			posterior probability that a transcript is equally expressed	posterior probability that a transcript is differentially expressed	real fold change is the ratio of the normalized mean count values for LSMMG over the normalized mean count values for normal
			39.5hrs	40hrs	40.5hrs	39.5hrs	40hrs	40.5hrs			
MMAR_0515	hsp	virulence, adaptation and detoxification	14.92	17.9	17.49	63.57	84.69	168.2	0.018652867	0.981347133	5.67916644
MMAR_0637	dnaK	virulence, adaptation and detoxification	760.45	838.89	753.78	2013.68	3095.42	3932.23	0.000289328	0.999710672	3.485261785
MMAR_0638	grpE	virulence, adaptation and detoxification	184.34	156.59	144.95	318.56	572.55	539.34	0.000219805	0.999780195	2.676184662
MMAR_0639	dnaJ	virulence, adaptation and detoxification	166.28	158.49	183.41	350.7	523.33	563.86	3.33E-06	0.999996665	2.565539823
MMAR_2065	MMAR_2065-1	virulence, adaptation and detoxification	145.2	134.02	168.6	88.81	93.45	93.03	3.67E-08	0.999999963	0.55584684
MMAR_2410	bcpB	virulence, adaptation and detoxification	889.21	861.66	675.24	1335.01	1275.38	1182.21	6.07E-09	0.999999994	1.4151552
MMAR_2499	tlyA	virulence, adaptation and detoxification	244.6	191.63	177.1	165.5	142.32	159.3	0.000103334	0.999896666	0.695482513
MMAR_3521	htpG	virulence, adaptation and detoxification	1534.63	1548.9	1327.13	2077.86	2172.12	2241.59	2.33E-15	1	1.338620949
MMAR_3527	MMAR_3527-1	virulence, adaptation and detoxification	1004.32	809.28	922.11	1483.57	1469.61	1493.49	0.042696379	0.957303621	1.464663125
MMAR_3683	dnaJ2	virulence, adaptation and detoxification	181.33	119.51	129.5	110.96	111.31	103.31	0.00515766	0.99484234	0.69253669
MMAR_4051	mce3A_1	virulence, adaptation and detoxification	45.67	44.51	61.96	80.09	88	92.65	0.001492998	0.998507002	1.551352155
