# Supplementary material for: Exposure of Mycobacterium marinum to low-shear modeled microgravity: effect on growth, the transcriptome and survival under stress
Source: NPJ Microgravity. 2016 Dec 1;2:16038–. doi: 10.1038/npjmgrav.2016.38 (PMC5515531; doi:10.1038/npjmgrav.2016.38)
Supplement: Supplementary Excel Worksheet 2 [file npjmgrav201638-s4.pdf]

Cell wall and cell processes - 54 genes

GENE ID	GENE NAME	FUNCTION	FPKM Fragments Per Kilobase of transcript per Million mapped reads. NORMAL Long			LSMMG Long			PPEE posterior probability that a transcript is equally expressed	PPDE posterior probability that a transcript is differentially expressed	RealFC real fold change is the ratio of the normalized mean count values for LSMMG over the normalized mean count values for normal
			4days 35hr	4days 36hr	4days 37hr	4days 35hr	4days 36hr	4days 37hr			
MMAR_0142	ltp1_1	cell wall and cell processes	61.57	57.72	49.4	125.2	112.16	96.59	6.86E-08	0.999999931	1.846561413
MMAR_0218	MMAR_0218-1	cell wall and cell processes	3.08	0.85	1.99	14.86	17.99	25.46	2.21E-12	1	9.481240366
MMAR_0446	mmpL3	cell wall and cell processes	740.49	594.46	641.03	469.74	442.17	495.85	8.08E-08	0.999999919	0.671566534
MMAR_0493	MMAR_0493-1	cell wall and cell processes	60.28	49.81	69.01	107.5	99.84	87.68	7.62E-07	0.999999238	1.558340356
MMAR_0495	MMAR_0495-1	cell wall and cell processes	992.25	856.25	925.39	1228.72	1236.12	1369.04	1.03E-06	0.999998972	1.286338061
MMAR_0502	MMAR_0502-1	cell wall and cell processes	318.82	242.5	238.19	404.86	397.66	407.27	3.46E-05	0.99996536	1.416585623
MMAR_0543	MMAR_0543-1	cell wall and cell processes	238.69	232.63	250.24	302.89	301.78	342.8	0.001819101	0.998180899	1.235130266
MMAR_0546	esxG	cell wall and cell processes	1782.91	1266.45	2178.41	1345.72	1153.32	1028.92	0.011496909	0.988503091	0.623311456
MMAR_0547	esxH	cell wall and cell processes	1313.97	1048.45	1390.83	869.01	866.63	675.78	0	1	0.589678948
MMAR_0548	MMAR_0548-1	cell wall and cell processes	667.37	516.46	702.43	464.92	380.42	364.4	1.11E-16	1	0.604111523
MMAR_0549	MMAR_0549-1	cell wall and cell processes	78.5	68.91	101.48	71.36	40.21	55.56	0.030723673	0.969276327	0.632459205
MMAR_0963	MMAR_0963-1	cell wall and cell processes	316.13	272.87	287.53	384.35	352.51	372.33	0.007007298	0.992992702	1.183203286
MMAR_1005	mmpL5	cell wall and cell processes	1230.29	1597.7	1059.04	214.71	325.85	380.07	0.000320702	0.999679298	0.219623297
MMAR_1006	mmpS5	cell wall and cell processes	1275.11	1301.54	1069.72	152.06	172.9	242.16	0	1	0.142836513
MMAR_1270	MMAR_1270-1	cell wall and cell processes	83.83	81.65	106.54	158.62	118.35	166.28	0.005124421	0.994875579	1.52348727
MMAR_1271	ctpC	cell wall and cell processes	92.83	83.55	108.86	226.33	208.62	276.96	1.11E-16	1	2.355513724
MMAR_1345	MMAR_1345-1	cell wall and cell processes	103.76	86.56	120.85	89.04	67.1	74.69	3.07E-05	0.999969324	0.699519504
MMAR_1554	MMAR_1554-1	cell wall and cell processes	179.46	204.07	223.41	346.36	363.37	287.25	0.003278744	0.996721256	1.538551259
MMAR_1754	MMAR_1754-1	cell wall and cell processes	39.81	26.15	27.52	90.07	90.91	156.3	0.000150372	0.999849628	3.394238836
MMAR_1769	drrC	cell wall and cell processes	676.72	593.26	585.2	491.56	498.55	436.83	0.024444634	0.975555366	0.718778206
MMAR_1771	drrA	cell wall and cell processes	800.72	746.4	804.34	610.01	619.81	554.01	3.76E-11	1	0.711027417
MMAR_1840	MMAR_1840-1	cell wall and cell processes	255.24	240.67	311.85	376.54	348.11	342.85	0.010192428	0.989807572	1.246945965
MMAR_1887	efpA	cell wall and cell processes	357.22	406.34	648.38	172.99	224.02	220.09	0.020765539	0.979234461	0.414706013
MMAR_1967	ftsK	cell wall and cell processes	607.73	519.03	609.02	705.04	739.33	730.56	3.50E-07	0.99999965	1.183531373
MMAR_2004	MMAR_2004-1	cell wall and cell processes	650.2	421.27	570.24	391.52	376.33	367.78	4.09E-05	0.999959064	0.64410197
MMAR_2026	MMAR_2026-1	cell wall and cell processes	73.47	68	64.4	140.94	108.34	104.81	0.000259706	0.999740294	1.601908366
MMAR_2268	MMAR_2268-1	cell wall and cell processes	906.98	848.47	824.67	481.5	454.84	454.7	7.77E-16	1	0.502434773
MMAR_2389	MMAR_2389-1	cell wall and cell processes	366.78	607.76	562.62	208.04	234.25	235.48	0.009392428	0.990607572	0.409357196
MMAR_2424	cydD	cell wall and cell processes	30.66	19.51	27.93	59.66	52.13	42.8	1.07E-05	0.999989332	1.88022767
MMAR_2426	cydA	cell wall and cell processes	352.44	368.06	351.45	498.11	689.1	612.34	0.031809756	0.968190244	1.573927743
MMAR_2439	MMAR_2439-1	cell wall and cell processes	31.89	30.15	39.64	29.11	20.64	20.17	0.000957598	0.999042402	0.647570198
MMAR_2502	MMAR_2502-1	cell wall and cell processes	552.92	567.28	636.49	1000.2	1072.28	954.04	7.35E-14	1	1.617017148
MMAR_2716	MMAR_2716-1	cell wall and cell processes	71.39	50.75	81.83	53.34	52.29	54.84	0.049525066	0.950474934	0.74800785
MMAR_2772	MMAR_2772-1	cell wall and cell processes	562.15	491.75	708.77	1951.39	1849.68	1666.22	0	1	2.88611859
MMAR_2929	MMAR_2929-1	cell wall and cell processes	248.07	297	455.08	712.01	643.95	538.75	0.038755355	0.961244645	1.749117138
MMAR_3234	MMAR_3234-1	cell wall and cell processes	525.4	417.64	524.36	465.74	393.88	444.46	1.98E-06	0.999998023	0.836919994
MMAR_3250	MMAR_3250-1	cell wall and cell processes	334.45	337.63	435.53	255.04	266.47	284.95	3.37E-05	0.999966305	0.682175072
MMAR_3359	MMAR_3359-1	cell wall and cell processes	78.7	59.39	63.88	122.07	109.43	100.5	3.92E-07	0.999999608	1.544754947
MMAR_3364	MMAR_3364-1	cell wall and cell processes	3.42	2.84	4.42	8.48	10.48	15.34	0.001398909	0.998601091	3.042674622
MMAR_3554	MMAR_3554-1	cell wall and cell processes	367.17	447.88	240.47	34.28	50.77	35.52	2.60E-05	0.999973956	0.105693061
MMAR_3568	MMAR_3568-1	cell wall and cell processes	49.71	62.92	38.69	140.9	156.18	87.81	0.013028723	0.986971277	2.347166685
MMAR_3653	esxN_3	cell wall and cell processes	800.02	958.65	1360.09	692.4	601.84	589.17	0.016376353	0.983623647	0.554080951
MMAR_3658	MMAR_3658-1	cell wall and cell processes	100.99	77.94	99.64	1129.35	974.27	602.57	1.23E-12	1	9.136067243
MMAR_3698	mbtA	cell wall and cell processes	25.85	19.44	21.8	50.37	49.54	55.63	2.17E-11	1	2.185912491
MMAR_3744	mmpL4_1	cell wall and cell processes	80.61	85.46	56.76	146.77	240.05	253.27	0.000487165	0.999512835	2.677489519
MMAR_4138	oppD	cell wall and cell processes	96.69	92.4	103.23	158.59	150.76	129.81	4.55E-05	0.999954475	1.413083161
MMAR_4182	MMAR_4182-1	cell wall and cell processes	11.93	7.54	9.6	21.68	17.34	28.04	0.000124517	0.999875483	2.176633996
MMAR_4576	pstS2	cell wall and cell processes	77.02	78.62	88.63	166.37	128.09	118.21	0.003817366	0.996182634	1.580791837
MMAR_4580	phoS2	cell wall and cell processes	433.4	370.59	482.39	555.17	535.61	606.18	1.28E-05	0.999987191	1.243089578
MMAR_4623	MMAR_4623-1	cell wall and cell processes	458.44	420.15	432.42	723.82	672.06	555.61	0.030735112	0.969264888	1.397358042
MMAR_5101	lsr2	cell wall and cell processes	3192.73	2662.41	3408.91	1894.27	1644.54	2197.14	0	1	0.57142495
MMAR_5368	fbpA	cell wall and cell processes	6270.93	5164.38	5902.18	3446.02	3507.05	4740.34	2.01E-05	0.999979899	0.633374103
MMAR_5369	MMAR_5369-1	cell wall and cell processes	306.85	238.27	300	237.1	194.64	207.96	3.33E-16	1	0.714820219
MMAR_5474	MMAR_5474-1	cell wall and cell processes	135.27	116.51	150.82	121.69	121.65	126.63	0.001073818	0.998926182	0.86994504

Conserved hypotheticals - 64 genes

GENE ID	GENE NAME	FUNCTION	FPKM Fragments Per Kilobase of transcript per Million mapped reads. NORMAL Long			LSMMG Long			PPEE posterior probability that a transcript is equally expressed	PPDE posterior probability that a transcript is differentially expressed	RealFC real fold change is the ratio of the normalized mean count values for LSMMG over the normalized mean count values for normal
			4days 35hr	4days 36hr	4days 37hr	4days 35hr	4days 36hr	4days 37hr			
MMAR_0022	MMAR_0022-1	conserved hypotheticals	402.44	277.06	367.6	269.55	221.33	296.11	0.003395242	0.996604758	0.710831655
MMAR_0066	MMAR_0066-1	conserved hypotheticals	162.9	116.9	187.55	59.72	73.42	85.22	2.06E-10	1	0.440140731
MMAR_0119	MMAR_0119-1	conserved hypotheticals	11.24	14.18	8.29	22.62	20.47	18.97	0.038858181	0.961141819	1.692982422
MMAR_0120	MMAR_0120-1	conserved hypotheticals	13.88	20.12	20.99	38.53	34.96	47.5	1.04E-06	0.999998958	2.047772137
MMAR_0137	MMAR_0137-1	conserved hypotheticals	365.04	384.64	368.05	229.64	313.52	220.24	0.011023625	0.988976375	0.620391814
MMAR_0232	MMAR_0232-1	conserved hypotheticals	5.51	10.03	4.29	38.68	43.18	120.39	0.021674017	0.978325983	9.201334139
MMAR_0348	MMAR_0348-1	conserved hypotheticals	86.37	113.67	116.36	165.47	162.91	215.67	0.015597188	0.984402812	1.586854609
MMAR_0398	MMAR_0398-1	conserved hypotheticals	28.72	34.3	24.23	44.14	50.8	63.15	0.03455008	0.96544992	1.668774143
MMAR_0425	MMAR_0425-1	conserved hypotheticals	42.73	30.96	34.87	60.18	45.16	52.87	0.024209051	0.975790949	1.371362136
MMAR_0447	MMAR_0447-1	conserved hypotheticals	193.81	152.14	204.83	130.64	104.62	143.58	3.07E-09	0.999999997	0.64567681
MMAR_0519	MMAR_0519-1	conserved hypotheticals	18.43	39.13	34.73	11.39	10.38	13.22	0.027084601	0.972915399	0.348392093
MMAR_0523	MMAR_0523-1	conserved hypotheticals	21.28	5.51	13.67	61.83	59.81	74.65	0	1	4.667340235
MMAR_0524	MMAR_0524-1	conserved hypotheticals	4.93	6.74	7.86	56.71	46.03	62.4	0	1	7.842456177
MMAR_0853	MMAR_0853-1	conserved hypotheticals	135.2	72.84	102.73	869.42	948.96	1150.12	0	1	8.945263929
MMAR_0905	MMAR_0905-1	conserved hypotheticals	17.8	34.92	36.35	56.27	54.57	60.85	0.002408374	0.997591626	1.788480593
MMAR_0982	MMAR_0982-1	conserved hypotheticals	266.02	256.66	355.18	130.85	173.59	168.17	3.88E-08	0.999999961	0.509518816
MMAR_1007	MMAR_1007-1	conserved hypotheticals	996.79	1050.56	1387.64	184.62	189.41	320.64	0	1	0.188325345
MMAR_1241	MMAR_1241-1	conserved hypotheticals	6.32	1.75	6.38	14.36	12.44	20.13	0.000114528	0.999885472	3.147936882
MMAR_1272	MMAR_1272-1	conserved hypotheticals	222.53	257.64	223.22	406.14	534.05	507.06	3.17E-06	0.999996833	1.86882579
MMAR_1304	MMAR_1304-1	conserved hypotheticals	434.56	466.35	532.68	671.5	716.36	694.2	8.76E-06	0.999991245	1.355602947
MMAR_1572	MMAR_1572-1	conserved hypotheticals	264.48	289.47	278.76	396.13	409.11	386.85	0.000297597	0.999702403	1.333689
MMAR_1588	MMAR_1588-1	conserved hypotheticals	48.29	40.07	43.83	73.25	68.4	57.02	0.032574389	0.967425611	1.411853751
MMAR_1915	MMAR_1915-1	conserved hypotheticals	298.97	237.24	264.1	238.42	194	214.9	0.001997949	0.998002051	0.758425688
MMAR_2009	MMAR_2009-1	conserved hypotheticals	39.46	38.99	46.31	106.42	123.4	85.88	7.00E-07	0.9999993	2.348411687
MMAR_2503	MMAR_2503-1	conserved hypotheticals	255.37	289.44	350	495.22	439.53	439.56	0.000167025	0.999832975	1.437610319
MMAR_2771	MMAR_2771-1	conserved hypotheticals	142.67	135.63	135.1	324.7	333.91	308.36	1.18E-12	1	2.158940953
MMAR_2779	MMAR_2779-1	conserved hypotheticals	424.69	379.91	390.29	317.59	307.8	310.81	6.96E-06	0.999993044	0.734204379
MMAR_2935	MMAR_2935-1	conserved hypotheticals	14.98	14.05	18.99	42.44	43.56	56.22	8.66E-15	1	2.792369002
MMAR_2946	MMAR_2946-1	conserved hypotheticals	39.26	40.31	30.93	125.82	81.07	123.48	4.43E-09	0.999999996	2.735189633
MMAR_3007	MMAR_3007-1	conserved hypotheticals	232.48	216.43	265.45	437.05	419.38	383.66	0	1	1.62859639
MMAR_3010	MMAR_3010-1	conserved hypotheticals	66.16	56.89	62.58	139.43	129.33	109	9.99E-13	1	1.908789192
MMAR_3068	MMAR_3068-1	conserved hypotheticals	87.61	92.43	89.34	140.83	143.71	121.07	0.002722575	0.997277425	1.412521268
MMAR_3070	MMAR_3070-1	conserved hypotheticals	106.1	96.84	92.11	189.63	207.5	168.6	3.48E-09	0.999999997	1.798703576
MMAR_3088	MMAR_3088-1	conserved hypotheticals	137.77	175.01	168.99	246.04	279.89	252.77	0.000879514	0.999120486	1.507978076
MMAR_3187	MMAR_3187-1	conserved hypotheticals	1884.65	1698.93	2051.86	3146.72	2432.2	2622.44	0.000534013	0.999465987	1.358888335
MMAR_3207	MMAR_3207-1	conserved hypotheticals	364.08	357.36	312.85	462.93	520.8	500.69	0.037304308	0.962695692	1.337159061
MMAR_3365	MMAR_3365-1	conserved hypotheticals	77.7	72.39	50.89	123.5	118.42	97.87	0.040374194	0.959625806	1.557066121
MMAR_3430	MMAR_3430-1	conserved hypotheticals	50.77	45.15	37.2	68.86	72.24	87.96	0.000950398	0.999049602	1.603135779
MMAR_3549	MMAR_3549-1	conserved hypotheticals	26.06	29.9	30.65	8.34	6.04	12.67	1.11E-16	1	0.291160883
MMAR_4142	MMAR_4142-1	conserved hypotheticals	88.1	83.68	100.81	132.69	140.23	154.46	1.01E-10	1	1.479029336
MMAR_4168	MMAR_4168-1	conserved hypotheticals	109.54	120.32	142.59	252.78	245.47	213.48	8.08E-12	1	1.78330456
MMAR_4248	MMAR_4248-1	conserved hypotheticals	18.95	30.01	23.94	75.09	48.25	50.98	0.018595722	0.981404278	2.172606126
MMAR_4249	MMAR_4249-1	conserved hypotheticals	20.64	10.19	19.72	37.37	58.16	38.98	0.016581089	0.983418911	2.517745722
MMAR_4254	MMAR_4254-1	conserved hypotheticals	16.72	8.41	20.26	29.83	31.21	41.07	0.000978657	0.999021343	2.164431082
MMAR_4275	MMAR_4275-1	conserved hypotheticals	310.05	373.21	429.09	268.13	292.45	256.77	0.016748746	0.983251254	0.683285287
MMAR_4306	MMAR_4306-1	conserved hypotheticals	87668.97	50568.35	34812.06	380806.3	356581.2	242741.47	0	1	4.91127938
MMAR_4356	MMAR_4356-1	conserved hypotheticals	979.94	1023.48	1332.23	681.55	887.98	834.08	0.020737167	0.979262833	0.675052185
MMAR_4413	MMAR_4413-1	conserved hypotheticals	21.73	26.16	38.23	62.4	81.41	74.59	1.18E-07	0.999999882	2.396180862
MMAR_4498	MMAR_4498-1	conserved hypotheticals	537.26	397.85	600.61	2432.03	2858.04	3239.66	0	1	5.104761732
MMAR_4609	MMAR_4609-1	conserved hypotheticals	450.56	661.18	570.32	1698.62	1685.85	1088.99	0.000267377	0.999732623	2.440657248
MMAR_4645	MMAR_4645-1	conserved hypotheticals	631.58	735.78	650.96	1037.05	1084.26	1220.16	3.36E-05	0.999966366	1.52205435
MMAR_4647	MMAR_4647-1	conserved hypotheticals	296.11	312.24	367.5	519.49	597.24	483.61	0.000851696	0.999148304	1.54033456
MMAR_4761	MMAR_4761-1	conserved hypotheticals	43.14	47.98	53.28	81.79	108.96	103.23	1.78E-06	0.999998215	1.91074954
MMAR_4772	MMAR_4772-1	conserved hypotheticals	30.3	24.67	26.42	42.76	42.25	64.68	0.038210244	0.961789756	1.723354945
MMAR_4858	MMAR_4858-1	conserved hypotheticals	131.32	85.53	116.34	91.37	70.39	87.96	0.010654358	0.989345642	0.71023253
MMAR_4903	MMAR_4903-1	conserved hypotheticals	23.72	21.27	15.45	52.16	60.17	120.2	0.037038441	0.962961559	3.570269446
MMAR_4959	MMAR_4959-1	conserved hypotheticals	2160.67	2472.07	3573.95	4997.68	5468.39	4739.61	0.000489384	0.999510616	1.698848926
MMAR_5103	MMAR_5103-1	conserved hypotheticals	495.52	486.78	585.72	214.3	232.33	255.58	0	1	0.419771546
MMAR_5106	MMAR_5106-1	conserved hypotheticals	125.98	75.75	130.13	64.13	51.87	85.3	0.015180175	0.984819825	0.575325861
MMAR_5340	MMAR_5340-1	conserved hypotheticals	422.06	295.73	397.26	317.26	285.25	327.62	0.002846301	0.997153699	0.789141301
MMAR_5437	MMAR_5437-1	conserved hypotheticals	239.48	164.07	327.43	506.92	506.04	393.72	0.015827518	0.984172482	1.783782079
MMAR_5455	MMAR_5455-1	conserved hypotheticals	196.94	232.42	212.73	391.98	347.42	300.63	0.011653106	0.988346894	1.511471113
MMAR_5457	espB	conserved hypotheticals	462.65	484.94	536.03	757.66	713.03	673.18	1.16E-06	0.999998844	1.354786505
MMAR_5578	MMAR_5578-1	conserved hypotheticals	1932.04	1683.92	1603.49	1400.21	1248.92	1235.3	0.018373998	0.981626002	0.6790101

Information pathways - 49 genes

GENE ID	GENE NAME	FUNCTION	FPKM Fragments Per Kilobase of transcript per Million mapped reads. NORMAL Long 4days 35hr	4days 36hr	4days 37hr	LSMMG Long 4days 35hr	4days 36hr	4days 37hr	PPEE posterior probability that a transcript is equally expressed	PPDE posterior probability that a transcript is differentially expressed	RealFC real fold change is the ratio of the normalized mean count values for LSMMG over the normalized mean count values for normal
MMAR_0072	rpsF	information pathways	1171.81	958.47	873.47	680.27	587.62	695.36	0.049184346	0.950815654	0.595072531
MMAR_0073	ssb	information pathways	1930.78	2393.48	2190.63	1431.88	1596.45	1327.46	0.030875475	0.969124525	0.617030489
MMAR_0074	rpsR1	information pathways	1988.72	1498.71	1574.39	1026.09	1072.76	1069.74	8.78E-05	0.999912161	0.569324291
MMAR_0972	nusG	information pathways	1549.12	1531.28	1569.76	1151.99	1255.26	1303.34	0.000893449	0.999106551	0.743862644
MMAR_0974	rplA	information pathways	2139.34	1648.12	1997.42	1182.04	1311.14	1531.62	4.49E-08	0.999999955	0.653032306
MMAR_0975	MMAR_0975-1	information pathways	83.77	55.08	59.07	40.38	34.13	36.14	0.001717921	0.998282079	0.526538843
MMAR_0990	rplJ	information pathways	1812.82	1488.46	1752.78	970.46	977.79	1156.79	0	1	0.574294408
MMAR_0996	rpoC	information pathways	2262.43	2003.14	2112.18	1545.04	1706.01	1823.03	0.008875707	0.991124293	0.7498948
MMAR_1012	rpsG	information pathways	1959.72	1755.97	2403.87	1475.98	1364.46	1806.9	0.008189108	0.991810892	0.707634337
MMAR_1031	rplC	information pathways	4179.97	3120.82	4122.36	1971.62	2051.01	2696.71	1.54E-12	1	0.552352235
MMAR_1032	rplD	information pathways	3478.87	3285.67	3496.05	2070.38	2108.44	2315.41	0	1	0.590086874
MMAR_1034	rplB	information pathways	3084.07	2508.48	2775.73	1790.7	1747.19	2045.37	2.08E-13	1	0.625257853
MMAR_1036	rplV	information pathways	827.53	527.57	843.95	445.54	416.85	603.87	0.026858404	0.973141596	0.626955379
MMAR_1039	rpmC	information pathways	6878.57	5472.75	5624.71	4298.55	4163.37	4145.9	0.000845522	0.999154478	0.635502394
MMAR_1045	rplN	information pathways	2385.59	2276.86	2547.88	1766.07	1444.76	1676.43	0	1	0.623691855
MMAR_1048	rpsN	information pathways	11883.41	11742.89	11744.89	7407.08	8901.35	8218.61	8.74E-09	0.999999991	0.622348378
MMAR_1052	rpsE	information pathways	1276.37	1054.39	1454.81	738.48	762.45	1043.03	0.0001013	0.9998987	0.631194013
MMAR_1053	rpmD	information pathways	4702.47	4310.74	5223.47	3245.51	3205.49	4167.28	0.000454073	0.999545927	0.678193567
MMAR_1054	rplO	information pathways	3783.43	2802.81	3677.87	2538.22	2403.73	3114.57	0.010871182	0.989128818	0.730851457
MMAR_1085	infA	information pathways	10464.68	10970.77	13649.37	7797.94	8511.49	9031.56	3.48E-06	0.999996519	0.656665658
MMAR_1086	rpmJ	information pathways	4497.08	6141.56	5360.01	2532.38	2820.83	3741.77	0.028208217	0.971791783	0.512280668
MMAR_1089	rpsD	information pathways	3894.96	3301.06	3165.25	2274.24	2365.64	2527.04	0.011908933	0.988091067	0.643337383
MMAR_1090	rpoA	information pathways	2679.13	2197.72	2698.07	1529.53	1431.79	1779.22	0	1	0.588648599
MMAR_1091	rplQ	information pathways	1834.17	1439.17	1920.4	1032.77	896.4	1228.78	1.11E-16	1	0.568764968
MMAR_1106	rplM	information pathways	6944.33	6779.29	6007.38	4058.25	4712.1	4132.67	0.01056793	0.98943207	0.602653796
MMAR_1107	rpsI	information pathways	1794.31	1431.37	1429.78	1005.12	998.23	1031.59	0.000166834	0.999833166	0.603646388
MMAR_1334	sigH	information pathways	1048.48	1169.32	1044.65	1675.16	1809.3	1872.92	1.15E-06	0.999998846	1.524356685
MMAR_1640	nrdH	information pathways	2346.28	2386.84	1711.27	4456.24	4803.78	4654.27	3.17E-11	1	1.940008677
MMAR_1641	nrdI	information pathways	542.65	467.16	454.01	998.01	983.93	912.13	0	1	1.82815651
MMAR_1642	nrdE	information pathways	1060.68	1219.41	1011.56	2370.32	2965.18	2443.75	3.62E-09	0.999999996	2.207293017
MMAR_1647	nrdF2	information pathways	504.26	568.49	489.07	1138.09	1403.56	1256.05	1.82E-14	1	2.263339822
MMAR_1728	hupB	information pathways	3742.58	4039.4	5272.94	2901.38	3053.59	3222.13	0.010088208	0.989911792	0.657876101
MMAR_1742	recG	information pathways	96.18	78.48	97.44	133.39	111.34	114.18	0.000430964	0.999569036	1.245305358
MMAR_1799	rpsP	information pathways	1662.46	1301.98	1455.22	1090.4	904.4	1130.47	1.33E-06	0.999998668	0.656509898
MMAR_1801	rimM	information pathways	805.18	689.49	696.92	476.37	429.89	465.24	1.47E-10	1	0.580449705
MMAR_1802	trmD	information pathways	197.09	112.68	165.94	121.72	89.27	107.95	0.02972639	0.97027361	0.631968784
MMAR_1820	tsf	information pathways	1718.48	1392.49	1611.78	1051.19	1019.95	1237.36	1.08E-13	1	0.656895878
MMAR_1925	gpsI	information pathways	1490.03	1339.51	1498.18	1141.01	1122.43	1116.63	0	1	0.735698974
MMAR_2170	alaS	information pathways	296.61	234.53	295.13	277.5	243.45	249.49	0.004479378	0.995520622	0.881804317
MMAR_2433	rpsA	information pathways	5666.52	6107.7	6100.43	4135.9	4516.8	4589.24	0.005883392	0.994116608	0.693354891
MMAR_2451	tsnR	information pathways	336.42	236	296.12	231.82	168.53	160.75	2.76E-05	0.999972442	0.607046723
MMAR_3733	rpsT	information pathways	1092.9	711.87	960.83	712.55	480.33	597	0.000929874	0.999070126	0.591226474
MMAR_4105	argS	information pathways	315.28	231.06	281.12	230.22	235.05	233.58	0.003165317	0.996834683	0.79875094
MMAR_4143	sbcD	information pathways	86.09	69.59	107.13	122.64	133.58	139.75	0.006098777	0.993901223	1.42840754
MMAR_4216	sigE	information pathways	652.79	638.33	648.08	984.96	909.78	969.51	0	1	1.376251917
MMAR_4289	typA	information pathways	361.47	281.76	300.21	200.14	203.42	205.27	2.55E-08	0.999999975	0.608296884
MMAR_4472	rplY	information pathways	1098.81	999.42	1155.98	684.04	700.16	839.35	0	1	0.638091609
MMAR_5240	dnaZX	information pathways	96.6	84.32	99.8	68.8	73.06	84.68	5.96E-05	0.999940405	0.761004966
MMAR_5569	rnpA	information pathways	784.26	527.62	828.86	438.08	416.29	467.22	1.36E-07	0.999999864	0.574508241

Intermediary metabolism - 96 genes

GENE ID	GENE NAME	FUNCTION	FPKM Fragments Per Kilobase of transcript per Million mapped reads. NORMAL Long			LSMMG Long			PPEE posterior probability that a transcript is equally expressed	PPDE posterior probability that a transcript is differentially expressed	RealFC real fold change is the ratio of the normalized mean count values for LSMMG over the normalized mean count values for normal
			4days 35hr	4days 36hr	4days 37hr	4days 35hr	4days 36hr	4days 37hr			
MMAR_0065	ino1	intermediary metabolism, and respiration	793.25	874.44	1035.72	427.81	473.62	636.12	1.06E-05	0.999989427	0.533198784
MMAR_0107	celA	intermediary metabolism, and respiration	248.81	188.13	266.7	98.96	108.51	139.65		1	0.466519159
MMAR_0324	pepA	intermediary metabolism, and respiration	292.55	257.77	320.24	230.46	205.46	276.88	0.010706665	0.989293335	0.769378128
MMAR_0346	cyp138A3	intermediary metabolism, and respiration	68.44	79.05	58.38	144.16	172.14	251.17	0.001068413	0.998931587	2.554849194
MMAR_0379	pntB	intermediary metabolism, and respiration	410.06	291.57	406.19	308.52	264.76	317.35	0.00025724	0.99974276	0.760828744
MMAR_0516	nirB	intermediary metabolism, and respiration	9.81	11.25	14	4.6	6.77	6.52	1.24E-05	0.999987552	0.482279402
MMAR_0550	mycP3	intermediary metabolism, and respiration	82.09	50.96	79.76	58.42	30.32	41.22	0.011010594	0.988989406	0.57791869
MMAR_0728	lpqM	intermediary metabolism, and respiration	206.78	160.13	194.51	246.41	240.13	225.42	0.001564068	0.998435932	1.197565138
MMAR_0780	putA_1	intermediary metabolism, and respiration	155.52	160.12	220.1	314.52	322.76	274.08	0.000443978	0.999556022	1.606868763
MMAR_0792	icl	intermediary metabolism, and respiration	1292.19	2676.46	2941.96	146.37	273.67	307.4	9.95E-07	0.999999005	0.098247332
MMAR_0829	galE2	intermediary metabolism, and respiration	366.74	264.62	325.03	248.89	197.29	228.48	6.42E-08	0.999999936	0.664373346
MMAR_0842	hemA	intermediary metabolism, and respiration	229.37	161.69	219.19	186.4	132.9	160.04	0.014710927	0.985289073	0.741911775
MMAR_0843	hemC	intermediary metabolism, and respiration	1805.01	1248.99	1989.72	775.71	859.8	904.76	1.10E-11	1	0.477966788
MMAR_0844	hemD	intermediary metabolism, and respiration	472.18	368	497.66	262.76	227.58	274.51	0	1	0.540492608
MMAR_0845	hemB	intermediary metabolism, and respiration	438.89	320.73	388.24	327.53	234.1	254.08	0.000239692	0.999760308	0.667771102
MMAR_0913	sulP	intermediary metabolism, and respiration	155.07	114.45	115.04	260.86	190.04	209.53	0.000319559	0.999680441	1.612432869
MMAR_0914	sulP_1	intermediary metabolism, and respiration	65.83	69.46	71.1	119.4	109.14	102.62	7.42E-10	0.999999999	1.501932721
MMAR_1041	atsA	intermediary metabolism, and respiration	319.85	271.94	274.47	388.41	409.02	397.08	0.000260468	0.999739532	1.297958252
MMAR_1055	MMAR_1055-1	intermediary metabolism, and respiration	166.66	104.4	137.57	94.6	72.76	107.04	0.022600238	0.977399762	0.633034448
MMAR_1079	mmsA	intermediary metabolism, and respiration	228.82	253.17	239.94	370.89	339.62	332.42	0.000364158	0.999635842	1.348403794
MMAR_1108	mrsA	intermediary metabolism, and respiration	248.37	202.18	280.97	155.09	151.23	169.47	0	1	0.615004602
MMAR_1183	MMAR_1183-1	intermediary metabolism, and respiration	433.54	327.26	516.54	810.24	853.24	947.72	4.77E-15	1	1.931542395
MMAR_1184	metA	intermediary metabolism, and respiration	495.21	261.99	392.66	925.54	854.47	1042.09	0	1	2.33257389
MMAR_1185	metC	intermediary metabolism, and respiration	742.19	642.33	693.71	1540.69	2480.12	2601.41	3.23E-05	0.999967697	3.004138688
MMAR_1200	sdhB	intermediary metabolism, and respiration	407.5	394.58	472.96	350.3	386.38	369.36	0.00244864	0.99755136	0.813536653
MMAR_1202	sdhD	intermediary metabolism, and respiration	302.31	208.49	288.71	174.36	167.3	234.19	0.0159748	0.9840252	0.672900969
MMAR_1216	MMAR_1216-1	intermediary metabolism, and respiration	152.84	147.69	151.34	206.93	220.35	198.82	0.000722519	0.999277481	1.295542056
MMAR_1240	pcd	intermediary metabolism, and respiration	65.88	50.4	47.45	114.69	78.03	105.3	0.00019547	0.99980453	1.70348865
MMAR_1243	lat	intermediary metabolism, and respiration	50.57	21.77	26.18	137.54	151.71	230.76	4.57E-09	0.999999995	5.019373579
MMAR_1251	accA3	intermediary metabolism, and respiration	366.48	332.84	486	297.71	265.96	323.16	0.045915207	0.954084793	0.707657957
MMAR_1314	MMAR_1314-1	intermediary metabolism, and respiration	43.92	36.64	36.27	51.15	64.52	74.25	0.03807203	0.96192797	1.525810033
MMAR_1375	atsD_1	intermediary metabolism, and respiration	82.2	75.61	71.24	116.77	99.98	97.83	0.045105339	0.954894661	1.286877663
MMAR_1644	MMAR_1644-1	intermediary metabolism, and respiration	328.41	302.83	296.61	515.76	483.78	493.42	0	1	1.507617966
MMAR_1693	MMAR_1693-1	intermediary metabolism, and respiration	39.01	49.63	85.59	135.38	129.54	119.42	0.000596132	0.999403868	2.090705548
MMAR_1710	ilvB1	intermediary metabolism, and respiration	401.84	402.4	419.84	285.7	309.38	360.7	0.015443336	0.984556664	0.732677734
MMAR_1712	ilvC	intermediary metabolism, and respiration	1408.01	1148.12	1404.58	928.08	992.27	1147.89	7.78E-08	0.999999922	0.729446702
MMAR_1749	pca	intermediary metabolism, and respiration	471.58	414.12	465.51	431.64	391.66	415.3	1.44E-07	0.999999856	0.863880057
MMAR_1821	amiC	intermediary metabolism, and respiration	221.55	161.31	206.88	138.12	121.85	141.73	0	1	0.643449311
MMAR_1926	pepR	intermediary metabolism, and respiration	765.21	660.46	660.41	499.46	469.16	447.93	1.59E-06	0.99999841	0.636809545
MMAR_1988	hflX	intermediary metabolism, and respiration	84.02	71.13	92.6	153.15	124.4	155.66	1.22E-15	1	1.648181308
MMAR_2202	gmk	intermediary metabolism, and respiration	983.53	802.32	1012.19	719.47	634.34	817.34	4.37E-08	0.999999956	0.725243021
MMAR_2269	csd	intermediary metabolism, and respiration	379.98	404.75	375.42	207.92	216.01	197.04	9.94E-10	0.999999999	0.499763131
MMAR_2277	trxB1	intermediary metabolism, and respiration	193.58	300.27	245.59	860.25	1155.43	1450.69	5.73E-09	0.999999994	4.267860008
MMAR_2333	wcaA	intermediary metabolism, and respiration	359.19	339.75	421.97	367.55	309.59	303.59	0.046088902	0.953911098	0.821013897
MMAR_2353	MMAR_2353-1	intermediary metabolism, and respiration	73.11	77.87	89.74	71.38	56.7	56.55	0.004733885	0.995266115	0.718241746
MMAR_2403	MMAR_2403-1	intermediary metabolism, and respiration	207.6	126.36	180.33	142.64	117.04	122.58	0.042975788	0.957024212	0.703990195
MMAR_2414	trpB	intermediary metabolism, and respiration	1215.91	1114.09	1302.09	1192.22	1179.27	1160.65	0.02051734	0.97948266	0.914596557
MMAR_2434	coaE	intermediary metabolism, and respiration	1131.25	878.05	940.89	642.04	561.88	639.31	4.42E-09	0.999999996	0.586881334
MMAR_2557	MMAR_2557-1	intermediary metabolism, and respiration	38.49	41.15	36.95	85.99	83.87	68.62	2.97E-09	0.999999997	1.906886499
MMAR_2568	MMAR_2568-1	intermediary metabolism, and respiration	74.11	68.53	70.63	94.53	99.05	88.06	0.024291682	0.975708318	1.242559235
MMAR_2570	MMAR_2570-1	intermediary metabolism, and respiration	38.32	28.89	34.79	52.92	45.52	68.44	0.035879997	0.964120003	1.537574015
MMAR_2775	MMAR_2775-1	intermediary metabolism, and respiration	13.64	15.52	16.12	32.67	20.66	37.29	0.022983869	0.977016131	1.859229408
MMAR_2836	MMAR_2836-1	intermediary metabolism, and respiration	27.07	19.86	28.89	67.28	60.7	65.19	0	1	2.417137656
MMAR_2844	MMAR_2844-1	intermediary metabolism, and respiration	269.04	263.12	308.64	142.72	169	207.84	6.87E-09	0.999999993	0.58121169
MMAR_2902	MMAR_2902-1	intermediary metabolism, and respiration	276.58	222.24	325.24	189.1	168.52	180.36	5.21E-11	1	0.615564306
MMAR_2992	folE_1	intermediary metabolism, and respiration	217.31	303.16	212.17	552.62	478.49	455.39	4.18E-06	0.999995822	1.861731257
MMAR_3294	glnA2	intermediary metabolism, and respiration	713.46	742.99	730.27	403.21	501.51	535.16	0.000194932	0.999805068	0.6165771
MMAR_3299	panB	intermediary metabolism, and respiration	277.79	406.1	349.53	144.11	173.39	203.42	0.013444654	0.986555346	0.466503511
MMAR_3555	MMAR_3555-1	intermediary metabolism, and respiration	1190.35	1568.78	741.53	84.21	149.32	100.74	0.000476253	0.999523747	0.087952775
MMAR_3556	MMAR_3556-1	intermediary metabolism, and respiration	241.54	232.92	117.7	11.97	13.82	14.61	4.02E-06	0.999995984	0.063121154
MMAR_3558	MMAR_3558-1	intermediary metabolism, and respiration	1156.85	1158.12	497.43	56.58	73.18	67.86	0.000579964	0.999420036	0.064837573
MMAR_3690	mbtF	intermediary metabolism, and respiration	42.7	34.26	37.16	67.12	52.76	51.03	0.008843736	0.991156264	1.411797101
MMAR_3691	mbtE	intermediary metabolism, and respiration	44.91	32.78	31.78	71.2	66.95	107.29	0.005040434	0.994959566	2.111062549
MMAR_3693	MMAR_3693-1	intermediary metabolism, and respiration	218.94	248.28	176.46	421.16	408.51	575.39	0.000161951	0.999838049	2.011405323
MMAR_3697	mbtG	intermediary metabolism, and respiration	204.69	244.69	173.02	386.85	372.31	524.11	0.001259956	0.998740044	1.906252317
MMAR_3701	MMAR_3701-1	intermediary metabolism, and respiration	22.49	21.82	25.86	46.72	56.37	40.53	0.000110864	0.999889136	1.924427421
MMAR_3702	MMAR_3702-1	intermediary metabolism, and respiration	15.95	18.99	18.02	31.41	36.65	45.27	1.53E-05	0.999984722	1.997134706
MMAR_3757	MMAR_3757-1	intermediary metabolism, and respiration	168.51	195.96	203.91	265.39	255.94	253.54	0.027080335	0.972919665	1.276646502
MMAR_3765	obg	intermediary metabolism, and respiration	419.19	243.44	344.49	219.96	185.32	232.88	0.005596786	0.994403214	0.601291182
MMAR_3801	mobA	intermediary metabolism, and respiration	181.58	231.91	270.96	98.65	109.84	172.12	0.014608792	0.985391208	0.516995816
MMAR_3802	porB	intermediary metabolism, and respiration	601.22	699.14	855.59	317.04	374.12	517.96	0.002643476	0.997356524	0.525970948
MMAR_3803	porA	intermediary metabolism, and respiration	582.12	801.23	848.31	313.24	371.46	421.94	0.000519218	0.999480782	0.463841671
MMAR_3813	rpiB	intermediary metabolism, and respiration	1630.51	1549.65	1608.93	1357.5	1163.33	1182.99	9.39E-08	0.999999906	0.71524519
MMAR_4102	thrC	intermediary metabolism, and respiration	408.38	301.07	382.41	312.3	279.21	329.96	0.000857123	0.999142877	0.79562298
MMAR_4135	MMAR_4135-1	intermediary metabolism, and respiration	569.51	723.13	845.49	1441.92	1460.45	1321.02	8.52E-12	1	1.830575834
MMAR_4180	MMAR_4180-1	intermediary metabolism, and respiration	340.41	322.53	321.16	431.71	398.6	414.73	0.015006896	0.984993104	1.183061222
MMAR_4252	putA	intermediary metabolism, and respiration	3.77	4.88	6.77	9.73	18.07	14.58	0.036257355	0.963742645	2.592969094
MMAR_4273	MMAR_4273-1	intermediary metabolism, and respiration	341.8	335.63	397.08	296.87	299.32	336.13	0.009525591	0.990474409	0.814501375
MMAR_4279	fbiC	intermediary metabolism, and respiration	161.51	132.57	157.86	231.21	205.21	216.38	0	1	1.363419676
MMAR_4372	phoH2	intermediary metabolism, and respiration	185.92	164.44	224.08	342.21	327.42	257.6	0.026236	0.973764	1.525238557
MMAR_4409	MMAR_4409-1	intermediary metabolism, and respiration	409.85	372.59	419.33	707.63	649.24	517.19	0.021500251	0.978499749	1.461410547
MMAR_4410	aslA	intermediary metabolism, and respiration	429.71	472.68	498.55	880.53	909.21	736.27	1.47E-06	0.999998526	1.690536065
MMAR_4473	pth	intermediary metabolism, and respiration	654	578.95	754.36	346.71	409.04	528.53	6.75E-06	0.999993246	0.604813991
MMAR_4497	MMAR_4497-1	intermediary metabolism, and respiration	37.82	32.63	38.57	222.1	202.36	161.31	0	1	5.033570801
MMAR_4614	MMAR_4614-1	intermediary metabolism, and respiration	126.38	108.53	121.73	190.05	196.27	154.66	0.004323493	0.995676507	1.429423378
MMAR_4725	MMAR_4725-1	intermediary metabolism, and respiration	22.93	26.14	22.19	40.31	51.57	39.15	0.003729951	0.996270049	1.714467152
MMAR_4762	cyp105Q4	intermediary metabolism, and respiration	8.33	9.85	9.68	17.4	24.57	27.89	8.38E-05	0.999916195	2.343934386
MMAR_4768	cysK2	intermediary metabolism, and respiration	26.98	26.09	16.59	69.82	68.16	144.53	0.016362205	0.983637795	3.748335275
MMAR_4784	MMAR_4784-1	intermediary metabolism, and respiration	655.33	685.04	1017.84	1250.46	1222.18	1279.88	0.043992951	0.956007049	1.499505059
MMAR_4869	sseC2	intermediary metabolism, and respiration	488.17	530.99	603.02	918.84	790.32	714.72	0.010352294	0.989647706	1.365264037
MMAR_5104	panD	intermediary metabolism, and respiration	929.29	1138.28	1220.04	621.62	637.84	656.81	7.34E-08	0.999999927	0.537547943
MMAR_5105	panC	intermediary metabolism, and respiration	254.77	237.19	282.17	168	144.82	168.62	0	1	0.582897299
MMAR_5169	MMAR_5169-1	intermediary metabolism, and respiration	278.58	287.01	289.52	410.51	389.87	381.02	6.89E-06	0.999993107	1.291421865
MMAR_5333	MMAR_5333-1	intermediary metabolism, and respiration	178.22	147.12	202.39	165.34	144.49	150.41	0.006361835	0.993638165	0.823237038
MMAR_5353	MMAR_5353-1	intermediary metabolism, and respiration	446.83	352.01	455.38	308.41	313.99	370.25	7.62E-07	0.999999238	0.744051559
MMAR_5394	glpQ1	intermediary metabolism, and respiration	320.28	272.19	387.63	205.96	223.17	205.14	1.63E-10	1	0.610740895

Lipid metabolism - 36 genes

GENE ID	GENE NAME	FUNCTION	FPKM Fragments Per Kilobase of transcript per Million mapped reads. NORMAL Long			LSMMG Long			PPEE	PPDE	RealFC
									posterior probability that a transcript is equally expressed	posterior probability that a transcript is differentially expressed	real fold change is the ratio of the normalized mean count values for LSMMG over the normalized mean count values for normal
			4days 35hr	4days 36hr	4days 37hr	4days 35hr	4days 36hr	4days 37hr			
MMAR_0336	desA3_2	lipid metabolism	536.96	427.89	199.01	1566.49	1873.72	1124.04	2.00E-06	0.999997996	3.642602765
MMAR_0452	fadD4	lipid metabolism	184.2	191.01	224.91	131.61	136.29	145.83	7.05E-10	0.999999999	0.648063347
MMAR_0466	MMAR_0466-1	lipid metabolism	30.21	24.8	23.46	45.8	44.63	34.57	0.037716856	0.962283144	1.495533171
MMAR_0505	fadE5	lipid metabolism	616.17	817.57	1107.22	289.32	364.97	389.88	0.002419515	0.997580485	0.387481623
MMAR_0706	fadD30	lipid metabolism	139.76	118.32	124.55	102.92	99.06	91.56	3.09E-05	0.999969058	0.721757377
MMAR_0793	fadB2	lipid metabolism	453.04	581.71	679.78	242.89	273.24	320.54	1.29E-05	0.999987083	0.455910375
MMAR_1154	lpqD	lipid metabolism	162.08	210.92	241.82	368.26	379.12	295.21	0.043198966	0.956801034	1.58020147
MMAR_1250	MMAR_1250-1	lipid metabolism	10.89	6.91	11.35	5.64	5.52	7.79	0.010247034	0.989752966	0.621407258
MMAR_1315	desA3	lipid metabolism	167.47	121.87	79.31	210.33	257.76	236.06	0.013707607	0.986292393	1.786156672
MMAR_1509	fadE23	lipid metabolism	301.98	283.58	351.31	577.35	663.63	691.17	0	1	1.942842229
MMAR_1761	fadD22	lipid metabolism	868.77	844.64	928.16	784.37	701.23	702.79	5.30E-07	0.99999947	0.778317897
MMAR_1762	pks15/1	lipid metabolism	423.34	376.72	537.39	311.02	294.39	326.72	3.53E-05	0.99996467	0.661088635
MMAR_1772	ppsE	lipid metabolism	439.78	392.21	385.76	289.2	266.48	239.8	3.53E-06	0.999996473	0.614338196
MMAR_1916	pptII	lipid metabolism	295.96	255.66	264.8	214.9	209.68	177.99	8.71E-05	0.999912858	0.68881433
MMAR_2117	fadD9	lipid metabolism	104.19	97.83	112.95	142.71	157.51	187.01	0.001643775	0.998356225	1.458862323
MMAR_2759	echA8_6	lipid metabolism	69.05	45.89	61.69	103.04	92.13	79.7	0.04558198	0.95441802	1.466677763
MMAR_2936	fadD9_1	lipid metabolism	10.5	4.55	7.5	22.1	21.25	29.53	1.03E-11	1	3.09817775
MMAR_2981	fabG3_1	lipid metabolism	80.12	62.38	77.18	153.17	131.86	106.79	0.000553164	0.999446836	1.674953012
MMAR_3336	fabD	lipid metabolism	635.63	869.97	1049.36	480.73	424.55	407.45	0.002034913	0.997965087	0.479730319
MMAR_3338	kasA	lipid metabolism	1573.14	2092.64	2899.76	747.93	795.98	1026.96	0.004804236	0.995195764	0.368151227
MMAR_3339	kasB	lipid metabolism	1248.25	1486.74	2124.87	549.44	593.68	746.87	0.000178132	0.999821868	0.3664396
MMAR_3340	accD6	lipid metabolism	540.4	799.22	1076.41	251.85	278.36	397.47	0.024221687	0.975778313	0.360483927
MMAR_3694	mbtC	lipid metabolism	101.24	79.05	59.67	161.79	124.04	217.13	0.032679826	0.967320174	1.951597776
MMAR_4030	MMAR_4030-1	lipid metabolism	108.31	132.99	110.52	224.87	227.3	249.15	2.23E-13	1	1.849925185
MMAR_4031	MMAR_4031-1	lipid metabolism	62.41	70.35	66.86	126.23	104.48	134.47	1.09E-09	0.999999999	1.702509486
MMAR_4032	MMAR_4032-1	lipid metabolism	34.45	35.61	34.69	73.72	72.31	73.66	6.13E-06	0.999993873	1.95944355
MMAR_4033	fab	lipid metabolism	72.2	70.27	68.83	103.54	148.22	132.05	0.002022377	0.997977623	1.696803576
MMAR_4476	pks16	lipid metabolism	335.12	428.78	458.8	174.88	213.4	242.34	2.48E-05	0.999975238	0.482838698
MMAR_4534	accA2	lipid metabolism	19.44	25.35	27.5	12.36	16.13	15.79	0.005373085	0.994626915	0.574627099
MMAR_4535	fadE12	lipid metabolism	52.87	43.44	68.84	34.17	32.63	34.62	2.66E-05	0.9999734	0.581963699
MMAR_4676	fadB	lipid metabolism	375.65	387.37	531.29	227.93	261.73	317.36	0.005715207	0.994284793	0.589325262
MMAR_4691	echA8_2	lipid metabolism	288.44	265.22	260.35	204.79	212.49	227.69	0.023551829	0.976448171	0.738925902
MMAR_5236	MMAR_5236-1	lipid metabolism	270.85	308.25	319.6	451.88	410.54	399.26	0.005027144	0.994972856	1.311841426
MMAR_5275	fadD15_1	lipid metabolism	218.37	205.96	238.56	309.25	284.95	318.62	0	1	1.295070885
MMAR_5364	pks13	lipid metabolism	375.79	409.53	410.43	232.69	230.13	266.34	1.68E-08	0.999999983	0.571739827
MMAR_5365	fadD32	lipid metabolism	637.7	754.41	715.53	409.87	410.83	427.95	1.42E-05	0.999985813	0.553160388

PE/PPE - 8 genes

GENE ID	GENE NAME	FUNCTION	FPKM Fragments Per Kilobase of transcript per Million mapped reads. NORMAL Long			LSMMG Long			PPEE	PPDE	RealFC
									posterior probability that a transcript is equally expressed	posterior probability that a transcript is differentially expressed	real fold change is the ratio of the normalized mean count values for LSMMG over the normalized mean count values for normal
			4days 35hr	4days 36hr	4days 37hr	4days 35hr	4days 36hr	4days 37hr			
MMAR_0641	MMAR_0641-1	PE/PPE	18.49	13.01	15.19	38.71	38.86	62.54	9.84E-05	0.99990155	2.839722433
MMAR_0761	MMAR_0761-1	PE/PPE	136.24	132.34	153.69	130.03	121.22	108.89	0.004837191	0.995162809	0.801774852
MMAR_2591	MMAR_2591-1	PE/PPE	28.92	32.99	41.27	22.75	22.45	23.96	0.001398129	0.998601871	0.632025436
MMAR_3443	MMAR_3443-1	PE/PPE	79.98	101.47	107.52	40.87	60.12	58.32	0.000188664	0.999811336	0.516249998
MMAR_4561	MMAR_4561-1	PE/PPE	11.32	4.68	12.05	28.91	20.42	27.45	1.58E-08	0.999999984	2.634624715
MMAR_4562	MMAR_4562-1	PE/PPE	52.14	31.7	73.45	131.93	116.14	135.65	2.43E-10	1	2.341039958
MMAR_4899	MMAR_4899-1	PE/PPE	62.55	70.24	43.86	155.03	115.61	108.02	0.00067088	0.99932912	1.974062812
MMAR_5448	MMAR_5448-1	PE/PPE	599.5	563.85	645.48	805.73	734.17	730.84	1.74E-05	0.999982579	1.177719623

Regulatory proteins - 16 genes

GENE ID	GENE NAME	FUNCTION	FPKM Fragments Per Kilobase of transcript per Million mapped reads. NORMAL Long			LSMMG Long			PPEE	PPDE	RealFC
									posterior probability that a transcript is equally expressed	posterior probability that a transcript is differentially expressed	real fold change is the ratio of the normalized mean count values for LSMMG over the normalized mean count values for normal
			4days 35hr	4days 36hr	4days 37hr	4days 35hr	4days 36hr	4days 37hr			
MMAR_0016	pknB	regulatory proteins	285.3	210.87	260.69	227.14	203.84	229.66	0.007193237	0.992806763	0.824932412
MMAR_0108	MMAR_0108-1	regulatory proteins	88.52	69.91	91.18	41.01	42.52	67.17	0.000371183	0.999628817	0.565530357
MMAR_0640	hspR	regulatory proteins	143.87	132.03	164.5	632.07	579.44	858.82	1.04E-14	1	4.348963467
MMAR_0790	MMAR_0790-1	regulatory proteins	37.28	36.01	41.22	18.71	24.25	30.43	0.000219013	0.999780987	0.602912962
MMAR_1132	whiB3	regulatory proteins	346.95	402.9	407.3	117.55	115.23	242.44	4.65E-07	0.999999535	0.376107149
MMAR_1242	MMAR_1242-1	regulatory proteins	222.43	183.58	147.49	312.46	356.38	446.44	0.000928408	0.999071592	1.857277355
MMAR_1365	whiB7	regulatory proteins	111.73	70.4	65.32	186.01	180.87	336.32	0.048463671	0.951536329	2.59804091
MMAR_2281	MMAR_2281-1	regulatory proteins	156.82	156.08	234.79	121.59	100.86	125.7	0.007252402	0.992747598	0.594940856
MMAR_3454	MMAR_3454-1	regulatory proteins	218.11	166.2	235.56	118.48	156.64	181.15	0.034698044	0.965301956	0.69130916
MMAR_3703	MMAR_3703-1	regulatory proteins	109.01	96.59	107.89	190.25	289.87	408.62	0.04859521	0.95140479	2.662893181
MMAR_4325	MMAR_4325-1	regulatory proteins	75.19	70.09	65.41	115.86	104.78	107.42	7.33E-09	0.999999993	1.455162734
MMAR_4577	pknD	regulatory proteins	51.19	50.84	62.13	106.01	90.83	78.13	0.000953268	0.999046732	1.577430885
MMAR_4942	phoP	regulatory proteins	954.79	1013.72	1031.12	677.22	749.76	791.11	0.000182649	0.999817351	0.688698928
MMAR_5069	MMAR_5069-1	regulatory proteins	86.84	90.51	83.35	42.42	29.74	56.21	1.18E-05	0.999988156	0.454591254
MMAR_5170	whiB4	regulatory proteins	352.49	412.02	348.95	925.23	737.43	700.07	7.43E-09	0.999999993	1.931346496
MMAR_5405	ethR	regulatory proteins	269.76	346.71	315.26	115.41	85.47	149.99	2.72E-08	0.999999973	0.347071577

Virulence, adaptation and detoxification - 5 genes

GENE ID	GENE NAME	FUNCTION	FPKM Fragments Per Kilobase of transcript per Million mapped reads. NORMAL Long			LSMMG Long			PPEE	PPDE	RealFC
									posterior probability that a transcript is equally expressed	posterior probability that a transcript is differentially expressed	real fold change is the ratio of the normalized mean count values for LSMMG over the normalized mean count values for normal
			4days 35hr	4days 36hr	4days 37hr	4days 35hr	4days 36hr	4days 37hr			
MMAR_0515	hsp	virulence, adaptation and detoxification	10.21	10.08	12.69	250.44	285.9	913	0.001546827	0.998453173	40.77169742
MMAR_0637	dnaK	virulence, adaptation and detoxification	620.89	802.5	920.1	2607.47	4190.37	6636.63	0.043718887	0.956281113	5.377890602
MMAR_3740	eis	virulence, adaptation and detoxification	18.46	14.1	19.5	42.16	44.17	50.55	0.000124326	0.999875674	2.487015373
MMAR_3776	rpfE	virulence, adaptation and detoxification	272.23	197.58	171.44	132.01	61.99	90.14	0.024588876	0.975411124	0.410842249
MMAR_4053	mce3C_1	virulence, adaptation and detoxification	43.81	54.1	61.29	90.7	80.11	75.55	0.011300324	0.988699676	1.447590937
